# Modeling of xenobiotic transport and metabolism in virtual hepatic lobule models

**DOI:** 10.1371/journal.pone.0198060

**Published:** 2018-09-13

**Authors:** Xiao Fu, James P. Sluka, Sherry G. Clendenon, Kenneth W. Dunn, Zemin Wang, James E. Klaunig, James A. Glazier

**Affiliations:** 1 Biocomplexity Institute, Indiana University, Bloomington, IN, United States of America; 2 Department of Physics, Indiana University, Bloomington, IN, United States of America; 3 Department of Intelligent Systems Engineering, Indiana University, Bloomington, IN, United States of America; 4 School of Medicine, Indiana University, Indianapolis, IN, United States of America; 5 School of Public Health, Indiana University, Bloomington, IN, United States of America; Laval University, CANADA

## Abstract

Computational models of normal liver function and xenobiotic induced liver damage are increasingly being used to interpret *in vitro* and *in vivo* data and as an approach to the *de novo* prediction of the liver’s response to xenobiotics. The microdosimetry (dose at the level of individual cells) of xenobiotics vary spatially within the liver because of both compound-independent and compound-dependent factors. In this paper, we build model liver lobules to investigate the interplay between vascular structure, blood flow and cellular transport that lead to regional variations in microdosimetry. We then compared simulation results obtained using this complex spatial model with a simpler linear pipe model of a sinusoid and a very simple single box model. We found that variations in diffusive transport, transporter-mediated transport and metabolism, coupled with complex liver sinusoid architecture and blood flow distribution, led to three essential patterns of xenobiotic exposure within the virtual liver lobule: (1) lobular-wise uniform, (2) radially varying and (3) both radially and azimuthally varying. We propose to use these essential patterns of exposure as a reference for selection of model representations when a computational study involves modeling detailed hepatic responses to xenobiotics.

## Introduction

“Virtual Tissues” are an expanding class of multi-scale quantitative mechanism-based models designed to predict the complex interactions between subcellular biochemical networks, cell-level behaviors, tissues, organs and the body as a whole [1–7]. Virtual tissues provide a trans-disciplinary approach to capturing and integrating knowledge and expertise in specific organ systems, biology, clinical practice, biological big data, modeling and software development. Here we compare and contrast three virtual-tissue models of the liver.

The liver’s physiological functions include production of bile, synthesis of clotting factors and hormones, metabolism of nutrients, storage and release of iron, vitamins and glycogen, and detoxification of xenobiotics. The liver contains many cell types but the major parenchymal cell, the hepatocyte, makes up most of the liver’s mass and is responsible for the majority of its metabolic functions. Sinusoidal endothelial cells line the liver sinusoids (capillaries), fenestrated channels that facilitate molecular exchange between blood and the liver parenchyma. In sinusoids, the space between the abluminal surface of the endothelial cells and the parenchyma, the Space of Disse, replaces the normal vasculature’s basement membrane.

**Fig 1:**
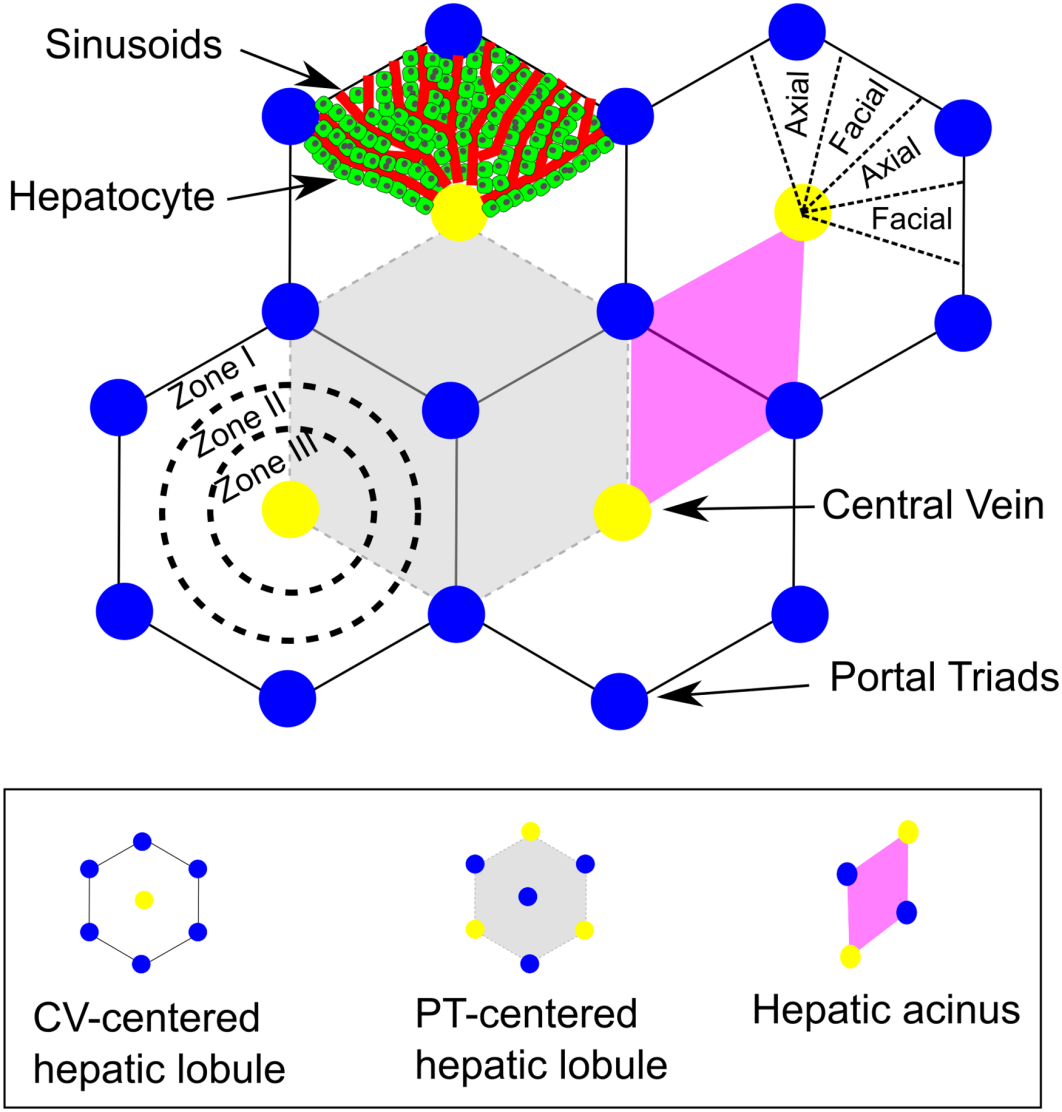
Schematic views of the hepatic lobule. Three classic schematics of the liver lobule are; central vein (CV)-centered, portal triads (PT)-centered and acinus. Despite different descriptive views of a liver lobule, the components and functions are the same. PT (blue) consists of hepatic artery, portal vein and bile duct. Blood enters a liver lobule via both hepatic arteries and portal veins, flows across network of liver sinusoids (red), and empties into central vein (CV). During transit, blood-borne substances are absorbed by and metabolized within hepatocytes (green), the major parenchymal cells of the liver, which make up most of liver mass. A liver lobule has three sub-regions (zones) that carry out different metabolic functions. Although definitions for zones vary slightly dependent on the descriptive view, generally, zone I refers to the periportal region; zone II refers to mid-lobular region; zone III refers to pericentral region. Due to both local local microdosimetry and different metabolic functions, the three zones exhibit different types and extents of damage under pathological conditions.

Anatomically, the human liver consists of millions of repeated functional units called lobules. The lobules connect to the liver vasculature in a parallel fashion so that a portion of blood (e.g., a red blood cell) passes through exactly one lobule as it transits the liver. As shown in Fig 1, basic models of the repeat unit of the liver include: (1) the central vein (**CV**)-centered liver lobule, (2) the portal triad (**PT**)-centered lobule and (3) the hepatic acinus (after [8] and [9]). These are different ways to look at the liver lobule, but the liver functions the same regardless of which of the viewpoints is used. In general, a lobule consists of a single centrally located CV and three to six PT blood sources located at the vertexes of a roughly hexagonal region centered at the CV. Each PT contains an artery, vein and bile duct. The CV drains the blood the PTs supply to the sinusoids. Fig 1 assumes a regular hexagonal lobule with six PTs per lobule, the “classical” lobule caricature. The PT-centered liver lobule has a basic unit of a central PT supplying blood to three CVs. If properly implemented either of these caricatures can construct a liver lobule in which blood flows from the hepatic artery and portal veins (which delivers venous blood from the small intestine) of the PT, across a complex network of sinusoids draining into the central vein. In the CV-centered liver lobule, blood flow “converges” from peripheral to central regions. In contrast, in the PT-centered model, blood flow “diverges” from the PTs. In this work, we use the CV-centered (classical) representation of the liver lobule.

Many metabolic functions of the liver occur within specific subregions, termed zones, in the lobule [10]. These zones are typically not apparent in standard histological sections. That is, no visible boundaries define the locations of individual zones. To observe zones in liver-tissue sections requires either staining for zonally restricted proteins or observing localized tissue changes such as necrosis following exposure to a liver toxicant. Zones visualized in tissue sections usually appear as roughly concentric rings centered on either the CV or a PT. In general, the lobule is defined as having three zones; Zone I is periportal and includes the PTs and post-terminal hepatic venules; Zone III is the region around the CV; Zone II includes the region intermediate between the periportal and pericentral regions [8] (Fig 1). Zone I is primarily responsible for glucose metabolism and ammonia detoxification processes. Zone III carries out most lipogenesis and glutamine synthesis and also expresses high levels of xenobiotic metabolizing cytochrome P450s such as CYP1A2 and CYP2E1. Zone II is a transitional region whose capabilities are generally not characterized but presumably has capabilities intermediate between those of Zone I and Zone III.

The liver is the primary site of metabolism and clearance of xenobiotics from the blood. In some cases, hepatocyte metabolism produces toxic metabolites that damage or kill hepatocytes. If the xenobiotic is a pharmaceutical, we refer to xenobiotic induced damage as Drug Induced Liver Injury (DILI), which is a significant cause of failure for new drugs [11]. Often DILI is zonal rather than uniform across a lobule. For example, in mice, acetaminophen overdose causes centri-lobular necrosis (Zone III) [12–14] (see the H&E sections in Section 1.1 in S1 Text). This zonal damage may result from high expression of Cytochrome P450 enzymes in Zone III hepatocytes. The underlying cause of zonal differences within the lobule is not clear. The decreasing blood oxygen concentration from the periportal to pericentral regions may allow individual hepatocytes to determine their position along the portal to central axis, hence their zonal position, and to change their behavior accordingly [15, 16]. Wnt/*β*-catenin signaling is also involved in liver zonation [17, 18], but the detailed control mechanisms are not clear. Other factors that may contribute to the zonation signal include the variable shear forces, or variable hepatocyte ages, across the lobule.

To understand the underlying cause(s) of lobule zonation, including xenobiotic-induced liver damage, it is important to investigate how cellular exposures to xenobiotic varies with location within the lobule. Understanding the microdosimetry (dose at the level of individual cells) is key to understanding the zonal distribution of damage [19] as well as the basic development of the zones themselves. In this work we address two questions key to understanding and modeling the functioning of, and damage to, the liver lobule. First, is the complex lobular blood flow pattern sufficient to explain zonal exposure and damage? Second, what characteristics of a xenobiotic make detailed lobule flow modeling, in a spatially accurate lobule model, a requirement to predict the pattern of exposure?

### Existing computational models

**Fig 2:**
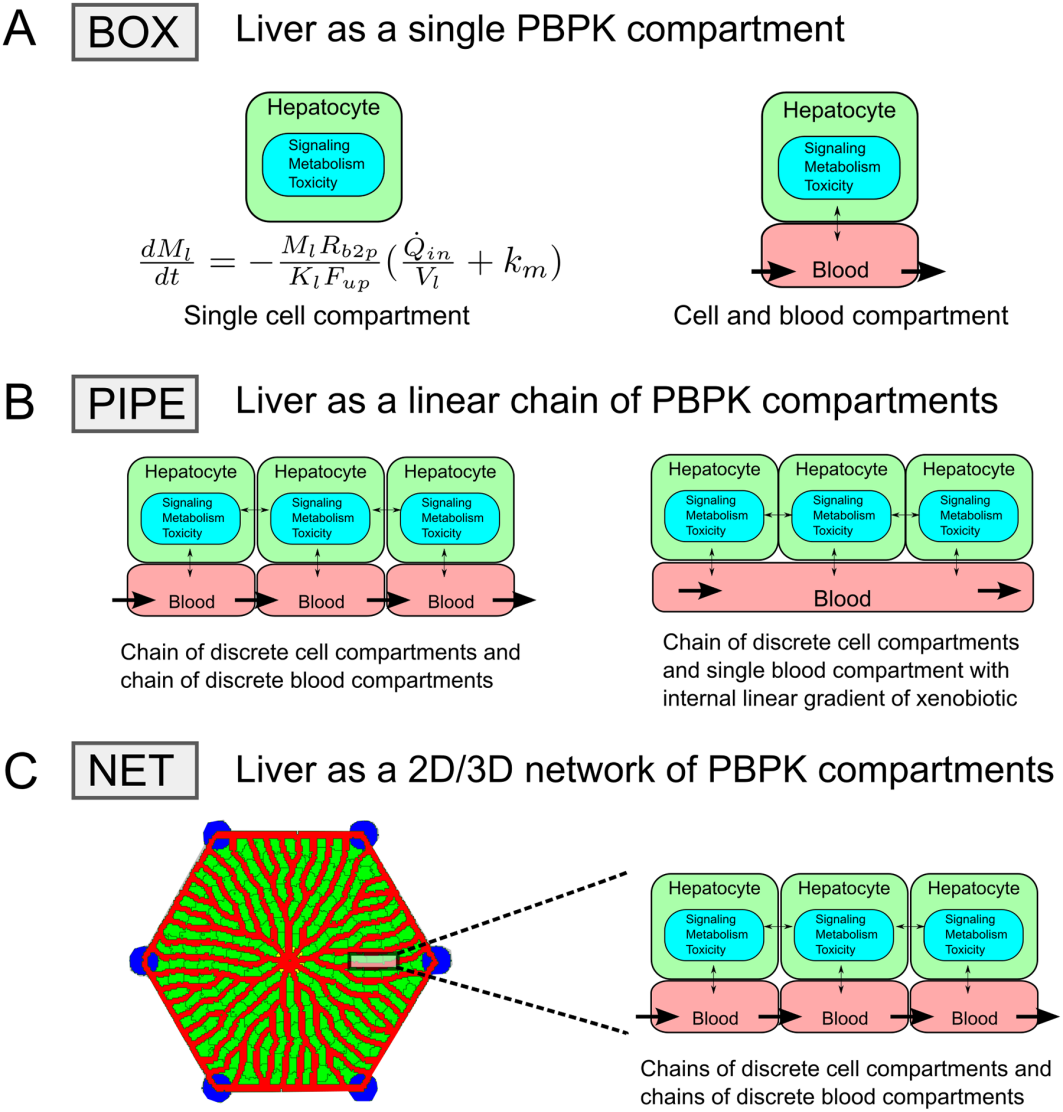
Schematics of model representations of the liver. Representations of the liver as **BOX** (A), **PIPE** (B), or **NET** (C) models. **BOX** models represent liver as either a single PBPK compartment representing both the blood and tissue of the organ (left in (A)) or separate blood and tissue compartments (right in (A)). **PIPE** models represent liver tissue as a linear chain of hepatocyte (or perhaps zonal) compartments and model blood as either a chain of blood compartments (left in (B)) or as a continuous medium for substance transport solved using convection-diffusion equation (right in (B)). **NET** models represent liver sinusoid network as spatially defined anastomotic chains of compartments and represent hepatocytes as individual compartments lining the sinusoid network (C).

A number of virtual tissue models have been developed to study liver physiology and injury as outlined in Fig 2. (1) The coarsest resolution represents the liver as a single Physiologically Based Pharmacokinetic (PBPK) compartment (referred to here as a ***BOX***) with PBPK blood and tissue sub components, treated as a well-mixed compartments. (2) A simple zonation model represents an unbranched, hepatocyte-lined sinusoid (referred to here as a ***PIPE***) as a linear array of PBPK compartments (e.g., a string of ***BOX*** s), each containing well-mixed blood and cellular sub-compartments [1]. Compartments may represent individual hepatocytes or liver zones. (3) To simulate blood flow and microdosimetry in more detail, we present a detailed lobule as a 2D or 3D network of hepatocyte-lined sinusoids in an anastomotic network (referred to here as a ***NET***).

***BOX*** models (Fig 2A) of the liver suffice to characterize metabolism and signaling within the liver and blood levels of xenobiotics and xenobiotic metabolites, when the spatial variation of xenobiotics is insignificant or when the average response of the liver parenchyma to xenobiotics is sufficient [1, 12, 14, 20]. ***BOX*** models are computationally fast and integrate easily with whole-body PBPK models to describe absorption, distribution, metabolism and excretion (ADME) of xenobiotics. However, ***BOX*** models can not predict microdosimetry, spatial variations of metabolism or zonal necrosis within the liver lobule.

***PIPE*** models (Fig 2B) can model gradients of xenobiotics in the sinusoid and in the hepatocytes along a simulated hepatocyte-lined sinusoid [1, 13, 21–24]. ***PIPE*** models can include zonal expression of enzymes by fiat or based on some emergent gradient within the sinusoid, such as the oxygen concentration. A simple ***PIPE*** model uses sub-compartments to describe the advective movement of xenobiotics in blood and hepatocyte extraction [22]. More sophisticated ***PIPE*** models combine advection-diffusion equations for blood and blood-borne xenobiotics and transport flux equations for transfer between blood and tissue [1, 13, 21]. Therefore, ***PIPE*** models can model zonal and gradient differences but do not accurately capture the complex blood flow patterns, such as differences in blood velocity, and do not represent the complex blood flow resulting from the anastomotic sinusoid network of the lobule. In particular, ***PIPE*** models do not represent the true cellular partitioning of a lobule, which has many more peripheral versus pericentral hepatocytes and many more peripheral versus pericentral sinusoids (Fig 1).

An explicitly ***NET*** (Fig 2C) model of anastomotic network attempts to reproduce the detailed lobular sinusoid structure, and the emergent microcirculation and microdosimetry. Hoehme *et al*. have attempted a complete reconstruction of the 3D sinusoid architecture of a complete liver lobule and developed an agent-based model to simulate hepatocyte motility and mitosis during liver regeneration after centri-lobular necrosis induced by carbon tetrachloride (**CCl_4_**) [25]. Shliess *et al*. [26] and Ghallab *et al*. [27] extended Heohm *et al*. model by coupling metabolic models within lobular compartments (periportal, mid-zonal and pericentral) to a whole-body model in order to simulate the ADME of CCl_4_. However, these models did not characterize the complex blood flow rates and cellular exposure to xenobiotic within the simulated liver lobule.

Bonfiglio *et al*. [28] modeled lobular microcirculation by treating the sinusoid space as a 2D porous medium perfused by blood to investigate the steady state distribution of hemodynamic variables of blood pressure and velocity in a hexagonal liver lobule. A similar flow model has recently been reported by Ricken *et al*. [29]. Debbaut *et al*. [30] extended this approach by building a 3D based model with a vascular septum (vascular network that supplies blood along the edges of a lobule). Debbaut *et al*. also investigated the effect of anisotropic permeability, which showed more spatially homogeneous blood flow compared to their model with isotropic permeability. These continuous models provide insight into lobular hemodynamics. However, they do not include biological components such as blood vessels and cells, making it difficult to compare architectural features of these virtual lobules with experimental observations such as the volume of sinusoid per unit volume parenchymal. In addition, it’s difficult to incorporate into these models experimental information such as the zonal expression of enzymes or transporters.

Rezania *et al*. generated a hexagonal liver lobule model with a pseudo-random sinusoidal structure using a sequential diffusion-limited aggregation algorithm. They then simulated drug microdosimetry using a convection-diffusion-reaction flow model, and calculated oxygen distribution and oxygen-driven zonation of CYP enzymes [31]. However, their model did not have biologically described components and it is difficult to define properties for individual cells or sinusoid segments.

Hunt and coworkers developed a model that represents a liver sinusoid segment consisting of a core region and three concentric cylindrical grids around the core [32, 33]. In their sinusoid segment model, the core represents the blood space and the inner to outer grids represent sinusoid rim, endothelial layer and hepatocyte cell layer (including the space of Disse), respectively. Their constructed liver lobule is a topological anastomotic network of these sinusoid segments. They then simulated advection, diffusion and intracellular binding and metabolism of xenobiotics. Their model provided a general purpose framework for simulation of microdosimetry. However, they did not model the spatiality of a liver lobule, such as the detailed geometry of the sinusoids (diameter, direction, branch angle) or hepatocytes (cell size and shape, extent of contact with sinusoids).

Wambaugh and Shah developed a spatially explicit 2D model of the sinusoidal network in a hexagonal liver lobule. They then coupled their lobule model with a whole-body PBPK model to simulate the microdosimetry of xenobiotics [19]. Their model provided a basic microdosimetry modeling framework for the development of our model.

### Biological question

The key biological question in this study is: *how does the complex liver microcirculation of blood, that arise from the anastomotic microvascular network, affect the microdosimetry within the liver lobule?* There are two rationales for addressing this question:

1. Liver lobules have zonal or regional variation in many physiological functions including xenobiotic metabolism, but the origin of such zonal variation is poorly understood. One hypothesis is that blood-borne signal could contribute to regulation and maintenance of liver’s metabolic zonation. Thus, modeling small molecule or xenobiotic microdosimetry in the liver lobule could provide mechanistic evidence for this hypothesis.
2. Liver damage, such as APAP-induced liver necrosis, often shows a zonal pattern where hepatocytes in certain regions of the lobule are more susceptible to damage than others. A liver lobule model with detailed characterization of microvascular architecture can serve as a module of the tissue scale and can be integrated with cell and whole-body modules to form a multi-scale simulating framework that includes the spatio-temporal aspects of liver metabolism and xenobiotic damage.

Hypothetically, zonal variation in hepatic (liver) microdosimetry of xenobiotics could cause zonal damage, even without zonally dependent xenobiotic metabolism. This study investigates if the pattern of blood flow in the liver lobule, without zonal variation in hepatocyte transport and metabolism, is sufficient to create zonal hepatic exposure to xenobiotics that could lead to zonal damage. In addition, by studying the zonal microdosimetry of a xenobiotic obtained using a range of parameters (such as rate of uptake or metabolism) we should be able to define metrics to help modelers choose the appropriate level of detail for a particular modeling goal. In particular, what xenobiotic characteristics suggest the use of ***BOX*** versus ***PIPE*** versus ***NET*** representations. In other words, in what cases is detailed flow and transport modeling required in order to explain microdosimetry as opposed to when a simple flow model provides an adequate microdosimetry prediction. If the microdosimetry is constant across the lobule then there is no need to use a complex flow model.

## Materials and methods

### Construction of the virtual liver lobule

**Fig 3:**
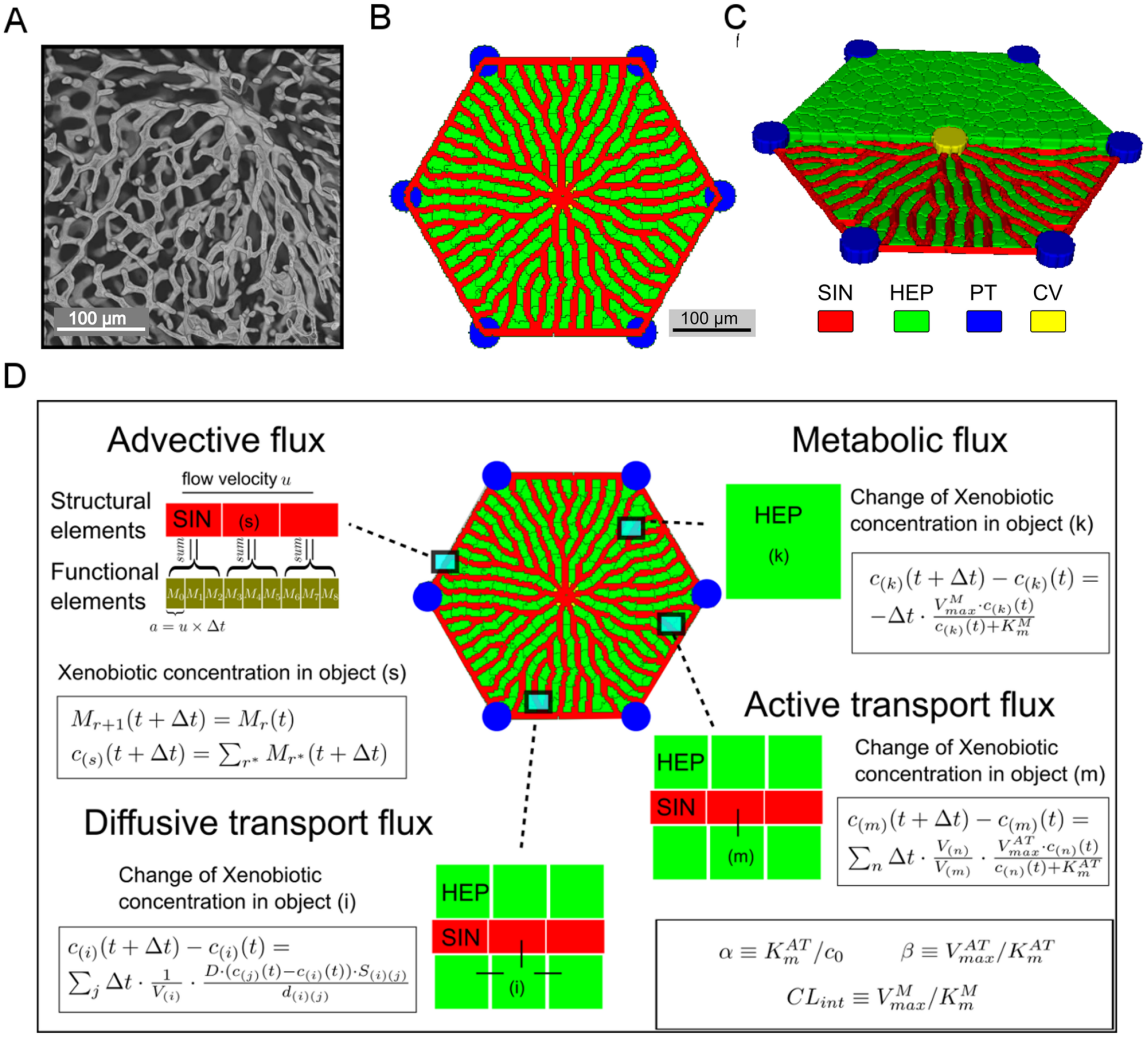
Model description. (A) Micrograph of sinusoids in a deep section of a rat liver. A central vein is located near the upper right. The scale bar is 100*μm* and the width of the individual sinusoids is ≈ 8*μm*. (B) Two-dimensional view of the virtual mouse liver lobule showing the sinusoid network. Hepatocytes, sinusoids, central vein and portal triads are colored green, red, yellow and blue, respectively. (C) Partial cut-away three-dimensional view of the virtual mouse liver lobule. (D) Schematics and equations for transport and metabolism. For more details on the advection model see Section 1.5 in S1 Text.

One representation of the liver is a ***PIPE*** virtual liver lobule, described in Section 1.3 in S1 Text and in our previous work [1]. In this study, we focus on describing a more realistic model representation of the liver—a ***NET*** virtual liver lobule. We constructed a virtual mouse liver lobule (Fig 3B and 3C) based on the analysis of rodent liver lobules by us (Fig 3A and Section 1.2 in S1 Text) and others [25]. A key observation is that in thick tissue sections individual hepatocytes are in contact with at least two sinusoids. In addition, we and others [25] have experimentally determined quantitative descriptions of rodent liver lobules including the volume fraction of sinusoids, average sinusoid-parenchyma interfacial area per unit parenchyma volume, branching angle and inter-branch segment length (Section 1.2 in S1 Text). These architectural quantities are criteria with which we compare our virtual liver lobules to real lobules.

The ***NET*** virtual liver lobule is a regular hexagon with CV at the center, six PTs at the vertices, and a dense network of sinusoids connecting the PTs and the CV. We used a set of simple rules to create the virtual sinusoid network as described in Section 1.4 in S1 Text. Briefly, the network is a collection of nodes (representing sinusoid junctions) and connecting edges (representing sinusoid segments). The first step was to determine the locations of nodes. We selected the center of the lobule hexagon as the root node (the location of the CV) and then placed concentric rings encircling the root node with radial spacing between each pair of rings equal to one hepatocyte width. The radius of the area enclosed by the first concentric ring equaled one hepatocyte size plus the radius of CV. We then divide the individual concentric rings along their perimeters every hepatocyte width placing nodes on each ring. The location of the first node on each ring was chosen randomly. We repeated this process of adding rings and subdividing out to the perimeter of the simulated lobule. Next, we connected nodes on each ring to the nearest node on the next ring outward to form an edge (a sinusoid segment). The root node connected to all nodes placed on the innermost ring, while the nodes on the outermost ring connected to the nearest nodes on the perimeter of the hexagon. Nodes did not connect to other nodes on the same ring. We then transformed the topological network into a spatial network in CompuCell3D (**CC3D**) [34, 35]. Along each edge, we created a sinusoid diameter cylinder to represent a sinusoid segment. To position the hepatocytes, we chose the voxel between two adjacent nodes on the same ring as the center of a hepatocyte and expanded this voxel to the proper cell volume, taking into account the cell-cell and cell-vessel interactions, in CC3D (see Fig 3B and 3C).

The constructed sinusoid network acquires a “radiating” anastomotic pattern from the root node (CV). Qualitatively, every hepatocyte is in contact with at least two sinusoids (hepatocyte at the periphery can contact more than two), and the spacing between adjacent pseudo-parallel sinusoids equals one hepatocyte width. The characteristics of the simulated liver lobule are given in (Table 1). The virtual liver lobule is three dimensional with a thickness of *h*_*lob*_ = 20 *μm* (one hepatocyte) and the bounding lobule hexagon edge length of *a*_*lob*_ = 200 *μm*, which is also the hexagon’s major radius. The constructed sinusoid network is planar and constrained to the center of this slab. The diameter of sinusoid segments is *d*_*sin*_ = 8*μm* everywhere in the simulated lobule. The complete virtual liver lobule slice contains 217 hepatocytes with mean hepatocyte volume of *V*_*hep*_ = 8030 *μm*^3^. Since the lobule’s sinusoid network is created with a stochastic algorithm, we examined a set of networks built with the same method (see Section 2.2 in S1 Text).

**Table 1:** Anatomical parameters of the simulated and real liver lobules.

Parameter	Description	Value	Source or Comparator
*h*_*lob*_	Thickness of constructed liver lobule slice	20 *μm*	Hepatocyte thickness 23.3 ± 3.1 *μm* [25]
*a*_*lob*_	Length of the perimeter edge (and major radius) of the constructed liver lobule slice	200 *μm*	284.3 ± 56.9 *μm* [25]
*d*_*sin*_	Diameter of sinusoidal lumen	8 *μm*	Sinusoid diameter including the thickness of the endothelial cell(s) 9.6 ± 4.5 *μm* [36]
*d*_*cv*_	Diameter of central vein	40 *μm*	41.2 ± 32.1 *μm* [25]^†^
*d*_*pt*_	Diameter of portal triad	40 *μm*	Assumed similar to *d*_*cv*_
${\bar{V}}_{hep}$	Mean hepatocyte volume in constructed lobule	8030 *μm*^3^	12, 653 ± 3915 *μm*^3^ [25], 5128 ± 838 *μm*^3^ [36]
*η*_*hep*_	Hepatocyte volume fraction in liver lobule volume excluding central vein and portal triads	87.1%	77.8 ± 1.15% [37]
*η*_*sin*_	Sinusoids volume fraction in liver lobule volume excluding central vein and portal triads	12.9%	13% (this work), 15.3 ± 3.9% [36], 10.6 ± 4.5% [37]
*S*_*int*_	Interfacial area between sinusoid and parenchymal space per unit parenchymal volume	0.122 *μm*^2^/*μm*^3^	0.163 ± 0.087 *μm*^2^/*μm*^3^ based on data in [36]
*θ*_*branch*_	Average minimum branching angle at sinusoid junctions	66.9° ± 12.8°	67.4° ± 27.56° (this work, from surface sections of mouse liver), 78.81° ± 22.60° (this work, from deep sections of rat liver in Fig 3A), 32.5° ± 11.2° reported in [25, 36]^‡^
*L*_*seg*_	Average inter-branch sinusoid length	51.0 ± 37.2 *μm*	43.1 ± 18.9 *μm* [25], 23.93 ± 5.9 *μm* [36]
Δ*P*	Pressure drop from portal triad to central vein	0.6 *mmHg*	See text.
*mpp*	Conversion factor for pixels to microns	2 *μm*/*px*	

^†^ [25] gives this value as the radius but examination of the paper’s figures suggests that the value is the diameter.

^‡^ [25] and [36] appear to be giving the half-angle.

The virtual liver lobule slice represents a minimal functional unit of a mouse liver across which a xenobiotic distributes. In a larger simulated tissue section there would be six additional units next to the simulated lobule as well as units stacked above and below the simulated lobule slice. In this study, the simulated liver lobule is isolated and independent such that no exchange of the xenobiotic occurs between the simulated liver lobule and the eight neighboring lobule slices.

One qualitative feature of the constructed virtual lobule is the uniform patterning of sinusoids, which is similar to what is see in mouse liver sections. We quantitatively compared structural properties of our simulated liver lobule with mouse data (Table 1). The volume fraction of sinusoids in the virtual lobule, excluding the CV and PT, is *η*_*sin*_ = 12.9%, comparable to experimentally measured values of 15% [36], 11% [37] and 10.4% ± 1.1% in our measurements in *ex vivo* rat lobules. *Ex vivo* sinusoid networks show a small variation in sinusoids diameters, with the largest diameters occurring near the PT and CV and smaller diameters in the mid-lobular region (see Section 1.2 in S1 Text). In addition, the *ex vivo* samples have a somewhat more densely connected network in three dimensions and it is common to observe nodes with four or more connections.

The average sinusoid-parenchyma interfacial area per unit parenchymal volume *S*_*int*_, which affects xenobiotic uptake by hepatocytes, is 0.122 *μm*^2^/*μm*^3^ in our virtual liver lobule versus 0.163 *μm*^2^/*μm*^3^ in [36] and 0.143 ± 0.019 *μm*^2^/*μm*^3^ from our measurements in *ex vivo* rat lobules.

Branching angle *θ*_*branch*_, which defines the smallest of the three inter-segment angles at bifurcations in the sinusoid network, is 66.9° ± 12.8° in the virtual liver lobule. Values measured by us are 67.4° ± 27.6° for surface section of mouse liver and 78.8° ± 22.6° for deep section of rat liver. Hoehme *et al*. [25] and Hammad *et al*. [36] reported a smaller value of 32.5° ± 11.2°, but appear to be referring to the branching half angle.

The average inter-branch sinusoid segment length *L*_*seg*_ is 51.0 ± 37.2 *μm* in our virtual liver lobule, larger than our measured value of 26.3 ± 17.4 *μm* and reported values, 43.1 ± 18.9 *μm* in [25] and 23.9 ± 5.9 *μm* in [36]. Our model assumption that the two-dimensional virtual sinusoid network has no inter-plane connections may be the source of the longer segment lengths in our virtual liver lobule versus real lobules. Overall, the virtual liver lobule matches many of the architectural aspects of the rodent liver summarized in Table 1.

The virtual liver lobule contains two types of computational objects; sinusoid segments (**SIN**s) and individual hepatocytes (**HEP**s). Biologically, a SIN represents a volume of blood. The sinusoidal endothelial cells and the Space of Disse, which represent relatively little volume, are not explicitly modeled. We treat the blood as homogeneous fluid and do not explicitly treat, or differentiate, between serum and blood cells such as erythrocytes (red blood cells). In addition, we do not explicitly model the separate arterial and venous blood inflows at the PT, the two blood flows are considered to have already mixed. Computationally, a SIN is a container of the xenobiotic dissolved in blood and is modeled as a CC3D pseudo-cell. HEPs are CC3D cells representing individual hepatocytes.

### Calculation of blood flow and velocity distributions

With the sinusoid network constructed, we calculated the blood flow velocity within each sinusoid segment using Hagen-Poiseuille’s law with mass conservation at sinusoid junctions (e.g., Kirchhoff’s Current Law). The Hagen-Poiseuille equation describes the pressure-velocity profile for laminar flow through a cylindrical tube, with the fluid characterized as incompressible and Newtonian. To apply this equation to blood flow within our microvascular network, an apparent viscosity dependent on vessel diameter is needed to describe the Fahraeus-Lindqvist effect and inverse Fahraeus-Lindqvist effect where resistance to blood flow decreases when vessel diameter drops from 200 *μm* to about 10 *μm* but increases when vessel diameter drops below about 10 *μm*. We used an empirical equation to describe the relationship between blood flow viscosity and vessel diameter in the microcirculation [38, 39] to calculate the blood flow rate ${\dot{Q}}_{ij}$ within a sinusoid segment *ij* using the Hagen-Poiseuille equation:

$$\begin{array}{c} \Delta P_{ij}=\frac{128\mu_{e}L_{ij}{\dot{Q}}_{ij}}{\pi d_{ij}^{4}} \end{array}$$ (1)

where *P*_*ij*_ is the pressure difference between two sinusoid junctions *i*, *j*; *μ*_*e*_ is the apparent blood viscosity (a function of vessel diameter and hematocrit, see Eq (6)-(7) in [39]); *L*_*ij*_ is the length of the sinusoid segment *ij*; ${\dot{Q}}_{ij}$ is the volumetric flow rate through the sinusoid segment *ij* and *d*_*ij*_ is the diameter of sinusoid segment *ij*.

To calculate blood flow rates across the network, we require conservation of volumetric blood flow rates at sinusoid junctions; the volume of blood flow entering a sinusoid junction equals that leaving the junction, namely $\Sigma {\dot{Q}}_{i}^{in}=\Sigma {\dot{Q}}_{i}^{out}$. In addition, we set a fixed pressure difference boundary condition Δ*P* between PT nodes and the CV node.

### Models of transport and metabolic fluxes

The virtual lobule model mechanistically characterizes transport and metabolic processes of the xenobiotic (Fig 3D), including:

1. Advective process: movement of the blood-borne xenobiotic along a chain of SINs that collectively build a sinusoid segment;
2. Diffusive (passive) transport process: bi-directional movement of the xenobiotic across the plasma membrane of adjacent HEPs and between adjacent HEPs and SINs (blood), and between adjacent SINs;
3. Active transport process: uni-directional movement of the xenobiotic into a HEP from adjacent SINs, mediated via inwardly rectified, saturable membrane transporters;
4. Metabolic process: metabolism of the xenobiotic within individual HEPs.

**Advective process:** To simulate the advective process of the xenobiotic, we used an agent-based finite volume (“conveyor-belt”) method. We recently reported an application of this method in a study on oxygen distribution in retinal capillaries [3]. Each sinusoid segment consists of an array of “conveyor blocks” (**CB**s) that convey xenobiotic and blood downstream. The size of the CBs on each sinusoid segment is proportional to the local blood flow velocity. Assume that at time *t* the mass of xenobiotic within the *r*th CB on capillary segment *ij* is *M*_*r*_(*t*), then the characteristic equation describing advective movement of xenobiotic in the sinusoid segment within a time period of Δ*t* is:

$$\begin{array}{c} M_{r+1}\left(t+\Delta t\right)=M_{r}\left(t\right) \end{array}$$ (2)

**Passive transport process:** To simulate the passive transport process of the xenobiotic, we assumed that intra-object xenobiotic concentration within SIN or HEP instances is uniform everywhere within the object (the object is “well stirred”) and xenobiotic transfer only occurs at the interface between two adjacent objects. Passive (diffusive) transport is bi-directional between neighboring objects (*i*) and (*j*). The rate of passive transport is determined by the diffusive trans-membrane rate constant *D* (a permeability coefficient), interfacial area *S*_(*i*)(*j*)_, the center-to-center distance *d*_(*i*)(*j*)_ between the two objects, and the source objects volume *V*_(*i*)_. The change of xenobiotic concentration in object (*i*) with object volume *V*_(*i*)_ due to passive transport within a time period of Δ*t* is given by:

$$\begin{array}{c} c_{\left(i\right)}\left(t+\Delta t\right)-c_{\left(i\right)}\left(t\right)=\Delta t\cdot \sum_{\left(j\right)}^{neighbors}\frac{1}{V_{\left(i\right)}}\cdot \frac{D\cdot \left(c_{\left(j\right)}\left(t\right)-c_{\left(i\right)}\left(t\right)\right)\cdot S_{\left(i)(j\right)}}{d_{\left(i)(j\right)}} \end{array}$$ (3)

**Active transport process:** To simulate the active transport process of the xenobiotic, we use Michaelis-Menten kinetics. Transporter-mediated transport is uni-directional from SIN to HEP with no reverse flux (though a reverse flux may exist due to the passive transport model). The rate of active transport from SIN object *n* to HEP object *m* is determined by the maximum uptake rate $V_{max}^{AT}$ and half-saturating concentration $K_{m}^{AT}$. The change of xenobiotic concentration in HEP object (*m*) due to active transport within a time period of Δ*t* is given by:

$$\begin{array}{c} c_{\left(m\right)}\left(t+\Delta t\right)-c_{\left(m\right)}\left(t\right)=\Delta t\cdot \sum_{\left(n\right)}^{SIN\;neighbors}\frac{V_{\left(n\right)}}{V_{\left(m\right)}}\cdot \frac{V_{max}^{AT}\cdot c_{\left(n\right)}\left(t\right)}{K_{m}^{AT}+c_{\left(n\right)}\left(t\right)} \end{array}$$ (4)

To simplify the parameters in Eq 4 we normalize the half-saturating concentration $K_{m}^{AT}$ to the concentration of xenobiotic in the inflow by defining the unitless parameter ***α*** as the ratio of the half saturating concentration of active uptake to inflow concentration, $K_{m}^{AT}/c_{0}$. Therefore, *α* is the de-dimensionalized half-saturating concentration for uptake. In addition, we normalized the active transport $V_{max}^{AT}$ and $K_{m}^{AT}$ pair as ***β***, the ratio of maximum active uptake rate to half saturating concentration, $V_{max}^{AT}/K_{m}^{AT}$, giving a pseudo-first order rate constant with units of 1/*time*. *β* is the ratio of maximum uptake rate to *α*, which gives a first order rate constant. If *α* ≪ 1, uptake of xenobiotic via active transport is almost always saturated and therefore obeys zeroth-order kinetics. Conversely, if *α* ≫ 1, uptake is never saturated and follows first order kinetics.

**Metabolic process:** To simulate the metabolic processing of the xenobiotic, we used Michaelis-Menten kinetics. A single metabolic pathway, as a sink for the xenobiotic, reflects removal of compound from hepatocytes and the virtual lobule with maximum metabolic rate $V_{max}^{M}$ and half-saturating concentration of $K_{m}^{M}$. For simplicity we assumed that the metabolism is never saturated and assign $K_{m}^{M}$ a value that is much greater than inflow concentration of the blood-borne xenobiotic introduced at the PTs, which is the maximum value for *c*_(*k*)_. The change of xenobiotic concentration in HEP object (*k*) due to metabolism within a time period of Δ*t* is calculated:

$$\begin{array}{c} c_{\left(k\right)}\left(t+\Delta t\right)-c_{\left(k\right)}\left(t\right)=-\Delta t\cdot \frac{V_{max}^{M}\cdot c_{\left(k\right)}\left(t\right)}{K_{m}^{M}+c_{\left(k\right)}\left(t\right)} \end{array}$$ (5)

To simplify the parameters in Eq 5 we define a clearance factor *CL*_*int*_ as the ratio $V_{max}^{M}/K_{m}^{M}$, giving a pseudo-first order rate constant with units of 1/*time*.

**Coordination of processes:** The model needs to coordinate simulations of advective and passive/active transport processes properly to ensure conservation of xenobiotic mass *M*_r*_, since the former is modeled on a chain of CBs while the latter is modeled between SINs and HEPs. A SIN may contain multiple associated CBs as long as the center of a particular CB r* is enclosed within the volume of SIN *V*_(*s*)_. Before simulation of passive/active transport, xenobiotic masses on the CB array were converted into SIN objects. The conversion was calculated using:

$$\begin{array}{c} c_{\left(s\right)}\left(t\right)=\sum_{r^{*}}^{associated\;CBs}M_{r^{*}}\left(t\right)\cdot \frac{1}{V_{\left(s\right)}} \end{array}$$ (6)

Passive and active transport was then calculated between all pairs of objects that were in contact. The calculations were done in random order to avoid introducing order-dependent effects.

After simulation of passive/active transport, the xenobiotic mass within each SIN object was converted back to its associated CBs. To maintain mass balance, the conversion was done such that each associated CB had the same percentage change of xenobiotic mass due to passive/active transport as the SIN object did. Assuming an object has $N_{\left(s\right)}^{CB}$ associated CBs, this conversion process was calculated using:

$$\begin{array}{c} M_{r^{*}}\left(t+\Delta t\right)=M_{r^{*}}\left(t\right)\cdot \frac{c_{\left(s\right)}\left(t+\Delta t\right)}{c_{\left(s\right)}\left(t\right)} \end{array}$$ (7)

**Boundary conditions:** Nodes in the sinusoid network that overlap the CV or PTs are at a fixed blood pressure difference Δ*P*, which permits calculation of blood flow across the entire sinusoid network and blood pressure values at all sinusoid nodes. Nodes in the sinusoid network that overlap with the PTs act as the source of blood-borne xenobiotic introduced into the sinusoid network (and therefore the liver lobule) and nodes in the sinusoid network that overlap the CV act as sinks where any remaining blood borne xenobiotic exits the lobule.

### Study design

To address our two driving questions, is the complex lobular blood flow pattern sufficient to explain zonal metabolism and what characteristics of a xenobiotic make detailed lobule flow modeling a requirement to predict the pattern of zonal injury, we designed experiments to identify the parameter space of transport and metabolism domains that generate distinct exposure patterns within the simulated lobule. Specifically, are there parameter domains for which zonally different microdosimetry patterns are *emergent* properties of the xenobiotic’s characteristics and the complex blood flow within the lobule? In addition, to address the issue of when a complex ***NET*** lobule model is required, versus simpler ***PIPE*** or ***BOX*** models, we use parameter sets that lead to the *lack* of emergence of zonal differences in microdosimetry to define the xenobiotic parameter space where the simpler models are sufficient.

**Fig 4:**
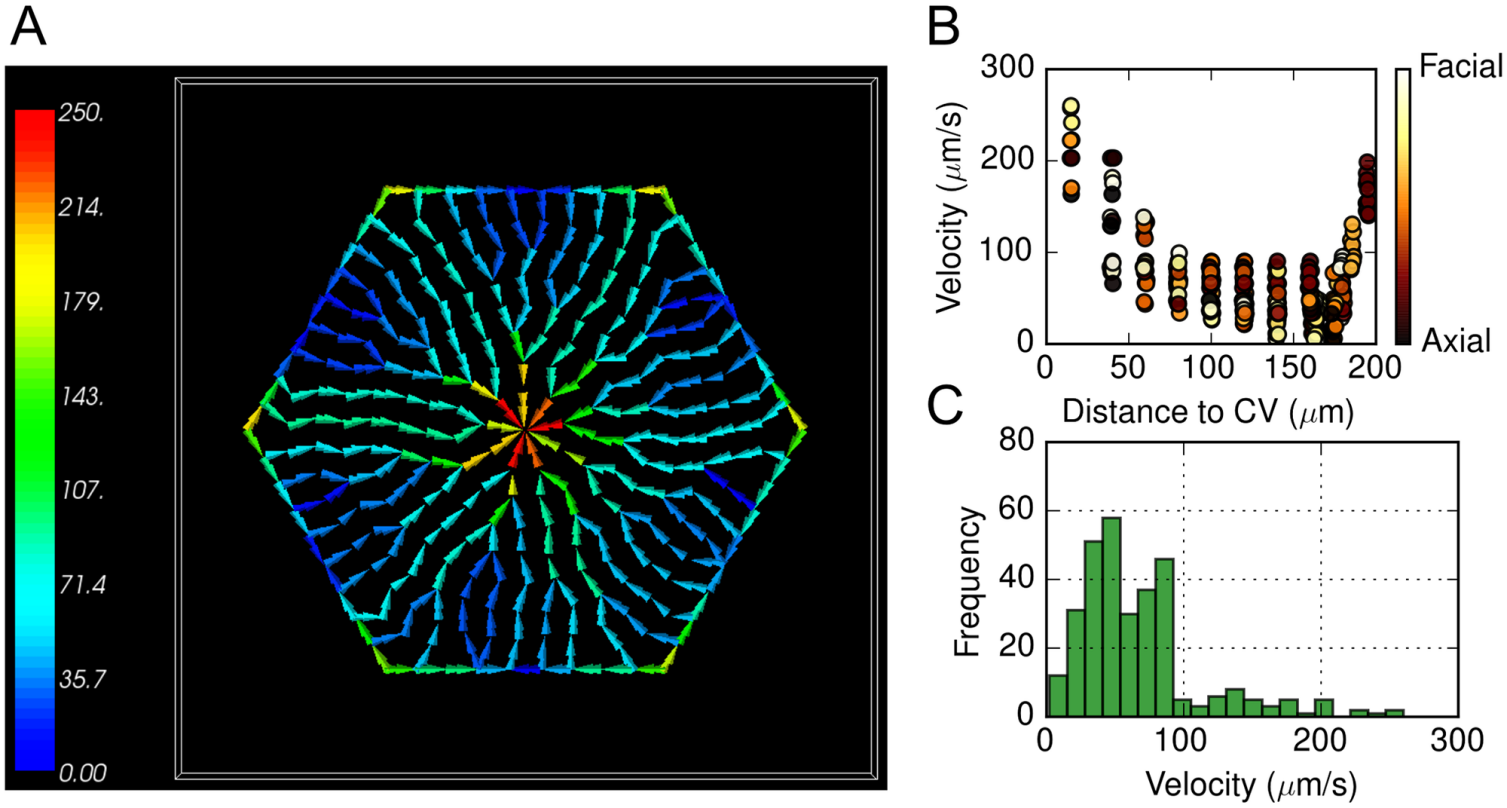
Spatial map and quantitative analysis of calculated flow velocities within the virtual sinusoid network. (A) Spatial map of flow velocities. Warmer color represents greater flow velocity. Color bar units are *μm*/*s*. (B) Calculated flow velocities in individual sinusoids segments with respect to their distances to central vein. Color codes angular positions with black indicating axial (vertex/PT to CV) and white facial (center of lobule face to CV) flows. (C) Histogram of calculated flow velocities.

Given our objectives and the number of parameters in the model, we designed two types of simulations: **PULSE** and **PERSISTENT**. **PULSE** simulations introduce a square pulse of xenobiotic with constant concentration into the lobule via the portal triads. The duration of the square pulse was 0.1 seconds, which is approximately the time it takes for a portion of blood to travel the distance of one hepatocyte width based on a sinusoid linear blood velocity of 200 *μm*/*s* (Fig 4). **PERSISTENT** simulations introduce a constant concentration of xenobiotic in the input blood flow throughout the simulation. In **PULSE** simulations, we simulated a time of 20 seconds, which is approximately 15 times the fastest blood transit times (estimated for velocities along six porto-central axial flow pathways shown in Fig 4) and is sufficient to develop gradients across the lobule. In **PERSISTENT** simulations, we used a longer simulated time of 5 minutes to allow distributions of xenobiotic to reach steady state.

In **PULSE** simulations, we measured the distribution of xenobiotic as a function of either diffusive (*D*) or transporter-mediated (*α*, *β*) transport parameters. The goal of **PULSE** simulation is to determine the individual contributions of passive and active transport to the microdosimetry pattern in the context of the complex blood flow. In **PERSISTENT** simulations, we explored passive and active transport mechanisms simultaneously to determine the interactions between the two transport processes and their combined contributions to the microdosimetry pattern in the context of the complex blood flow. Next, we added the metabolic flux (***C**L*_***i**nt*_, see Fig 3D) to each hepatocyte, which further alters the distribution of xenobiotic by irreversible trapping the xenobiotic within individual hepatocytes. Overall, we examined a range of *α*, *β* and *CL*_*int*_ values to define the xenobiotic parameter space that gives rise to zonally different microdosimetry patterns as outlined in Table 2.

**Table 2:** Xenobiotic dependent simulation parameters for transport and metabolism.

Parameter	Description	Value or Range
*D*	Diffusive (passive) trans-membrane rate constant for xenobiotic transfer	10^−7^–10^−5^ *cm*^2^/*sec*
*α*	$K_{m}^{AT}/c_{0}$, ratio of half saturating concentration of active uptake to inflow concentration	0.01–10
*β*	$V_{max}^{AT}/K_{m}^{AT}$, ratio of maximum active uptake rate to half saturating concentration	0.1–10/*sec*
$K_{m}^{M}$	Half saturating concentration of xenobiotic metabolism	10 *mmol*/*L*
*CL*_*int*_	$V_{max}^{M}/K_{m}^{M}$, ratio of maximum metabolic rate to half saturating concentration	0.01–10/*sec*

## Results

### Blood flow in the simulated lobule

Fig 4 shows the calculated blood flow velocities within a representative virtual mouse liver lobule, additional examples are given in Section 2.2 in S1 Text. To our knowledge, no experimental values for Δ*P* in mouse liver lobule are available. Reported values in other species include Δ*P* ≈ 3 *mmHg* in rat liver lobules [40–43] and <1 *mmHg* in rabbit liver lobules [44]. Macphee *et al*. reported [45] average flow velocity (≈ 70 *μm*/*s*) within the sinusoid network of mouse liver lobules, which was significantly smaller than that in rat liver lobules (250–430 *μm*/*s*) [45]. To account for this apparent inter-species difference of flow velocity, we assumed a smaller PT–to–CV pressure drop for the virtual mouse lobule of Δ*P* = 0.6 *mmHg* (Table 2). The calculated average blood flow velocity with this Δ*P* is $\bar{v}=67.5\;\mu m/s$ in the virtual liver lobule, similar to the value reported by Macphee *et al* [45]. The calculated total volumetric blood flow rate is $\dot{Q}=97.7\;pL/s$. By scaling this calculated flow rate of the simulated lobule volume of 2 × 10^6^ *μm*^3^ to the whole mouse liver volume of about 1.3 *cm*^3^ gives a total liver blood flow rate of ${\dot{Q}}_{liver}^{calc}=3.9\;mL/min$, which is similar to the value reported by Davies *et al*. of 3.3 *mL*/*min* [46]. In addition, the blood flow velocities calculated in CC3D were compared to results obtained using the capillary blood flow calculator *NetFlow* [38, 47] (Section 2.1 in S1 Text).

pericentral (a.k.a. peri-venous, centri-lobular) sinusoid segments have greater blood flow velocities (up to 250μm/s) than peripheral sinusoid segments do (which are as low as 10μm/s), consistent with the calculations of others [19, 48]. Azimuthally, calculated flow velocities within the virtual liver lobule are generally greater along the six PT–to–CV axes compared to the perimeter-face–center to CV, showing an angular discrepancy (Fig 4B). This angular discrepancy suggests that blood flow streams have path-dependent transit times (*i.e*., time interval between entering the lobule at a portal triad and exiting via the central vein) across the simulated liver lobule. Using the flow map, the estimated transit times of blood varies from about 1.5 seconds to more than 5 seconds. Blood flow along the PT–to–CV axes has smaller transit times than along the longer and more complex pathway from a PT, along a perimeter face of the bounding hexagon and then to the CV.

We will use the terms “**axial**” an “**facial**” in the remainder of this text to describe 30° azimuthal regions containing these two types of flow pathways, respectively. Such heterogeneous blood flow distribution, where flow pathways from the PTs to the CV exhibit varying transit times, contribute to variation in exposure of hepatocytes to the xenobiotic within the lobule (as discussed below).

We also explored the effects of different sinusoid anastomotic patterns (Section 2.2 in S1 Text), different lobule sizes (Section 2.3 in S1 Text), and different PT–to–CV pressure drops (Section 2.4 in S1 Text) on calculated blood flow distribution in the virtual mouse liver lobules. In studying different anastomotic patterns we observed consistent results among different virtual lobules in terms of mean hepatocyte volume ${\bar{V}}_{hep}$, sinusoids volume fraction *η*_*sin*_, average sinusoid-parenchymal interfacial area per unit parenchymal volume *S*_*int*_, mean blood flow velocity $\bar{v}$ and total lobular volume inflow rate $\dot{Q}$ (Section 2.2 in S1 Text). In addition, we examined the effects of lobule diameter on the hemodynamic features of virtual liver lobules, we observed similar spatial pattern of blood flow velocities in a virtual liver lobule with a 2x larger lobule diameter (400 *μm*) where direct flow paths from PT to CV typically had greater velocity than indirect flow pathways and flow paths near PT’s or the CV typically showed greater flow velocity than mid-zonal paths (Section 2.3 in S1 Text). The larger lobule had similar distributions of flow velocities but with a smaller fraction of flow paths with blood flow velocity greater than 100 *μm*/*s*. Mean blood flow velocity within the larger lobule was $\bar{v}=36.7\;\mu m/s$, nearly 50% smaller than that within the smaller default lobule.

Since the processing orders of cells in calculating transport and metabolism is stochastic, we selected three parameter sets to examine run-to-run variability in the model outputs lobular-wise mean xenobiotic concentration and central-to-peripheral ratio at 20 seconds (see Section 2.10 in S1 Text). We found that the variability in outputs was small and therefore multiple runs for a particular parameter set were not needed.

By studying the effects of the PT–to–CV pressure drop on the hemodynamic features of the virtual liver lobules, we found that mean blood flow velocities increase proportionally with increasing pressure drop (Section 2.4 in S1 Text). A PT–to–CV pressure drop of Δ*P* = 3 *mmHg* (reported value for rat liver) resulted in mean flow velocity of $\bar{v}=338\;\mu m/s$, in agreement with reported values in [40–43, 45].

### Hepatic exposure to PULSE xenobiotic

To explore the simplest case of xenobiotic uptake across the simulated lobule we examined the effect of varying the passive transport rate constant, in the absence of active transport and metabolism, for a narrow pulse (Δ*t* = 0.1*s*) of xenobiotic. In passive transport-only **PULSE** simulations, xenobiotic was absorbed into the hepatocytes and eventually cleared from the cells back to the blood (see Section 2.5 in S1 Text for ***PIPE*** and Section 2.6 in S1 Text for ***NET***). It should be noted that in a particular simulation the same diffusive trans-membrane rate constant was applied to both cell-blood and cell-cell transfers, but this did not guarantee the same diffusive flux, which depends also on variables such as interface area and blood flow velocity. We observed two xenobiotic distributions in the **PULSE** simulations using the ***PIPE*** model; uniform across the simulated pipe for low uptake rates and preferentially periportal at high uptake rates followed by a uniform and then pericentral high time periods as the xenobiotic washed out of the system after the end of the input pulse.

We next studied the effect of active transport in **PULSE** simulations. In these simulations, cell-to-cell and blood-to-cell diffusive flux is turned off. Without an efflux pathway for the xenobiotic it remains within a given cell once it is actively imported. We are interested in determining if certain regions of the lobule accumulate more xenobiotic and if this accumulation is specific to the ***NET*** model. Interestingly, although hepatocytes along the six porto-central axes in the ***NET*** model experience pulses of xenobiotic sooner, they generally failed to absorb more. Also strikingly and somewhat counter-intuitively, pericentral hepatocytes, on average, accumulated more xenobiotic than peripheral hepatocytes. Both phenomena can be interpreted as a consequence of lobule-wide small uptake rate but different cumulative uptake *durations*. In PULSE simulations where the pulse size is as narrow as ΔT = 0.1s, pericentral hepatocytes have the advantage of encountering a greater number of derived pulses split from the original incoming pulse due to variable transit times available in the network structure. In contrast, periportal cells experience only a single, short duration pulse. As a result, pericentral cells tended to accumulate more xenobiotic. We believe that though this higher pericentral pattern is interesting it may have relatively little biological relevance. In order to observe this behavior in the ***NET*** model requires an input pulse width that is less than the average transit time of blood through the lobule, that is, less than 1 second (see Section 2.7 in S1 Text). It seems unlikely that a liver lobule would ever encounter a transient concentration for a time period this brief.

### Hepatic exposure to PERSISTENT xenobiotic

We next moved to simulate **PERSISTENT** input flow of xenobiotic, where the concentration of incoming xenobiotic was maintained at *c*_0_ = 1 *mmol*/*L* throughout a simulated 5-minutes. Compared with **PULSE** simulations, **PERSISTENT** simulations represent physiologically realistic xenobiotic exposures. In active transport-only simulations, the central-to-peripheral ratios were similar to the 0.1-second **PULSE** simulation (see Section 2.9 in S1 Text) except the higher pericentral accumulation of xenobiotic at small-*α* values was not observed. For large-*α* and large-*β* values, the xenobiotic was preferentially taken up in periportal regions, a consistent phenomenon in both **PERSISTENT** and **PULSE** simulations (Section 2.8 in S1 Text).

**Fig 5:**
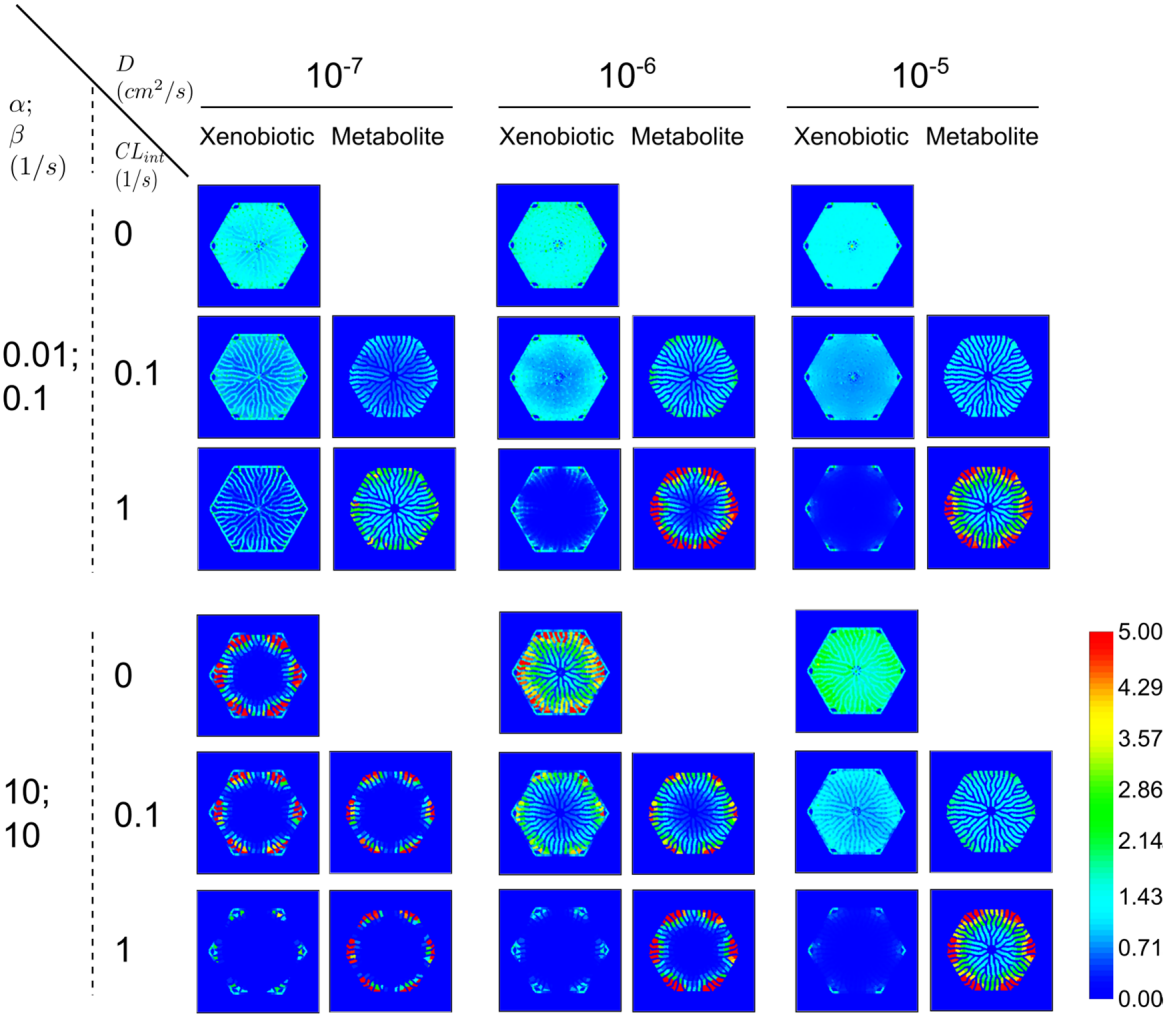
Transient xenobiotic and metabolite concentrations at *t* = 20 *s* in select PERSISTENT simulations. (*α*, *β*) pairs are (0.01, 0.1/*s*) and (10, 10/*s*) in upper and lower panels, respectively. In each panel, *CL*_*int*_, from top to bottom row, are 0/*s*, 0.1/*s*, and 1/*s*, respectively. *D*s, from left to right column, are 10^−7^, 10^−6^, and 10^−5^ *cm*^2^/*s*, respectively. Color scale has units *mmol*/*L*. See Section 3 in S1 Text and S1 Animations for animations of these simulations.

While some xenobiotics may only have one uptake mechanism (either passive or active transport) more generally, xenobiotics will have both mechanisms with different relative rates determined by the biochemical and biophysical properties of the particular xenobiotic. Therefore, we investigated the xenobiotic distribution in **PERSISTENT** simulations across a range of passive and active transport rates. As before, we assumed that the diffusive trans-membrane rate constant of xenobiotic movement between neighboring hepatocytes was identical to that between blood and hepatocytes. Snapshots of lobular xenobiotic distribution at *t* = 20*s* are shown for two extremes of active transport parameters and three selected diffusive trans-membrane rate constants in Fig 5. Initially we will focus on the no metabolism (*CL*_*int*_ = 0/*s*) rows, and we will discuss situations with metabolic flux later.

For extremely small (*α*, *β*) pairs, variation of the diffusive trans-membrane rate constants made little difference in the azimuthal distribution but did affect the radial distribution. For large diffusive trans-membrane rate constant the xenobiotic rapidly distributes between neighboring hepatocytes and between hepatocytes and blood. For extremely large (*α*, *β*) pairs, the diffusive trans-membrane rate constants significantly influenced the xenobiotic distribution. A small diffusive trans-membrane rate constant resulted in preferential accumulation of xenobiotic at the periportal region. In contrast, a large diffusive trans-membrane rate constant resulted in uniform xenobiotic distribution across the entire lobule. An intermediate diffusive trans-membrane rate constant resulted in intermediate radial and azimuthal differences in distribution. These comparisons illustrate quite different scenarios; when active transport was dominant, when passive transport was dominant, and when active and passive transport were similar. Note that the relative rate of active and passive transport for a particular xenobiotic will depend on the number of transporters, the biochemical and biophysical properties of the xenobiotic as well as the local xenobiotic concentration. As will be examined below, incorporation of metabolism also affects the spatial distribution of the xenobiotic.

Xenobiotics absorbed into hepatocytes often undergo metabolism. The model incorporates a single metabolic pathway as a route of clearance for the xenobiotic to examine how xenobiotic removal (metabolism) alters the net hepatic exposure and microdosimetry. The metabolic process irreversibly transforms the xenobiotic into a metabolite with Michaelis-Menten kinetics. The intrinsic clearance *CL*_*int*_ characterizes the metabolic process, and defines the ratio of the maximum reaction rate $V_{max}^{M}$ to the half-saturating concentration $K_{m}^{M}$ for metabolism. As our aim is to explore possible spatial variation in hepatic exposure to the parent xenobiotic, the model has no transport process for the metabolite and therefore the metabolite accumulates in the hepatocytes. The model also does not impose any zonation of the metabolic process in the virtual liver lobule. Therefore, any spatial variation in the hepatic exposure (or accumulation of metabolite) would be *emergent from the spatiality of the virtual liver lobule*. Fig 5 shows the spatial patterns of the xenobiotic and metabolite in **PERSISTENT** simulations with *CL*_*int*_ = 0.1/*s* and *CL*_*int*_ = 1/*s*.

With weak active transport (*α* = 0.01, *β* = 0.1/*s*) and weak metabolism (*CL*_*int*_ = 0.1/*s*), stronger passive transport resulted in a more uniform distribution of both xenobiotic and metabolite compared to weaker passive transport. This indicates that *passive transport functions to diminish the spatial variability through cell-to-cell transfer of xenobiotic*. The situation is very different if active transport is weak but metabolism is increased (*CL*_*int*_ = 1/*s*). In this case, with a small diffusive trans-membrane rate constant, the distribution of xenobiotic was even and the metabolite was distributed uniformly radially and azimuthally. In addition, the amount of xenobiotic remaining in the blood was significant. This is the result of lobular-wide slow uptake coupled with fast metabolism. With intermediate diffusive trans-membrane rate constants, the blood concentration of xenobiotic was very low in most of the lobule, and accumulation of metabolite was primarily seen in the lobule’s periphery. The azimuthal gradient in metabolite distribution was not as obvious as the radial gradient. This pattern shows the competition between the two transport fluxes and the single metabolism flux. Passive transport aided uptake of the xenobiotic from the blood, followed by either metabolism or diffusion into neighboring hepatocytes. It appears that the metabolizing flux and cell-to-cell diffusive flux had comparable contributions in this case, because a large area of high metabolite concentration was observed as well as some finger-like protrusive patterns of moderate metabolite levels. With a large diffusive trans-membrane rate constant, the region of high metabolite concentration expanded somewhat, giving a more uniform distribution of metabolite into the mid-zonal and centri-lobular regions. The lobule acquired a periportal pattern of preferential azimuthal metabolite gradients as well. As will be discussed later, such a spatial pattern is characterized by a small central-to-peripheral ratio and large axial-to-facial ratio. Interestingly, variation of the diffusive trans-membrane rate constant alone, with fixed weak active transport (*α* = 0.01, *β* = 0.1/*s*) and strong metabolism (*CL*_*int*_ = 1/*s*), resulted in a non-linear and non-monotonous change in regional accumulation of metabolite, especially for centri-lobular hepatocytes.

With strong active transport (*α* = 10, *β* = 10/*s*), similar patterns were observed for large diffusive trans-membrane rate constant, little spatial difference in distribution of metabolite were seen at low metabolism levels. This suggests that passive transport is the dominant driver for the observed spatial pattern, and active transport plays only a minor role in patterning. However, active transport did make a difference in the blood level of xenobiotic as higher active transport rates left little xenobiotic in the sinusoidal space. In contrast, with a small diffusive trans-membrane rate constant, active transport was dominant and controlled the distribution of both the xenobiotic and metabolite, regardless of metabolic rate. With strong active transport, xenobiotic accumulated in the lobular periphery, in particular at periportal regions. Intermediate diffusive trans-membrane rate constants led to a pattern reminiscent of both cases above, with intermediate radial and azimuthal gradient of xenobiotic and metabolite. Therefore, if active transport is strong, the variation of diffusive rate constant alone, regardless of metabolic rate, results in a monotonous change in radial accumulation of xenobiotic and metabolite. This is strikingly different from the case for weak active transport discussed earlier.

**Fig 6:**
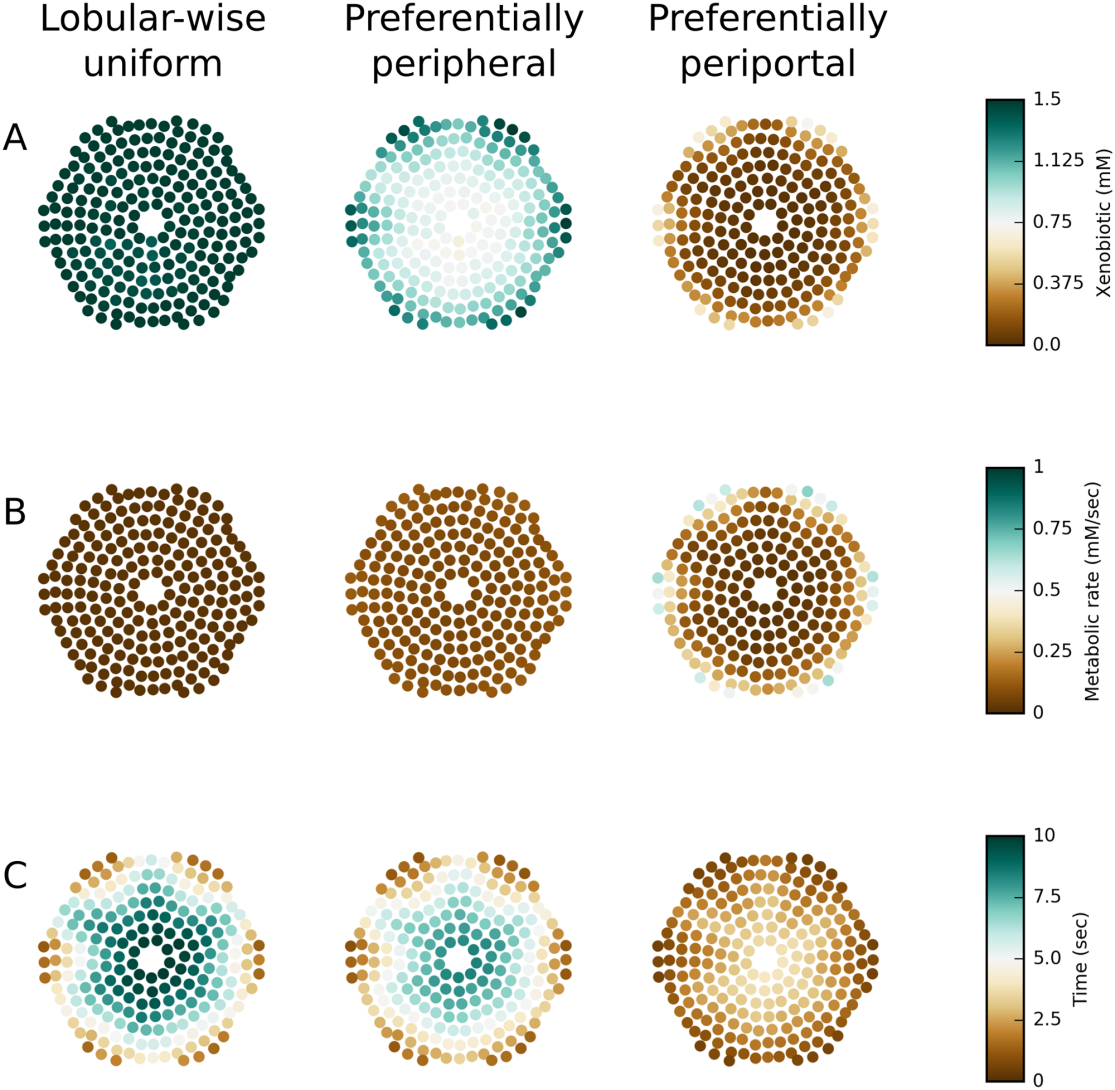
Spatial maps of select steady-state quantities within the virtual liver lobule. (A) xenobiotic concentration; (B) metabolic rate; (C) time for the xenobiotic concentration to reach half of steady-state value. Solid circles indicate the positions of individual hepatocytes in the virtual liver lobule slice. These three simulations have the same transport parameters (*α* = 1, *β* = 1/*s* and *D* = 1 × 10^−6^ *cm*^2^/*s*) and only differ in metabolism parameters (from left to middle to right column, *CL*_*int*_ = 0.01/*s*, 0.1/*s*, 1/*s*, respectively).

**Fig 7:**
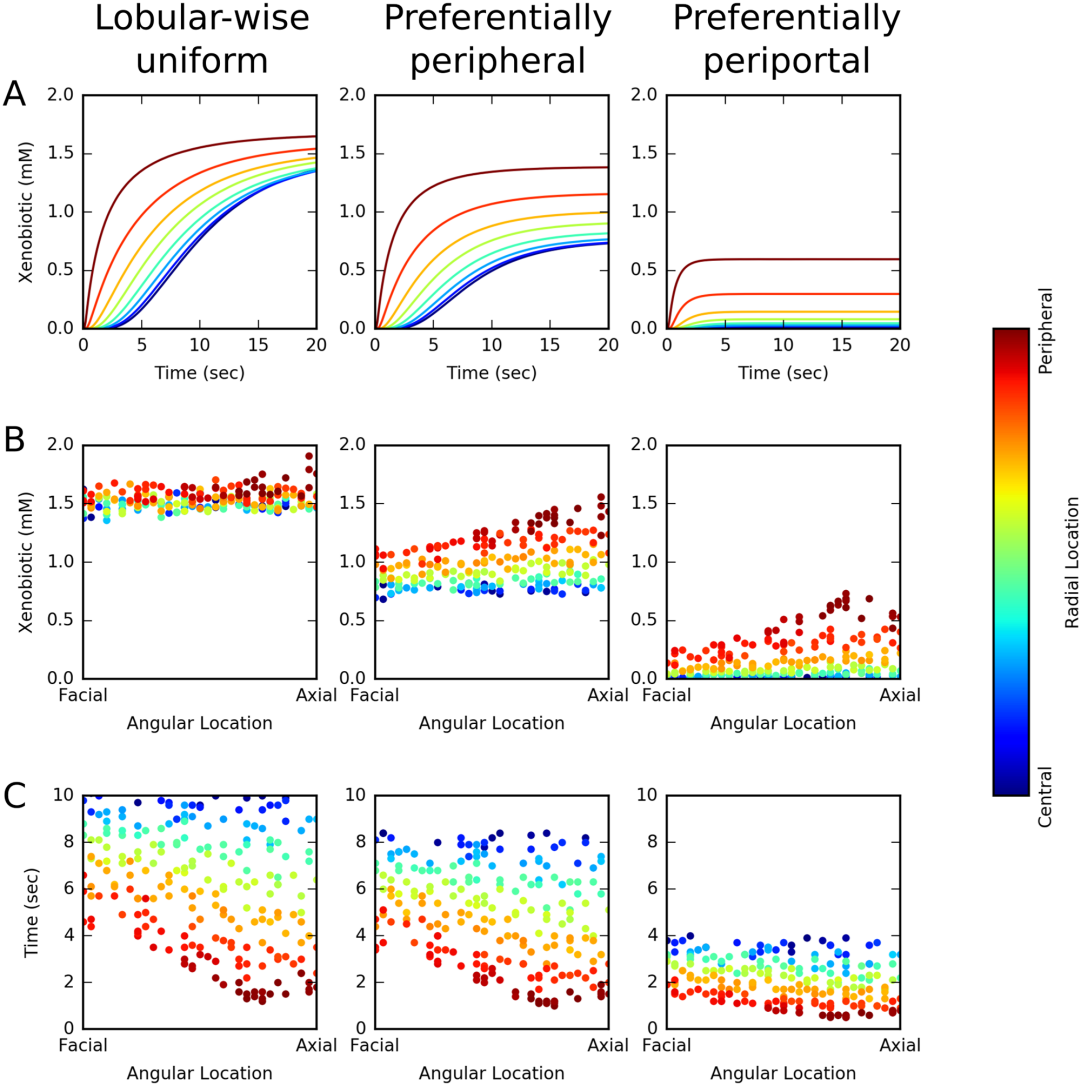
Spatial and temporal characteristics of xenobiotic distribution within the virtual liver lobule. (A) Time course of radially zonal average xenobiotic concentration in the first 20 seconds of simulation; (B) Steady-state xenobiotic concentrations in individual hepatocytes; (C) time for the xenobiotic concentration to reach half of steady-state value in individual hepatocytes. Color codes radial location of cells/zones from central to peripheral. These three simulations have the same transport parameters (*α* = 1, *β* = 1/*s* and *D* = 1 × 10^−6^ *cm*^2^/*s*) and only differ in metabolism parameters (from left to middle to right column, *CL*_*int*_ = 0.01/*s*, 0.1/*s*, 1/*s*, respectively).

**PERSISTENT** simulations with various combinations of transport and metabolism parameters show one of three *steady-state* patterns of hepatic exposure to the xenobiotic: *lobular-wise uniform*, *preferentially peripheral* (radially varying), and *preferentially periportal* (both radially and azimuthally varying). Figs 6 and 7 illustrate spatial and temporal features of three representative simulations that emergently produce these patterns of hepatic exposure to the xenobiotic. These three simulations have the same active and passive transport parameters, but differ in the rate of metabolism with *CL*_*int*_ = 0.01/*s*, *CL*_*int*_ = 0.1/*s* and *CL*_*int*_ = 1/*s*, respectively.

In *lobular-wise uniform* hepatic exposure, the xenobiotic distributes uniformly across the entire virtual lobule at steady state (left column in Figs 6 and 7). In other words, the radial and azimuthal variations in the xenobiotic are minor (Fig 7B). This hepatic exposure pattern resulted from the interaction between relatively weak metabolism and moderate transport that leads to initial “trapping” of the xenobiotic near the PTs with gradual diffusive “spreading” over time (left column in Figs 6C and 7C). As a result, despite insignificant steady state regional variations in hepatic exposure to the xenobiotic, this simulation demonstrated great variation in the time required by individual hepatocytes to reach half of the steady state xenobiotic concentration. Not unexpectedly, periportal cells established their steady states faster than mid-zonal and pericentral cells (Figs 6C and 7C). To summarize, periportal hepatocytes took up and metabolized the xenobiotic early due to their positional advantage, but if the lobular-wise metabolism was relatively weak these cells could spread xenobiotic to mid-zonal and pericentral cells over time, eventually leading to a steady-state *lobular-wise uniform hepatic exposure to the xenobiotic*.

In **preferentially peripheral** hepatic exposure, the xenobiotic distributed primarily in the periphery of the lobule with little or no azimuthal gradient at steady state (middle column in Figs 6 and 7). periportal (i.e., peripheral axial) cells, and to a slightly less extent peripheral facial cells, displayed greater exposure to the xenobiotic than pericentral cells (Fig 6A). For a given angular location, the central-to-peripheral difference in hepatic exposure to the xenobiotic was commonly larger than a factor of 2 (vertical dispersion of dots in the middle panel of Fig 7B). In contrast, for a given radial location, the axial-to-facial discrepancy in hepatic exposure to the xenobiotic was generally within of 25% (dots of a particular color in the middle panel of Fig 7B). This exposure pattern arose from the interaction between moderate transport and moderate metabolism that resulted in a competition between “trapping” and “spreading”. Compared with the first case when metabolism was weak, “trapping” was stronger keeping more xenobiotic in cells for metabolism, while “spreading” was less effective in distributing the xenobiotic between adjacent cells. Such interaction eventually led to a shallow gradient along PT–to–PT path (i.e., axial to facial to axial) and a steep gradient along PT–to–CV axis (i.e., peripheral to central). Concomitant with the emergent regional variation in steady state hepatic exposure to the xenobiotic, the time to reach half of steady state concentration displayed a regional variation that was similar to the pattern seen in the previous case where hepatic exposure to the xenobiotic was lobular-wise uniform (Figs 6C and 7C). Compared with the first case when metabolism was weak, regional variation of the time to steady state was smaller and lobular-wise average time was also smaller. Therefore, a faster establishment of steady state was observed when metabolism was faster. To sum up, with moderate metabolism competing with moderate passive transport, hepatocytes had limited ability to distribute the xenobiotic to adjacent cells, and therefore peripheral cells exhibit greater exposure to the xenobiotic than mid-zonal and pericentral cells did.

In **preferentially periportal** hepatic exposure, the xenobiotic predominantly appeared in the periportal hepatocytes, causing both steep radial and azimuthal gradients at steady state (right column in Figs 6 and 7). Due to strong metabolism, lobular average steady state concentration was significantly smaller than in the previous two cases and most of the xenobiotic was present in periportal cells (Fig 6A). Both radial (vertical dispersion of dots) and azimuthal (slope of trend of dots with same color) discrepancies in hepatic exposure to the xenobiotic were significant (Fig 7B). This exposure pattern arose from the effective xenobiotic “trapping” in the few periportal hepatocytes surrounding PTs, which was dominant over xenobiotic “spreading”. Not unexpectedly, in this case, time to arrive at half of steady state xenobiotic concentration was the smallest with small regional variation (Figs 6C and 7C). To sum up, strong metabolism outweighed moderate passive transport, periportal hepatocytes metabolized the majority of their absorbed xenobiotic and allowed minimal xenobiotic spreading to adjacent cells.

Only in trivial cases did we observe a predominantly pericentral pattern of hepatic exposure. In the trivial case of a **PULSE** exposure in the absence of metabolism a transient higher pericentral pattern is observed during the washout phase after the end of the input pulse in ***PIPE*** models and transiently, for some parameter sets, during the pulse phase in ***NET*** models.

To characterize the radial and azimuthal distributions of xenobiotic we define two descriptors. The descriptor for radial distribution, termed the central-to-peripheral ratio *γ*_*cp*_, is the ratio of the average xenobiotic concentration at the pericentral-most radial zone to that at the peripheral-most radial zone (Fig 17A in S1 Text). Scenarios with *γ*_*cp*_ > 1.25, 0.75 < *γ*_*cp*_ ≤ 1.25 and *γ*_*cp*_ ≤ 0.75 reflect preferentially pericentral, radially uniform and preferentially peripheral exposure to the xenobiotic, respectively. The descriptor for azimuthal distribution, termed the axial-to-facial ratio *γ*_*af*_, is the ratio of the average xenobiotic concentration at the six axial azimuthal zones to that at the six facial azimuthal zones (Fig 17B in S1 Text). Scenarios with *γ*_*af*_ > 1.25, 0.75 < *γ*_*af*_ ≤ 1.25 and *γ*_*af*_ ≤ 0.75 reflect preferentially axial, azimuthally uniform and preferentially facial exposure to the xenobiotic, respectively. (See also Section 2.6 in S1 Text)

The previous section introduced three characteristic hepatic exposure patterns based on the spatial and temporal features of xenobiotic distribution in three representative simulations. Here we present outcomes of all 300 **PERSISTENT** simulations in terms of their steady-state exposure patterns and discusses *possible combinations of transport and metabolism parameters that give rise to the three characteristic exposure patterns*.

We characterize the spatial exposure pattern of the xenobiotic using the two descriptors; central-to-peripheral ratio *γ*_*cp*_ and axial-to-facial ratio *γ*_*af*_ at steady state for radial and azimuthal variations, shown inFigs 8 and 9, respectively. The “heat heap map” is composed of 25 smaller *constituent heat map*s. In a *constituent heat map*, the horizontal axis reflects variation in *α* and the vertical axis variation in *β*. The top left corner of each *constituent heat map* corresponds to the lowest of active transport, and bottom right corner corresponded to the highest active transport. The diffusive trans-membrane rate constant *D* increases from the leftmost column to right. The intrinsic clearance *CL*_*int*_ (metabolism) increases from top (zero metabolism) to bottom row.

**Fig 8:**
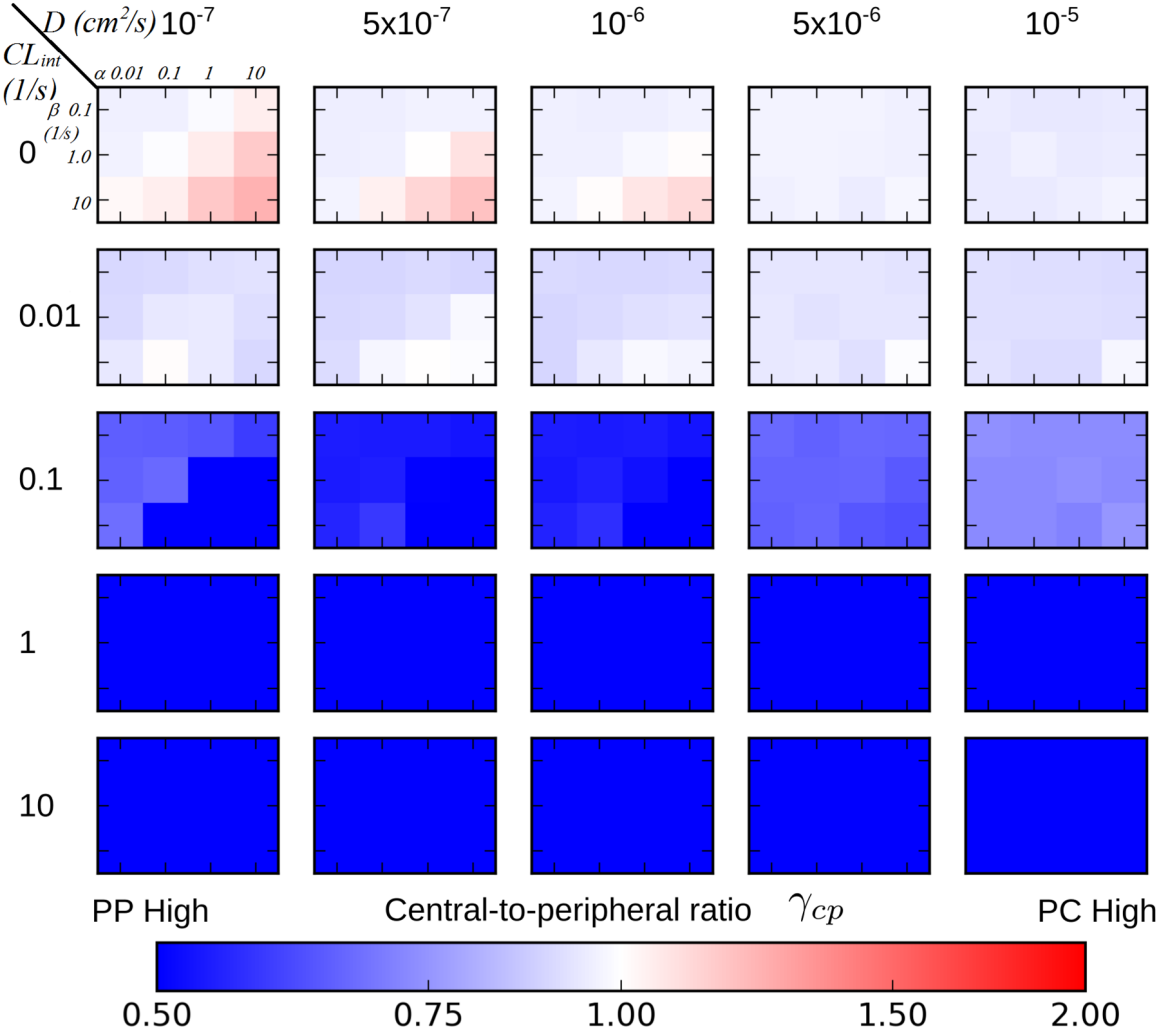
Heat maps of central-to-peripheral ratios at steady state in PERSISTENT simulations with various strengths of transport and metabolism. The complete heat map consists of 25 smaller heat maps, termed *constituent heat maps*. A *constituent heat map* contains 12 different (*α*, *β*) pairs (indicated on the upper-left most heat map), each of which represents varying strength of active transport. Diffusive trans-membrane rate constants, increase from left to right increasing passive transport. Intrinsic clearance *CL*_*int*_, increases from top to bottom which represents increasing metabolism. Warmer color reflects greater central-to-peripheral ratio, “PP” and “PC” refer to periportal and pericentral, respectively.

**Fig 9:**
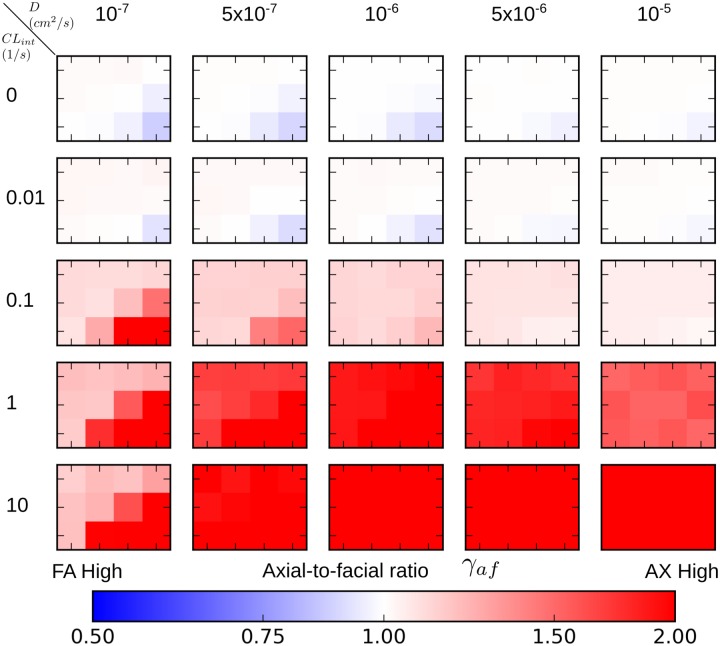
Heat maps of axial-to-facial ratios at steady state in PERSISTENT simulations with various strengths of transport and metabolism. The complete heat map consists of 25 smaller heat maps, termed *constituent heat maps*. A *constituent heat map* contains 12 different (*α*, *β*) pairs, each of which represents varying strength of active transport (refer to Fig 8 for details on the range of (*α*, *β*) pairs). Diffusive trans-membrane rate constants, increase from left to right, which represents increasing passive transport. Intrinsic clearance *CL*_*int*_, increase from top to bottom, which represents increasing metabolism. Warmer color reflects greater axial-to-facial ratio, “FA” and “AX” refer to facial and axial, respectively.

**Fig 10:**
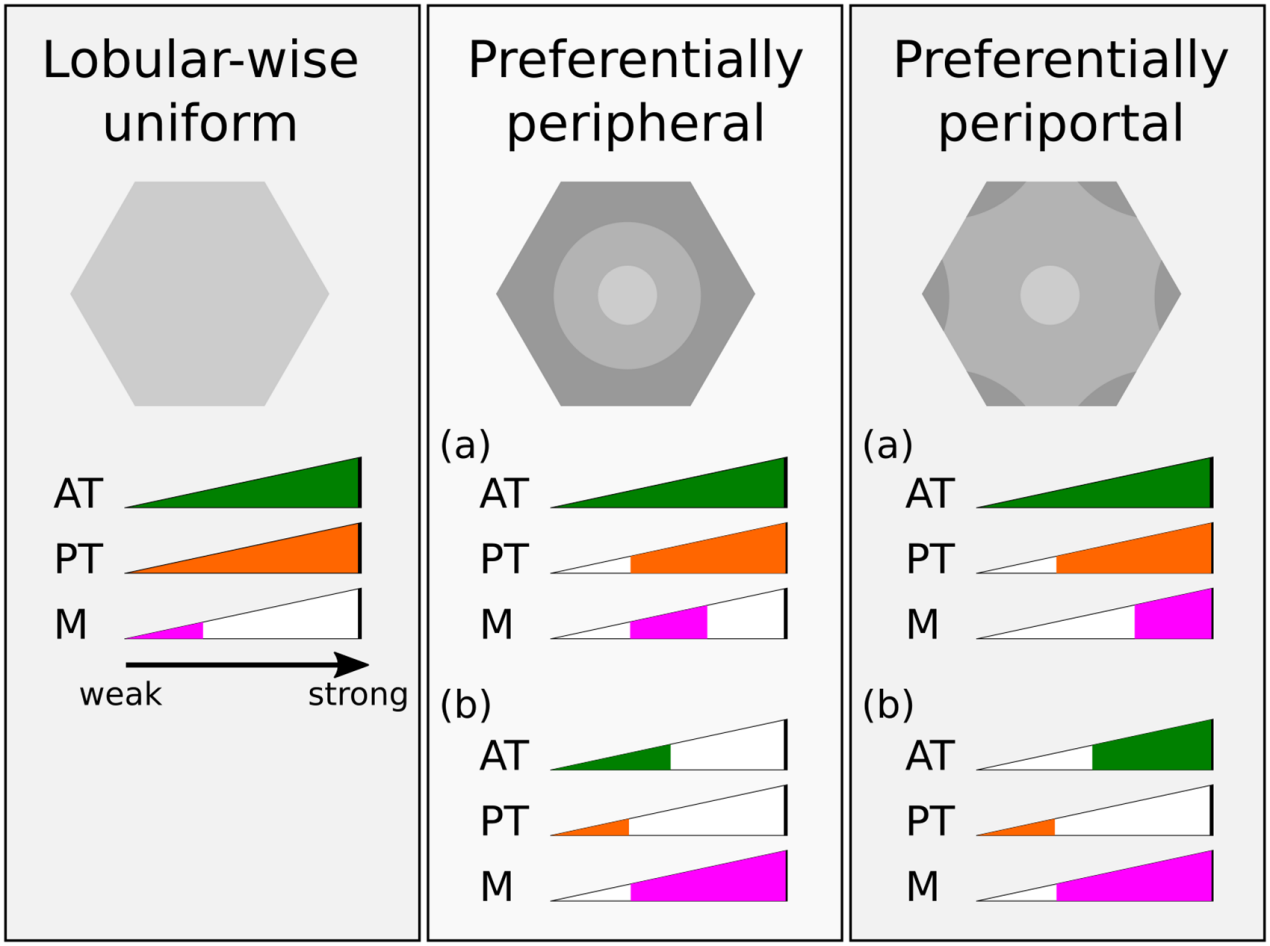
Schematics of the three emergent patterns of hepatic exposure at steady state in PERSISTENT simulations and corresponding parameter domains of transport and metabolism. Schematics of exposure patterns are shown in gray scale, qualitatively indicating high or low steady state xenobiotic concentrations. Parameter domains, colored region in “AT” (active transport), “PT” (passive transport) and “M” (metabolism) strength bars, define the range of transport and metabolism parameters that give rise to the corresponding spatial exposure patterns.

As discussed in the previous section, there are three characteristic exposure patterns of the xenobiotic: (1) *lobular-wise uniform*; (2) *preferentially peripheral*; (3) *preferentially periportal*. We discuss each of them below and will summarize the parameter domains that give rise to these patterns in Fig 10.

A *lobular-wise uniform* exposure pattern occurs in the transport and metabolism parameter domains where both central-to-peripheral and axial-to-facial ratios are close to 1 (0.75 < *γ*_*cp*_ ≤ 1.25, 0.75 < *γ*_*af*_ ≤ 1.25). According to the heat maps for radial and azimuthal variations, low metabolism generally resulted in a *lobular-wise uniform* exposure to the xenobiotic, regardless of strengths of passive and active transport (top two rows in Figs 8 and 9). This exposure pattern emerged primarily due to lack of, or ineffective, “trapping” of the xenobiotic by intracellular metabolism. As a consequence, cell-cell “spreading” via passive transport reduced the regional variation.

A *preferentially peripheral* exposure pattern occurs in domains where central-to-peripheral ratio is smaller than 1 and axial-to-facial ratio is close to 1 (*γ*_*cp*_ ≤ 0.75, 0.75 < *γ*_*af*_ ≤ 1.25). Parameter domains with moderate to strong passive transport (*D* = 5 × 10^−7^ *cm*^2^/*s* and larger) and moderate metabolism (*CL*_*int*_ = 0.1/*s*) satisfied this criterion (third row in Figs 8 and 9). This parameter domain reflected a competition between moderate “spreading” and moderate “trapping” that resulted in loss of the initially established azimuthal gradient but not the radial gradient. For certain active transport and metabolism parameter sets, the stronger the passive transport, the lower the radial variation. Another parameter domain with weak passive transport (*D* = 1 × 10^−7^ *cm*^2^/*s*) and comparably weak active transport (top left corner of the *component heat map*) and moderate to strong metabolism *CL*_*int*_ = 0.1, 1, 10/*s* also gave the preferentially peripheral exposure pattern. In this case, both active and passive transports were weak, so the xenobiotic absorption was lobular-wise small and cell-cell “spreading” was insignificant. Lobular-wise small extraction guaranteed small difference in concentration of blood-borne xenobiotic in peripheral and facial regions compared with periportal regions due a short travel distance of blood in between, but a still significant difference between peripheral and pericentral regions. Thus, a radial gradient, but not an azimuthal gradient, emerged. In addition, ineffective cell-cell “spreading” failed to diminish any initial radial variation in exposure.

A *preferentially periportal* exposure pattern corresponds to the domains where central-to-peripheral ratio is smaller than 1 and axial-to-facial ratio is larger than 1 (*γ*_*cp*_ < 0.75, *γ*_*af*_ > 1.25). All of the remaining domains in the heat map satisfied this condition. Briefly, most domains with strong metabolism (*CL*_*int*_ = 1, 10/*s*) showed radial and azimuthal variations in hepatic exposure to the xenobiotic. As an exception, scenarios when active transport was weak showed preferentially peripheral exposure with very mild azimuthal variation, as explained above. A small domain with moderate metabolism (*CL*_*int*_ = 0.1/*s*) but weak passive transport also showed preferentially periportal exposure. The three observed exposure patterns and the causative parameter domains are summarized in Fig 10.

**Fig 11:**
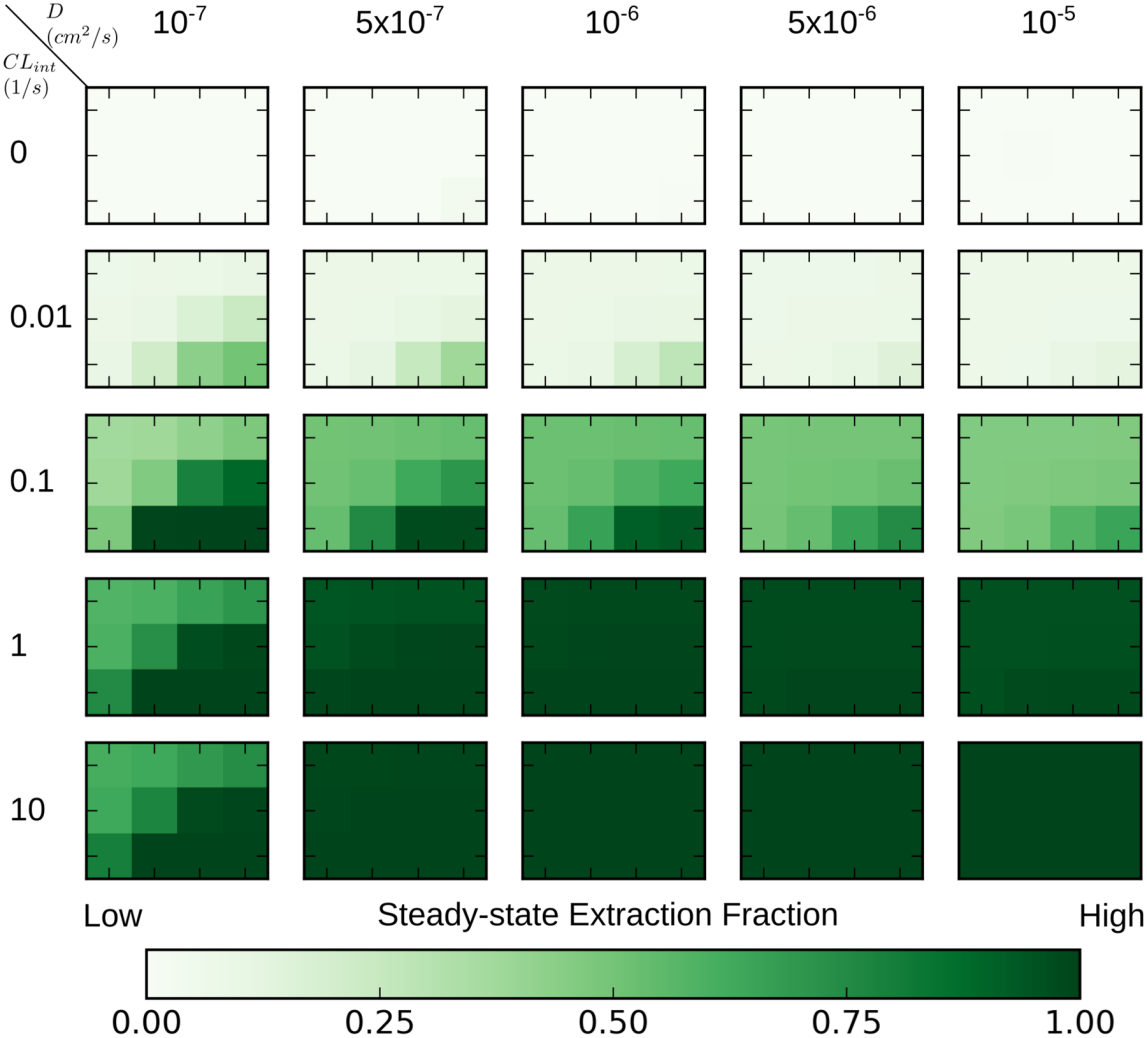
Heat maps of extraction fractions of the xenobiotic at steady state in PERSISTENT simulations with various strength of transport and metabolism. The complete heat map consists of 25 smaller heat maps, termed *constituent heat maps*. A *constituent heat map* contains 12 different (*α*, *β*) pairs, each of which represents varying strength of active transport (refer to Fig 8 for details on the range of (*α*, *β*) pairs). Diffusive trans-membrane rate constants increase from left to right, which represents increasing passive transport. Intrinsic clearance *CL*_*int*_ increases from top to bottom, which represents increasing metabolism. Darker color reflects greater extracted fraction.

We also examined the steady-state extracted fraction of xenobiotic in **PERSISTENT** simulations as a indicator of pan-lobular exposure to the xenobiotic (Fig 11). The extracted fraction is the fraction of the xenobiotic extracted from a portion of blood during its transit through the virtual liver lobule, which, at steady state, is calculated as the ratio of the difference between entering and exiting xenobiotic concentrations over the incoming xenobiotic concentration;

$$\begin{array}{c} \frac{\left[x_{in}\right]-\left[x_{out}\right]}{\left[x_{in}\right]} \end{array}$$ (8)

The larger the extracted fraction, the greater the overall exposure to the xenobiotic is for the entire virtual liver lobule.

Overall, the heat map of steady state extracted fractions (Fig 11) is similar to the descriptors of steady state exposure (Figs 8 and 9). Generally, simulations that displayed *lobular-wise uniform*, *preferentially peripheral* and *preferentially periportal* exposure patterns, had low (0∼30%), intermediate (30%∼70%) and high (70%∼100%) extracted fraction, respectively.

Lastly, as described in Section 2.11 in S1 Text, we explored time series of the xenobiotic partition between the parenchyma and the blood within the virtual liver lobule, as an alternative illustration of xenobiotic extraction and overall hepatic exposure. The time series results lead to the same conclusions as to the parameter domains that lead to the particular exposure pattern, as did the studies outlined above. The only significant difference is the higher periportal exposure at early time points in simulations with rapid transport.

## Discussion

### Summary and significance

We developed a computational model of a biologically plausible CV-centered ***NET*** hepatic lobule. In contrast to the often used ***PIPE*** representations, the ***NET*** lobule predicts diverse transit times and blood flow velocity distributions similar to what is observed *in vivo* [49]. We were particularly interested in regional differences in the xenobiotic distribution that are dependent only on the complex blood flow pattern in the ***NET*** hepatic lobule and did not impose any zonation of transport or metabolic capabilities. Using our ***NET*** lobule model, we simulated the micro-dosimetry of a xenobiotic emergent from cellular transport, metabolism, and blood flow velocities. We see the emergence of tissue-level spatio-temporal patterns of exposure and blood-parenchyma partitioning of the xenobiotic. We further investigated how variations of active and passive transport and metabolic rates affected the spatially dependent exposure of xenobiotic across the lobule. We exhaustively explored the parameter spaces of our four governing parameters for transport (*α*, *β*, and *D*) and metabolism (*CL*_*int*_), which resulted in a range of uptake and metabolism from extremely low (single pass clearance of less than 1%) to extremely high (single pass clearance ∼100%). We observed both central-to-peripheral and axial-to-facial gradients in the xenobiotic distributions and metabolism. Based on the observed gradients, we identified three categories of spatial patterns which, as discussed below, are reminiscent of *in vivo* liver metabolic and necrotic patterns of xenobiotics. In no simulations, other than the trivial passive only without metabolism ***PULSE*** simulations, did we observe a highest pericentral exposure.

Our virtual lobule model and microdosimetry simulations are novel in several aspects. First, we designed and built a ***NET*** virtual mouse liver lobule that is structurally similar to the *ex* *vivo* mouse lobule. Our lobule structure was based on our and other’s parameters derived from microscopic analysis of mouse livers. In particular, the diameters of sinusoids, volume fraction of sinusoids versus hepatocytes, interfacial area of the sinusoid-parenchyma interface and average branching angle of sinusoid junctions [25, 36, 37]. Second, the virtual liver lobule model allowed us to calculate local blood flow velocities. The calculated average blood flow velocities and total perfusion rates were in agreement with measured values reported in literature for mouse [45, 46]. Third, we employed a transport and metabolism model that calculated the distribution, uptake and metabolism across the virtual lobule given the lobule’s geometry and blood flow characteristics. Our simulations predicted the various spatial patterns of hepatic exposure to the xenobiotic based on a set of characteristics (parameters) describing the xenobiotic. Fourth, we identified domains in the transport and metabolism parameters that resulted in different spatial patterns of hepatic exposure. Based on these simulated spatial patterns and corresponding parameter domains, we provide a set of metrics that can help select a suitable model representation of a liver lobule (***BOX***, ***PIPE*** and ***NET***) for a given xenobiotic based on the estimates of transport and metabolism properties of that xenobiotic and the level of details (e.g., microdosimetry, spatial variation in liver damage) expected from the model.

### Diverse blood flow transit times in simulated sinusoid networks

**Fig 12:**
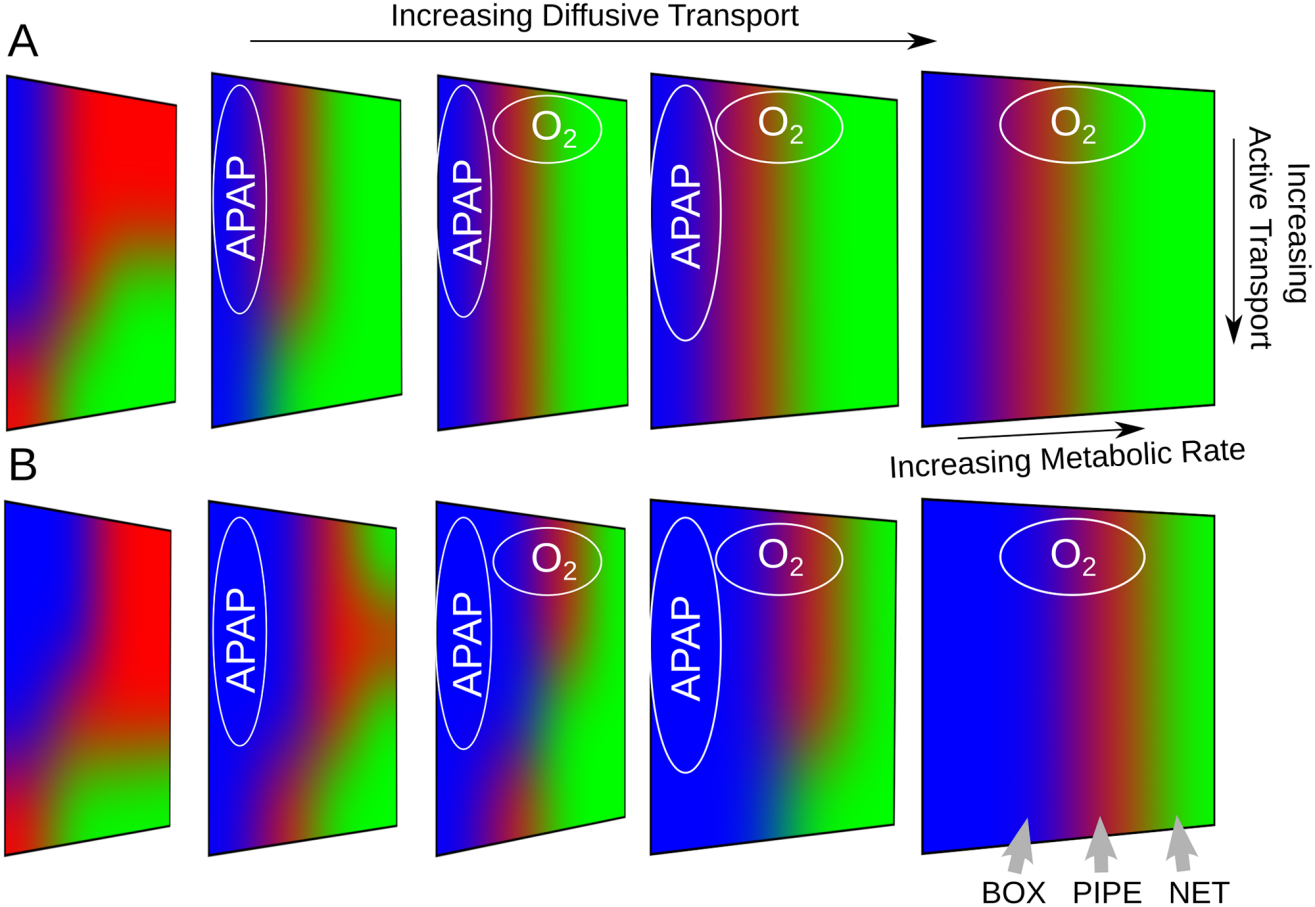
Summary map of appropriate *in silico* model representation of the liver. Two criteria determine if a liver lobule representation is appropriate: Precision of describing microdosimetry and computational efficiency. Five colored maps correspond to different strengths of passive transport, increasing from left to right column. In each colored map, direction from left to right corresponds to increasing metabolism and direction from top to bottom refers to increasing active transport. Color codes the appropriate model representation: blue–**BOX**; red–**PIPE**; green–**NET**. Since this is a three parameter space with color, individual slices of the space are shown horizontally and the colored maps are rotated for better visualization. The conditions for the model choices are as follows: If azimuthal discrepancy is greater than 25% (A) or 100% (B), **NET** is appropriate (green regions). If azimuthal discrepancy is smaller than 25% (A) or 100% (B) and radial discrepancy is larger than 25% (A) or 100% (B), **PIPE** is appropriate (red regions). Otherwise, BOX is appropriate (blue regions). Predicted parameter domains for oxygen (“*O*_2_”) and acetaminophen (“*APAP*”) are marked.

The virtual liver lobule has complex blood flow velocity distributions and diverse transit times of blood along different flow paths. Flow paths along PT–to–CV axes show greater flow velocities and thus smaller transit times than flow paths from PT via a facial region to the CV (Fig 4). This result reflects experimental observation of heterogeneous blood flow velocities in *in vivo* mouse liver [49]. As best illustrated in **PULSE** simulations with low xenobiotic extraction, pulses of the xenobiotic split into separate flow paths with quite different speeds (see the top row in Fig 12 in S1 Text).

Diverse blood flow transit times contribute to spatial patterns of hepatic exposure to the xenobiotic. In **PULSE** simulations, if transporter-mediated extraction of the xenobiotic from the blood to the hepatocytes is the sole mechanism for uptake, and those transporters are easily saturated (*α* = 0.01), then the xenobiotic predominantly accumulates in the pericentral region (Fig 17 in S1 Text). This effect results from different *cumulative uptake durations* experienced by peripheral versus pericentral cells. Increasing the pulse size width diminishes this effect.

In **PERSISTENT** simulations, when active and passive transports are both weak, the azimuthal gradient of the xenobiotic is smaller than the radial gradient. Facial and peripheral cells have larger cumulative uptake durations than pericentral cells do, and this partially compensates for their positional disadvantage for uptake and diminishes the azimuthal differences in hepatic exposure.

### Distribution and metabolism gradients describe the behavior of different xenobiotics

Radial and azimuthal metrics, central-to-peripheral ratio *γ*_*cp*_ and axial-to-facial ratio *γ*_*af*_, respectively, characterize the spatial pattern of hepatic exposure to the xenobiotic. The use of these two metrics allows us to categorize the range of spatial patterns into *lobular-wise uniform* (0.75 < *γ*_*cp*_ ≤ 1.25, 0.75 < *γ*_*af*_ ≤ 1.25), *preferentially peripheral* (*γ*_*cp*_ ≤ 0.75, 0.75 < *γ*_*af*_ ≤ 1.25), and *preferentially periportal* (*γ*_*cp*_ ≤ 0.75, *γ*_*af*_ > 1.25). Heat maps of radial and azimuthal descriptors (Figs 8 and 9), created across a wide range of transport and metabolism parameters of the imaginary xenobiotic, provide a clear depiction of *which subset of xenobiotic properties are responsible for the emergence of particular spatial patterns of hepatic exposure*, as summarized in Fig 10.

Categorization of hepatic exposure to the xenobiotic enables us to make predictions on the required biophysical properties of *a blood-born endogenous signal that hepatocytes can use to determine their zonal identities*. Several hypotheses have been proposed regarding signals for regulation and maintenance of metabolic zonation in the liver, including the gradient in serum oxygen concentration [15] and the *wnt/β-catenin* signalling system [18, 50]. In the first case, the hypothesis is that a blood-borne signal, with a spatially varying adsorption, provides a zonation signal. Thus, the prerequisite for an endogenous, blood borne compound to act as the “zonation” signal, is to acquire certain transport and metabolism properties that lead to either *preferentially peripheral or preferentially periportal* hepatic exposure. Examination of Figs 8–10 suggests that an endogenous compound that has moderate to strong metabolism will result in significant variation in hepatic exposure, which could be used as a zonation signal.

Using our model, it is possible to evaluate if oxygen could act as a zonation signal. Oxygen has complicated kinetics because the serum concentration of oxygen is coupled to oxygen bound to hemoglobin in the red blood cells. Thus, there is a buffering effect on the serum oxygen concentration and the serum oxygen concentration gradient is not as steep as might be expected. Oxygen in the serum is expected to be absorbed rapidly by the hepatocytes via passive transport only, so *D* is intermediate to large and *β* is zero. Studies using isolated hepatocytes suggest that the rate of oxygen consumption per cell is in the range of 0.02 ∼ 0.07 *mmol* ⋅ *L*^−1^ ⋅ *s*^−1^ [51, 52]. Assuming all hepatocytes within the liver lobule have the same oxygen consumption rate this corresponds to a moderate to strong metabolic rate (0.1/*s* < *CL*_*int*_ < 1/*s*) in our model. Examining the simulation results for these estimated oxygen parameters indicate a zonal hepatic exposure, as shown in Fig 8, bottom three rows where *γ*_*cp*_ ≈ 0.5. Therefore, this model, with an albeit simple model for oxygen kinetics in the blood, predicts that blood-borne oxygen could be a “zonation” signal since it shows a zonal distribution pattern.

### Relevance to *in silico* modeling of the liver

As introduced in Fig 2, *in silico* representations of liver lobules need to address a balance between computational efficiency, spatial resolution and accuracy. Using the categorization of hepatic exposure to xenobiotics described herein, we provide a set of scenarios that can be used to select one of the model representations of the liver (***BOX***, ***PIPE*** or ***NET***), for modeling a particular xenobiotic, *if the purpose of a particular study is to have optimal computational efficiency without losing precision in characterization of the hepatic exposure*. For example, ***BOX*** representation lacks precision in description of the liver architecture, so it is not recommended for modeling a xenobiotic that has a zonal hepatic exposure, despite its advantage in computational efficiency. Assuming that any active transporters and metabolic capabilities are evenly distributed throughout the lobule, the domains of xenobiotic properties corresponding to suitable model representations of the liver are described in Fig 12 and as:

- For compounds with low extraction or weak metabolism,—hepatic exposure is uniform across the lobule. In these situations, ***BOX***, ***PIPE*** and ***NET*** are equally competent and precise for describing the xenobiotic exposure. Considering computational efficiency, ***BOX*** is appropriate.
- For compounds with (1) moderate metabolism and moderate to strong passive transport regardless of the strength of active transport, or (2) moderate to strong metabolism but weak active and passive transport,—hepatic exposure to xenobiotics displays radial gradients but insignificant azimuthal gradients. Compounds of this type show preferentially periportal exposure. In these situations, ***BOX*** representation is not appropriate due to its imprecision in characterization of microdosimetry, but ***PIPE*** and ***NET*** are both capable of describing hepatic exposure. Considering computational efficiency, ***PIPE*** is appropriate. However, it should be noted that ***PIPE*** models misrepresent the relative number of peripheral versus central hepatocytes and sinusoids, which needs to be accounted for.
- For compounds with (1) strong metabolism and moderate to strong passive transport regardless of the strength of active transport, or (2) moderate to strong metabolism, strong active transport but weak passive transport,—hepatic exposure to xenobiotics has both radial and azimuthal gradients. Compounds of this type show preferentially periportal and facial exposure patterns. Only ***NET*** representations can adequately describe this pattern of hepatic exposure. Thus, ***NET*** is appropriate.
- We did not observe any xenobiotic parameter that gave preferentially pericentral exposure. This indicates that pericentral metabolism or toxicity is due to zonal differences in metabolic or toxic responses and not a result of the complex blood flow pattern giving rise to higher pericentral exposure.

However, the *goals* of a particular computational model can override the guidelines listed above. For example, if zonal liver functions or damages is irrelevant to the questions a particular study addresses, then the ability to properly characterize the emergence of zonal hepatic exposure is not needed and ***BOX*** representation is sufficient. Furthermore, it is important to recall that both our ***PIPE*** and ***NET*** simulations do not have zonal gene expressions and zonal variation in transporters or metabolism may overwhelm the properties emergent from the blood flow and the sinusoidal network topology.

Locating the transport and metabolism domains for oxygen in the heat maps suggest it could acts as a blood-borne “zonation” signal as discussed above. Since that includes zonal hepatic exposure to oxygen, ***PIPE*** or ***NET*** representation are suitable (domain labeled “*O*_2_” in Fig 12).

Another example is acetaminophen (*APAP*), which has simpler partitioning kinetics in blood than oxygen. APAP is rapidly absorbed into cells (passive transport is rapid and active transport is weak to moderate). The metabolic clearance of APAP is quite slow compared with transport rate (*CL*_*int*_ is small). Locating this parameter region in the summary maps in Fig 12 indicates that ***BOX*** representations are sufficient to describe the hepatic exposure to APAP (domain labeled “*APAP*” in Fig 12). This result also suggests that hepatic exposure to APAP is uniform in the context of anastomotic sinusoid network and complex blood flow, and thus to explain the centrilobular necrosis induced by APAP requires *imposing metabolic zonation*. In a modeling study that includes zonation of metabolism within liver lobules of APAP and aims to study zonal liver damage, ***BOX*** representation is not appropriate and ***PIPE*** or ***NET*** representation are recommended.

A final example is the behavior of a blood-borne macromolecule such as a protein hormone. These macromolecules bind to cell surface receptors and enter cells via active transport. In this case, *α* and *β* describe the Michaelis-Menten kinetics for receptor-binding and *CL*_*int*_ describes the internalization and/or destruction of the protein. In Fig 12 a protein hormone, with high, but saturable binding affinity and fairly rapid destruction, would be located in the bottom right region of the left most panels, suggesting that a ***NET*** model is needed to properly reflect the zonal distribution of a blood borne protein signal.

In this work we address two questions key to understanding and predicting the functioning of, and damage to, the liver lobule. First, is the complex lobular blood flow pattern sufficient to explain zonal exposure and damage? Second, what characteristics of a xenobiotic make detailed lobule flow modeling, in a spatially accurate lobule model, a requirement to predict the pattern of zonal exposure? Our modeling results suggest that the complex blood flow pattern contributes significantly only in cases where the xenobiotic is rapidly absorbed and metabolized leading to preferentially periportal exposure. In no cases did we observed preferentially pericentral exposure. Therefore, for compounds that show pericentral damage that damage is due to zonal differences in metabolism and/or transport and not to the complex flow pattern.

### Limitations

The model does not account for certain structural aspects of the mouse liver lobule. For example, the virtual liver lobule has fewer inter-sinusoidal connections than the mouse liver lobule. This is partly due to the lack of inter-plane connections between the simulated planar sinusoid network and the un-modeled planes above and below. The simulated sinusoid network also omits in-plane azimuthal connections, because these connections would contain very low blood flow due to small azimuthal pressure gradient.

Our flow model does not explicitly treat the hematocrit *H*_*c*_ (the volume fraction of blood that is red blood cells) nor the possibility that the *H*_*c*_ varies across the sinusoidal network. We have used the *Netflow* [38, 47] capillary blood flow calculator to predict *H*_*c*_ across our typical in silico lobule. As shown in Section 2.1 of S1 Text, the predicted *H*_*c*_ across our typical in silico lobule ranges from 0.21–0.64 for an input *H*_*c*_ of 0.45. Also, our model does not address the phenomenon of intermittent blood flow in sinusoid segments [45] and instead simulates constant blood flow rates.

The model makes several simplifications in terms of transport and metabolism processes. For example, the model does not include active export process (such as exporters like MDR/PGP) that pump xenobiotics from the hepatocytes back into the blood or transfer into the bile canaliculi. In addition, we do not include hepatocyte–to–hepatocyte transfers via gap junctions, which may allow more rapid intercellular transfer. In addition, the model *does not incorporate zonal variations in transport or metabolic processes*, which will affect the pattern of hepatic exposure to xenobiotics.

## Supporting information

S1 Text Supplementaryi information. Extensive supplementary information detailing the model development, implementation and testing. Particular sections in this file are referenced in the main text. This file also includes descriptions of the animations in S1 Animations and the source code in S1 Code.
(PDF)

S1 Animations Simulation animation files. Animation files, for details see Section 3 of S1 Text.
(ZIP)

S1 Code Source code. Source code files, for details see Section 4 of S1 Text.
(ZIP)
