# Supplementary material for: Modeling of xenobiotic transport and metabolism in virtual hepatic lobule models
Source: PLoS One. 2018 Sep 13;13(9):e0198060. doi: 10.1371/journal.pone.0198060 (PMC6136710; doi:10.1371/journal.pone.0198060)
Supplement: S1 Text — Extensive supplementary information detailing the model development, implementation and testing. Particular sections in this file are referenced in the main text. This file also includes descriptions of the animations in S1 Animations and the source code in S1 Code. (PDF) [file pone.0198060.s001.pdf]

Supplementary Material for:

Modeling of Xenobiotic Transport and Metabolism in Virtual Hepatic Lobule Models

Xiao Fu[1,2], James P. Sluka[1,3*], Sherry G. Clendenon[1,3], Kenneth W. Dunn[4], Zemin Wang[5], James E. Klaunig[5], and James A. Glazier[1,3]

1 Biocomplexity Institute, Indiana University, Bloomington, IN, USA

2 Department of Physics, Indiana University, Bloomington, IN, USA

3 Department of Intelligent Systems Engineering, Indiana University, Bloomington, IN, USA

4 School of Medicine, Indiana University, Indianapolis, IN, USA

5 School of Public Health, Indiana University, Bloomington, IN, USA

* Author for correspondence: James P. Sluka (e-mail: jsluka@indiana.edu)

Contents

1 Supplementary Information for *Methods* **5**

1.1 Histological studies of mouse liver 5

1.2 Confocal imaging of rat and mouse liver sinusoids 5

1.3 Construction of the ***PIPE*** virtual liver lobule 8

1.4 Construction of the ***NET*** virtual liver lobule 8

1.5 Mechanistic model of transport and metabolism 8

2 Supplementary Information for *Results* **13**

2.1 Comparison of Flow Calculations in CC3D with *NetFlow* of Priese and Secomb 13

2.2 Flow Calculations and Microdosimetry Simulations with Varied Sinusoids Structure 16

2.3 Flow Calculations and Microdosimetry Simulations with Varied Lobule Size 18

2.4 Flow Calculations and Microdosimetry Simulations with Varied PT-CV Pressure Drop 18

2.5 Descriptors of Hepatic Exposure within a *PIPE* Lobule in Active Transport Only PULSE Simulations 20

2.6 Hepatic *NET* Exposure to PULSE Xenobiotic 20

2.7 Descriptors of Hepatic Exposure within a *NET* lobule in Active Transport Only PULSE simulations with Varying Pulse Sizes 31

2.8 Lobular Partition of the Xenobiotic in PULSE Simulations 33

2.9 Descriptors of Hepatic Exposure within a *NET* lobule in Active Transport Only PERSISTENT simulations 35

2.10 Variability in Calculated Hepatic Exposure from Replicate Simulations 36

2.11 Lobular Partitioning of the Xenobiotic in PERSISTENT Simulations 36

3 Animations **40**

4 Source Files **42**

5 References **44**

List of Tables

1 **S1 Table 1.** Quantitative Results for Six (v0–v5) Different Constructed Sinusoid Networks. . 16

2 **S1 Table 2.** Quantitative Results with Different Lobule Sizes. 19
3 **S1 Table 3.** Quantitative Results with Varied PT–to–CV Pressure Drop ΔP 20
4 **S1 Table 4.** Variability in Replicate Simulations: Average and Standard Deviations for 50 Replicate Simulations 36
5 **S1 Table 5.** Animations for selected **PULSE** and **PERSISTENT** simulations. 41
6 **S1 Table 6.** Files included in the zip file S1 Source. 42
7 **S1 Table 7.** Parameters in "pDictLiverXeno.csv" for generation of ***NET*** simulations. . . . 43

List of Figures

S1 Fig 1: H&E staining of normal and necrotic mouse liver.. 6
S1 Fig 2. : Rat liver lobule from confocal microscopy. 7
S1 Fig 3: A ***PIPE*** virtual liver lobule. 9
S1 Fig 4: Schematics of sinusoid network construction. 10
S1 Fig 5: A ***NET*** virtual liver lobule. 11
S1 Fig 6: Schematics of advection, transport and metabolism in a small virtual liver section. . . . 12
S1 Fig 7: Comparison of flow calculations in CC3D and using NetFlow. 14
S1 Fig 8: Calculations of H_c in using NetFlow. 15
S1 Fig 9: Impact of varying structure of sinusoid network on hemodynamics and hepatic exposure. 17
S1 Fig 10: Impact of lobule size on hemodynamics. 19
S1 Fig 11: Heat maps of central-to-peripheral and axial-to-facial ratios within the ***PIPE*** lobule at $\boldsymbol{t = 20\ s}$ in active transport-only **PULSE** simulations. 21
S1 Fig 18: Heat maps of central-to-peripheral ratios within a *NET* lobule at $\boldsymbol{t = 20\ s}$ in active transport-only **PULSE** simulations with different pulse sizes. 32
S1 Fig 19: Lobular partition of the xenobiotic over time in passive transport-only and active transport-only **PULSE** simulations. 34
S1 Fig 20: Heat maps of central-to-peripheral and axial-to-facial ratios within a ***NET*** lobule at $\boldsymbol{t = 20\ s}$ in active transport only **PERSISTENT** simulations. 35
S1 Fig 21: Lobular partition of the xenobiotic over time in **PERSISTENT** simulations without metabolism. 37
S1 Fig 22: Lobular partition of the xenobiotic over time in **PERSISTENT** simulations with both active/passive transports and metabolism ($CL_{int} = 0.01/s$). 38

S1 Fig 23: Lobular partition of the xenobiotic over time in **PERSISTENT** simulations with both active/passive transports and metabolism ($CL_{int} = 0.1/s$). 39

1 Supplementary Information for *Methods*

1.1 Histological studies of mouse liver

Mice were housed in the AAALAC certified animal facility at Indiana University, Bloomington, Indiana. All animals were maintained in accordance to the Guide for the Care and Use of Laboratory Animals from the Institute of Laboratory Animal Research [1] and treatment protocols were approved by the Institutional Animal Care and Use Committee at Indiana University (IACUC)(permit number 14-012). Mice, after 12 hrs fasting, were treated with APAP at doses of 0 (control), 10, 100, or 250 mg/kg BW by IP injection. Mice were sacrificed 0.25 to 48 hrs after treatment in accordance with animal care procedures. A portion of the liver was fixed in 10% neutral buffered formalin and sectioned for histopathology analysis by hematoxylin and eosin (H&E) staining. The rest of the liver was snap frozen in liquid nitrogen and stored at –80C.

Images of H&E stained liver sections are shown below in S1 Fig 1. In a H&E stained liver section, cytoplasm appears pink and cell nucleus purple. In normal liver, hepatocytes have large round nuclei (S1 Fig 1A). In contrast, following treatment with a toxic dose of acetaminophen, hepatocytes near the central vein undergo necrosis as evidenced by a loss of nuclei (S1 Fig 1B).

1.2 Confocal imaging of rat and mouse liver sinusoids

Rats were maintained using protocols approved by the IACUC (permit number 10971). Rats were euthanized by inhalation of anesthetic gases (isoflurane) followed by bilateral pneumothorax, as well as exsanguinations as physical means to ensure death and as required by IUCAC. This minimizes animal distress and is quick, effective and efficient. This method is consistent with the recommendations of the AVMA Guidelines on Euthanasia.

A portion of a rat liver lobule sinusoid network is shown in S1 Fig 2. An animated file, giving better depth perception of this sinusoid network, is in file Rat_liver_sinusoids-1.mpg.

We labeled 300 μm thick sections of rat liver that had been perfusion fixed in 4% paraformaldehyde in PBS with FITC conjugated lens culinaris agglutinin (Vector Laboratories, Burlingame, CA) according to manufacturer instructions. Following labeling we optically cleared the tissue sections using benzyl alcohol-glycerol [2]. We acquired 3D mosaic images using a Leica SP2 MP microscope system (Leica Microsystems, Buffalo Grove, IL). We stitched these images, then segmented the vasculature and acquired tissue vasculature metrics using FIJI ImageJ [3, 4].

The volume fraction of sinusoids in this rat lobule, excluding the CV and PT, is 10.4% ± 1.1%. Note that

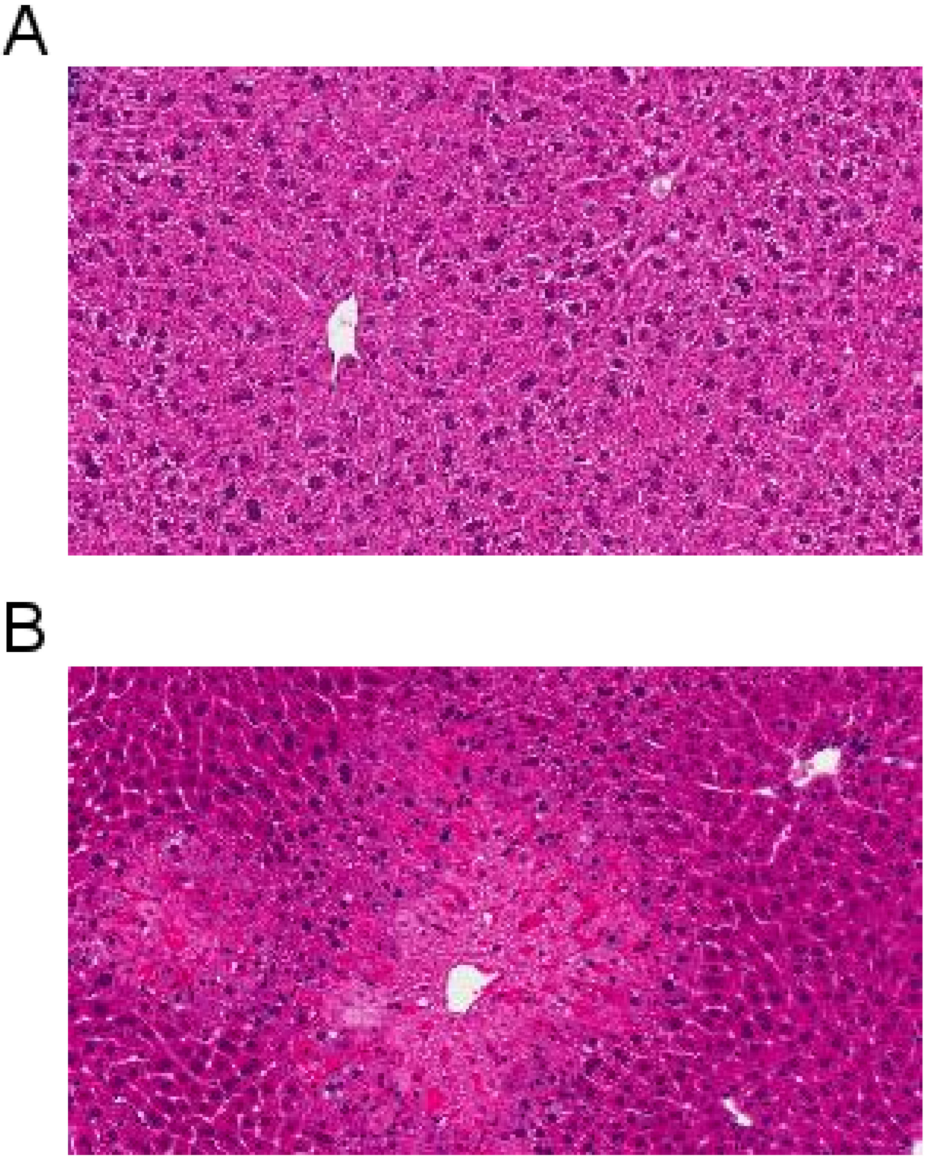

S1 Fig 1. H&E staining of normal and necrotic mouse liver. Portions of mouse liver lobules stained using hematoxylin and eosin (H&E). (A) Normal liver section. A central vein (**CV**) is located near the center of the image. (B) Necrotic liver section 12 hours following administration of toxic dose of acetaminophen. A central vein (**CV**) is located near the center of the image. A portal triad (**PT**) is located near the upper right corner of the image. Massive necrosis of hepatocytes are evident surrounding the **CV**.

the volume measured for the sinusoid is the luminal volume. Previous studies reported values for mice $15.3 \pm 3.9\%$ [5] and for rats $10.6 \pm 4.5\%$ [6], though it is not always clear if the number refers to the lumenal volume or the lumen+endothelial volume.

Ex vivo sinusoid networks show a small variation in sinusoids diameters, with the largest diameters occurring near the PT and CV and smaller diameters in the mid-lobular region. In addition, the *ex vivo*

samples have a somewhat more densely connected network in three dimensions and it is not uncommon to observe nodes with four or more connections. We also measured the average sinusoid-hepatocyte interfacial area per unit parenchymal volume S_{int}, which influences transport of xenobiotic in our model. S_{int}, in this rat liver, was found to be 0.143 ± 0.019 $\mu m^2/\mu m^3$.

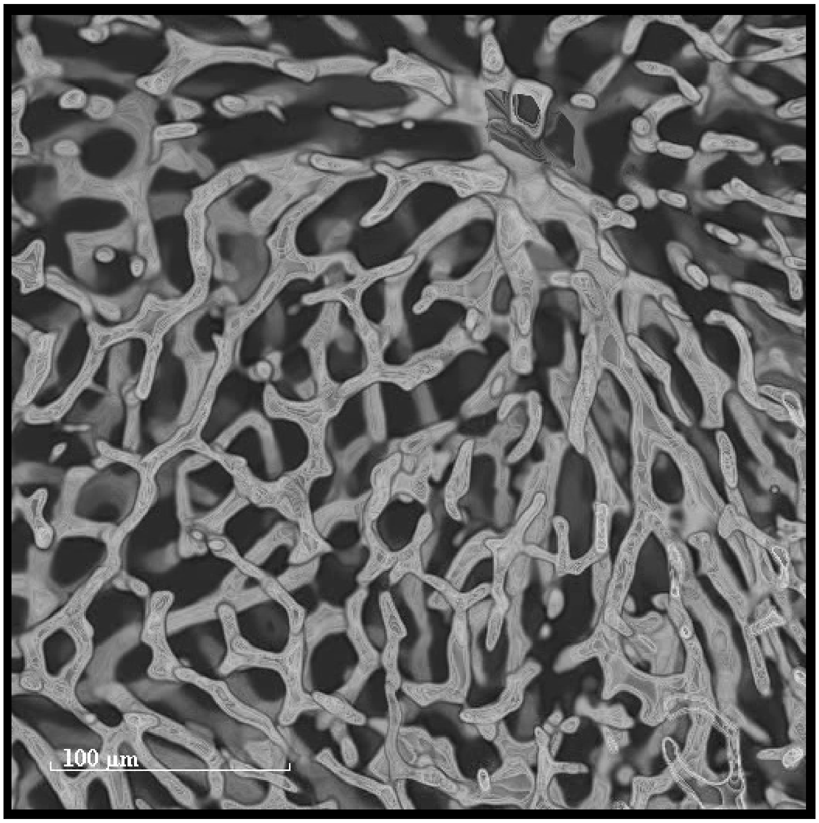

S1 Fig 2. Rat liver lobule from confocal microscopy. Portion of a 3D rat liver lobule showing the sinusoid network. A central vein (**CV**) is located near the upper right portion of the image. The scale bar is $100\mu m$ and the typical sinusoid is $\approx 8\mu m$ diameter. The thickness of the imaged region was 50 μm.

1.3 Construction of the *PIPE* virtual liver lobule

The ***PIPE*** virtual liver lobule consists of six parallel arrays of hepatocytes along six parallel "pipe"-like sinusoids (S1 Fig 3). The virtual liver lobule has dimensions of 240 $\mu m \times 120$ $\mu m \times 20$ μm. PT and CV are not explicitly constructed in PIPE lobule. Instead, we used an extended "Wall" regions of the lobule to represent area that should be occupied by PT and CV (S1 Fig 3B). We modeled no diffusive or active transport processes between SINs and "Wall", and no metabolism within the "Wall". We applied periodic boundary conditions in two dimensions perpendicular to flow direction.

1.4 Construction of the *NET* virtual liver lobule

In the Main Text, we described the steps of sinusoids network construction including determination of network node locations and creation of network edges. Here, we use a schematic to illustrate the processes (S1 Fig 4). We started by constructing the hexagonal boundary of a CV-centered virtual liver lobule including six PTs and peripheral sinusoids. We then added the central vein at the center of hexagon and drew virtual concentric rings surrounding central vein with radial spacing of one hepatocyte size (S1 Fig 4A). Next, on each virtual ring, we randomly selected a point as a node location and determined other node locations by "walking" on the ring with a step of one HEP size. We also subdivided each of six peripheral sinusoids by unit of one HEP size to determine perimeter node locations (S1 Fig 4B). Then, we started from the innermost ring and connected each node on the ring to the nearest node on the next outer ring to form an edge. Nodes on the the outermost ring connected to the nearest nodes on peripheral sinusoids or to PT nodes (S1 Fig 4C). Lastly, we determined locations of cell centers of HEPs by selecting the middle point between any two nodes on the same virtual rings, which will later become the source voxels of HEPs in CC3D (S1 Fig 4D).

After creation of the sinusoid network, we converted the model into a 3D spatial model in CC3D. PTs and the CV are converted into pixelated cylinders as are the sinusoid segments. The sinusoids have a diameter of $8\mu m$. The thickness of the virtual liver lobule slice is the thickness of one hepatocyte, 20 μm. Individual heptocytes developed from the one-voxel cell centers created in the layout algorithm, to fill the remaining space between sinusoid network. A typical ***NET*** virtual liver lobule in the model is shown in S1 Fig 5.

1.5 Mechanistic model of transport and metabolism

Figure 3 in the main text describes mathematical forms of the mechanistic model of xenobiotic advection, passive transport, active transport and metabolism. This section presents a more detailed schematic of these processes (S1 Fig 6). The spatial configuration of hepatocytes and sinusoids in a zoomed view of a virtual

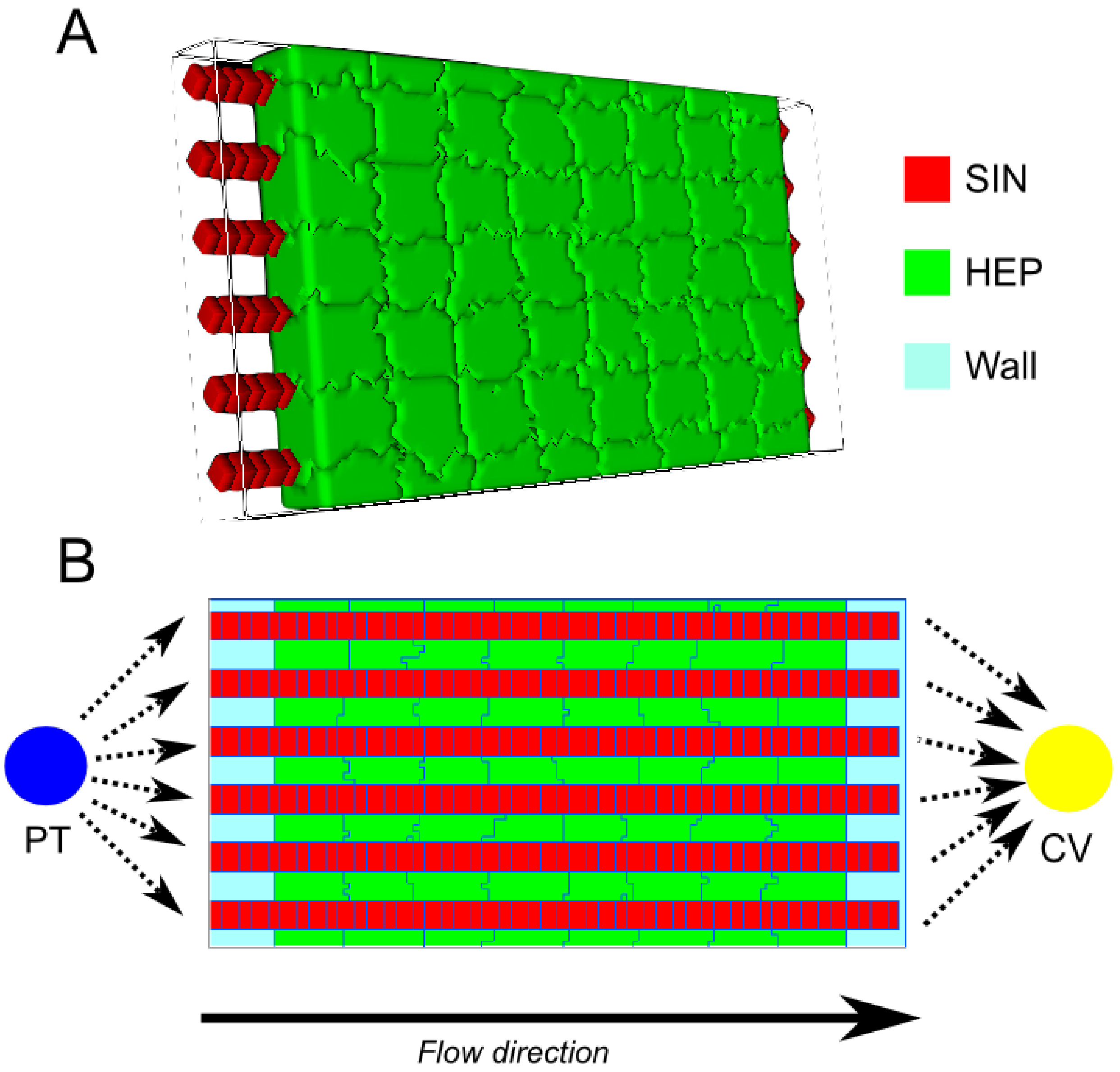

S1 Fig 3. A *PIPE* virtual liver lobule. Upper image is a 3D representation of the ***PIPE*** virtual liver lobule. Lower image is 2D cross-sectional view at the plane of sinusoids. The lobule's dimensions are 120 $px \times 60$ $px \times 10$ px, corresponding to 240 $\mu m \times 120$ $\mu m \times 20$ μm.

liver lobule is shown in S1 Fig 6A. The passive (diffusive) transport of the xenobiotic is modeled bidirectionally at the interface between sinusoid blocks (SINs) and hepatocytes (HEPs) and at the interface between neighboring HEPs. Active transport (transporter mediated) of the xenobiotic is modeled unidirectionally at the interface between SINs and HEPs, from the former to the latter. Metabolism of xenobiotic is modeled within individual HEPs.

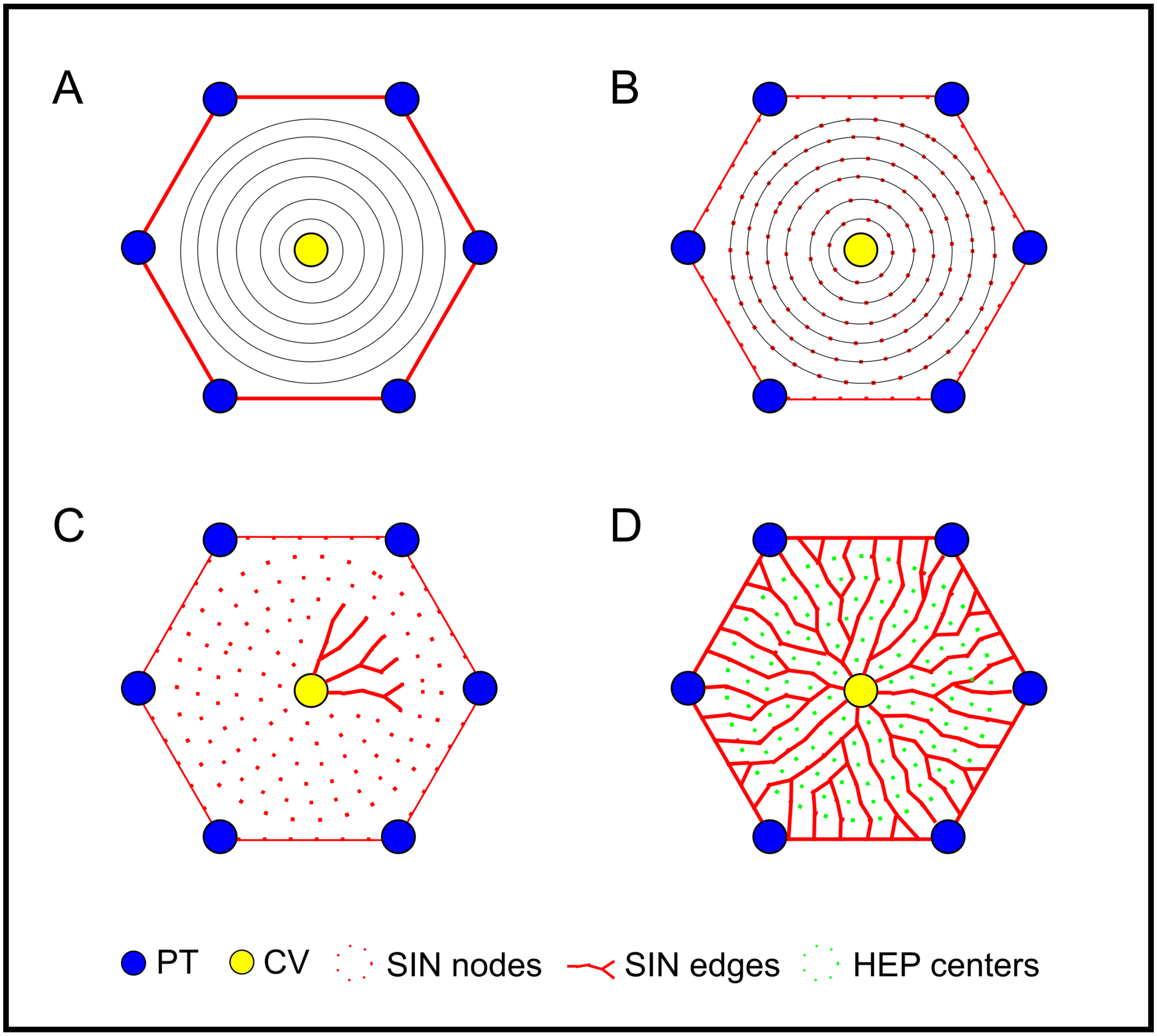

S1 Fig 4. Schematics of sinusoid network construction. (A) Construct peripheral sinusoid segments by connecting adjacent PTs (red). (B) Determine positions of network junctions on concentric rings. (C) Connect junctions to nearest junction on the next inner ring. (D) Create a one-voxel HEP between junctions on each rings.

The topological configuration of conveyor belt blocks (**CB**s) and junctions associated with the sinusoids network are shown in S1 Fig 6B. Advection of blood-borne xenobiotic is modeled unidirectionally from an upstream CB to the downstream one on the same segment. Converging segments receive the total mass of the xenobiotic in the last CBs of the upstream segments (e.g. segment ik and segment jk) into the first CB of the downstream segment (e.g. segment kr).

SINs, the structural elements of sinusoid segments, have equal length; CBs, the functional element of xenobiotic advection, have different sizes on different segments proportional to blood flow velocity in that

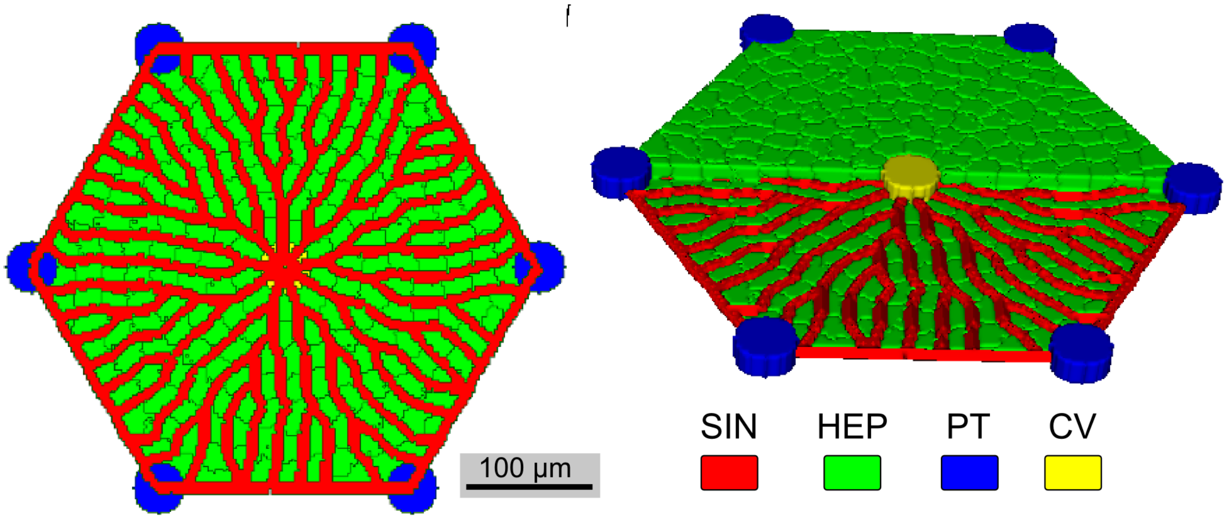

S1 Fig 5. A *NET* virtual liver lobule. A ***NET*** virtual liver lobule constructed in CC3D following procedures described in S1 Fig 4. The image on the left is a 2D cross-sectional view through the plane of sinusoid network. The image on the right is a partial cutaway of the 3D lobule slice. The hexagonal lobule has an edge size of 100 px corresponding to 200 μm and a thickness of 10 px corresponding to 20 μm.

sinusoid segment. Those CBs on a segment that carry blood flow with greater flow velocity have larger size and consequently the operation of "conveying" the xenobiotic one block downstream reflects faster "advection" (proportional to linear blood flow velocity).

To coordinate the advection part of the model, which is calculated at the CB level, with passive/active transport and metabolism, which are calculated at the SIN/HEP level, the model converts the mass of the xenobiotic between SINs and their associated CBs. Each SIN is associated with one or more CBs, which is guaranteed by selecting a sufficiently small time step Δt. In a simulation cycle of advection, transport and metabolism, mass conversion between CBs and SIN occurs twice. Following advection step, the model adds up the masses of the xenobiotic in the associated CBs to give the mass of the xenobiotic in a particular SIN. Then the model calculates passive/active transport among SINs and HEPs and metabolism within HEPs. Following metabolism step, the model adds (or subtracts) the same percentage change of the xenobiotic mass in a particular HEP to (or from) its associated CBs. In the next simulation cycle, all CBs have updated xenobiotic mass that reflects the post-transport level in the blood.

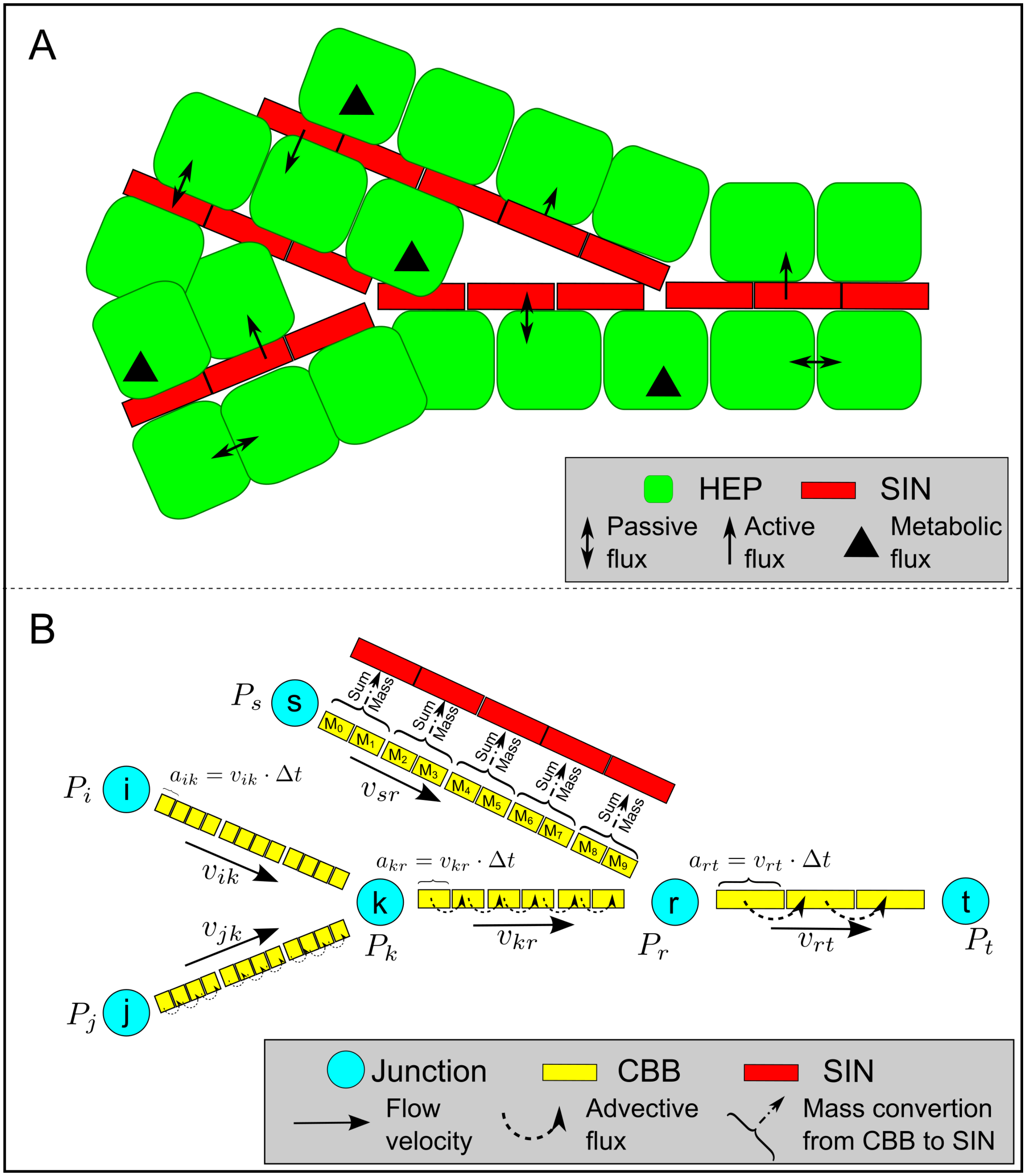

S1 Fig 6. Schematics of advection, transport and metabolism in a small virtual liver section. (A) Spatial configuration of hepatocytes (HEPs, green) and sinusoid blocks (SINs, red) in a schematic representation of a small virtual liver section. Model processes of the xenobiotic illustrated in (A) include passive transport (double-headed arrow), active transport (single-headed arrow) and metabolism (solid triangle). For clarity, only a few representative markers are shown in the diagram. (B) Topological configuration of conveyor belt blocks (CBs, yellow) and junctions (cyan) associated with the sinusoid network in (A). Model process of the xenobiotic illustrated in (B) is the advective flux (curved dashed arrows).

2 Supplementary Information for *Results*

2.1 Comparison of Flow Calculations in CC3D with *NetFlow* of Priese and Secomb

To calculate blood flow rates in the virtual sinusoid network, we wrote a Python module in CompuCell3D (CC3D) [7]. To check its accuracy, we compared calculated flow rates with results using an existing tool NetFlow developed by Pries, Secomb and coworkers for blood flow calculations in capillary (sinusoid) diameter vessels [8, 9]. We found that the correlation between our results in CC3D and those from NetFlow was very good (S1 Fig 7). The dispersion about the regression line is due to the inclusion of hematocrit variation in the NetFlow calculation, whereas the CC3D calculation does not take hematocrit into account. The correlation between the NetFlow and CC3D results is essentially a perfect if the NetFlow calculation is carried out with a hematocrit of zero. In addition to using NetFlow to verify our flow calculations we also used it to explore the hematocrit partitioning across the representative lobule. As shown in S1 Fig 8, using an input H_c of 0.45 the calculated distribution of H_c ranges from 0.21 to 0.64.

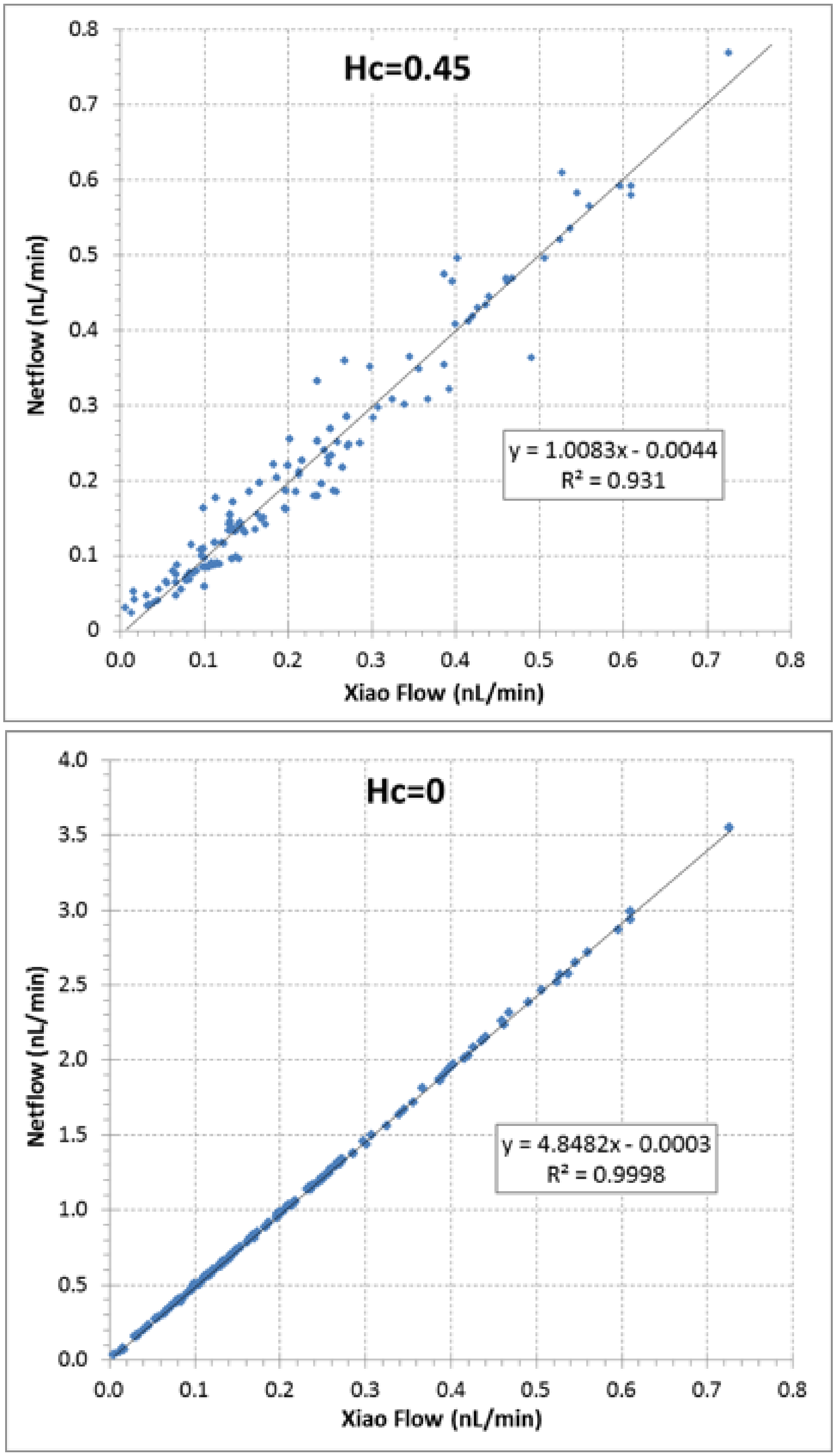

S1 Fig 7. Comparison of flow calculations in CC3D versus NetFlow. Blue dots are calculated blood flow rates in individual sinusoid segments in the ***NET*** virtual liver lobule. Horizontal axis are the flow rates from our calculation in CC3D. Vertical axis are the flow rates calculated using NetFlow [8, 9] for the same network. Upper graph is the NetFlow calculation with hematocrit $H_c = 0.45$ and the lower is the NetFlow calculation with $H_c = 0$.

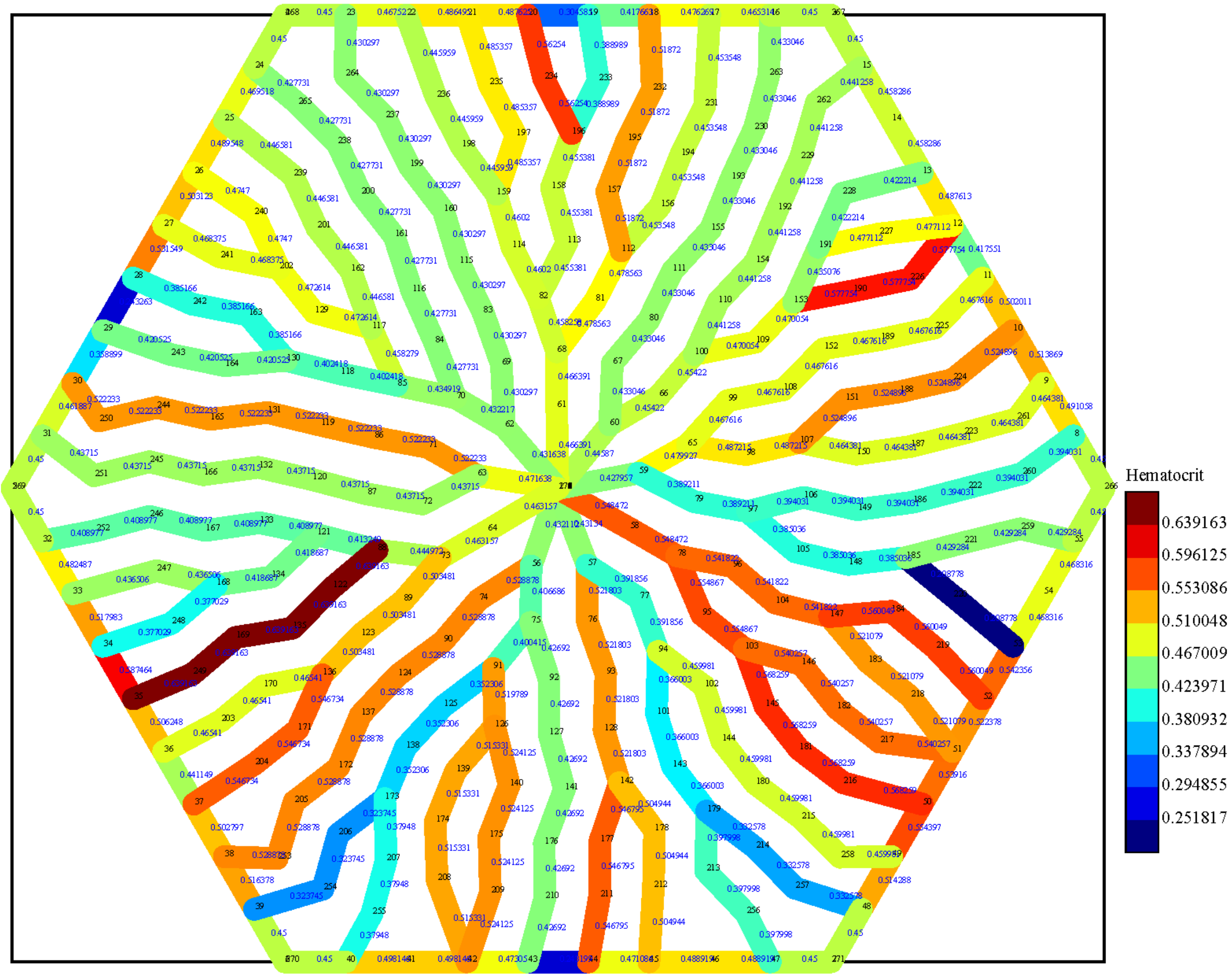

S1 Fig 8. Calculated H_c in the virtual liver lobule. Blood H_c is normally 0.40 to 0.50. Here we calculate the H_c using an input H_c of 0.45 using NetFlow. Black numbers are node IDs and blue numbers are segment H_c. Note that typically the H_c near the PTs and CV are near the input H_c of 0.45. Flows from a face of the hexagon tend to have higher H_c whereas from a vertex (PT) lower H_c.

2.2 Flow Calculations and Microdosimetry Simulations with Varied Sinusoids Structure

To explore the effects of anastomotic patterns of sinusoid network on structural and hemodynamic quantities of the virtual liver lobule as well as hepatic exposure to the xenobiotic, we examine the structural and hemodynamic features of six total lobule slices created using the same construction algorithm but with different anastomotic patterns (S1 Table 1 and S1 Fig 9). All of these constructed liver lobules show similar results in terms of mean hepatocyte volume $\bar{V}_{hep}$, sinusoids volume fraction η_{sin}, average sinusoid-parenchymal interfacial area per unit parenchymal volume S_{int}, mean blood flow velocity $\bar{v}$, and total lobular volume inflow rate $\dot{Q}$. Virtual lobules with different anastomotic patterns show appreciable, though small, difference in hepatic exposure to the xenobiotic, as reflected by different lobular-wise average xenobiotic concentration $\bar{c}_{lob}$ and two descriptors of spatial variation in xenobiotic micro doses γ_{cp} and γ_{af}.

S1 Table 1. Quantitative Results for Six (v0–v5) Different Constructed Sinusoid Networks.

Quantity (unit)	v0	v1	v2	v3	v4	v5	Avg.	±	S.D.	%
$\bar{V}_{hep}$ (μm^3)	8030	8007	8048	8011	8008	7999	8017	±	18	0.2%
η_{sin} (%)	12.86	12.79	12.72	12.67	12.78	12.82	12.77	±	0.07	0.5%
S_{int} ($\mu m^2/\mu m^3$)	0.1218	0.1217	0.1221	0.1249	0.1219	0.1217	0.1224	±	0.0013	1.0%
$\bar{v}$ ($\mu m/s$)	67.52	66.57	66.54	62.53	67.42	64.21	65.8	±	2.0	3.0%
$\dot{Q}$ (pL/s)	97.75	94.86	94.75	90.58	96.61	91.05	94.3	±	2.9	3.1%
$\bar{c}_{lob}$ ($mmol/L$)	1.426	1.118	1.088	1.090	1.159	1.1165	1.17	±	0.13	11%
γ_{cp}	0.1410	0.1394	0.1326	0.1299	0.1403	0.1295	0.1355	±	0.0054	4.0%
γ_{af}	1.3614	1.4578	1.4500	1.4861	1.4463	1.4806	1.447	±	0.045	3.1%

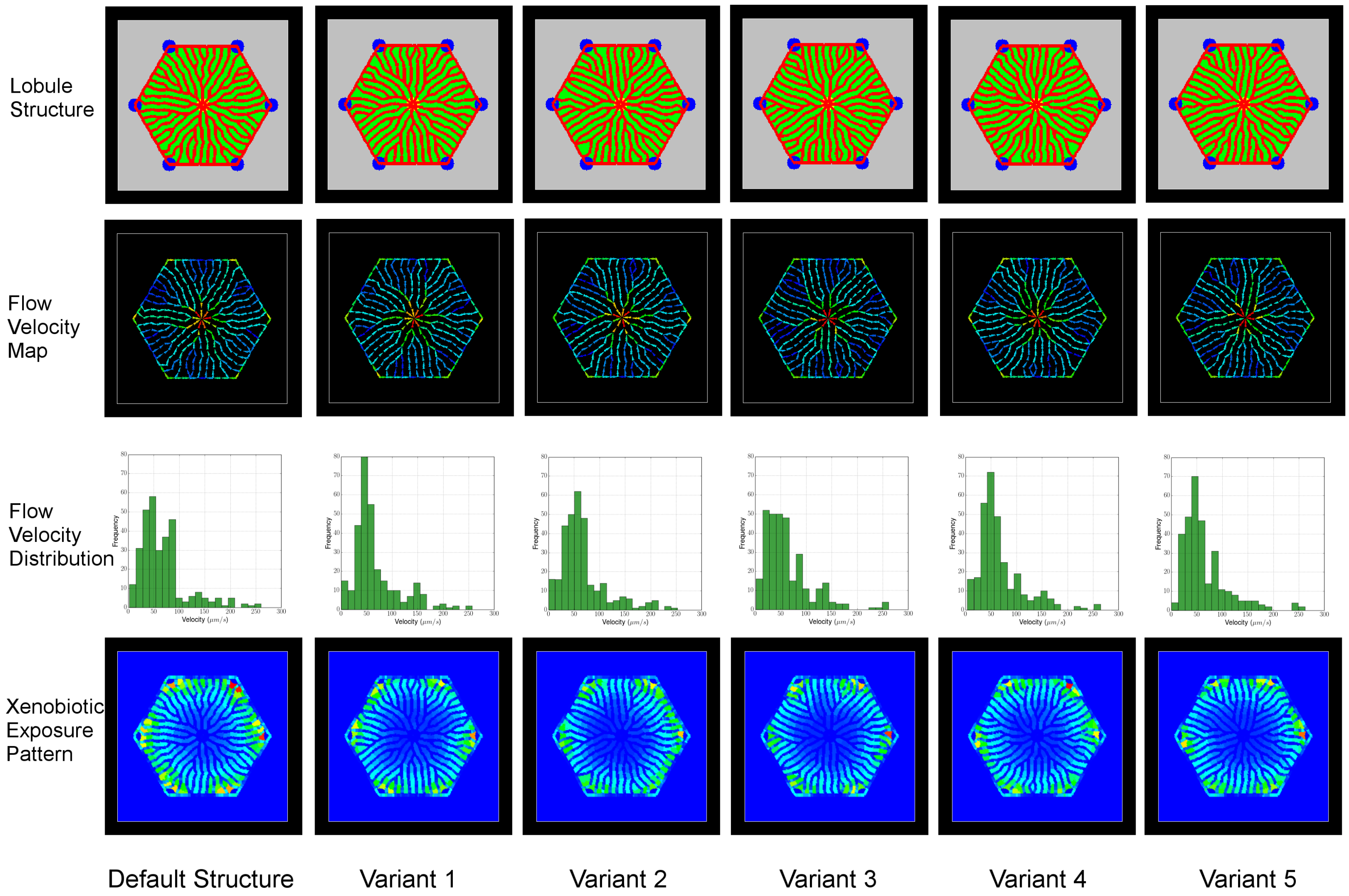

S1 Fig 9. Impact of varying structure of sinusoid network on hemodynamics and hepatic exposure. Columns from left to right show results for 6 different lobule structures. Rows from top to bottom show the lobular structure, flow velocity map, flow velocity distribution and spatial patterns of hepatic exposure to the xenobiotic. All simulations use parameters $\alpha = 10$, $\beta = 10/s$, $D = 10^{-6}\ cm^2/s$ and $CL_{int} = 0.1/s$ for comparison of xenobiotic exposure. Quantitative results from these simulations are summarized in S1 Table 1.

2.3 Flow Calculations and Microdosimetry Simulations with Varied Lobule Size

To explore the effects of lobule size on structural and hemodynamic quantities of the virtual liver lobule, we construct a lobule with a larger PT–to–CV distance of 400 μm. Overall, the descriptors of structural features are very consistent between the default and the larger-size virtual liver lobules, including $\bar{V}_{hep}, \eta_{sin}$ and S_{int} (S1 Table 2). To compare flow velocities across this larger lobule with the default lobule with a PT–to–CV distance of 200 μm, we assume a greater PT to CV pressure drop ($\Delta P = 0.84\ mmHg$) so that the larger lobule has a similar total lobular volume inflow rate $\dot{Q}$. Qualitatively, the larger lobule displays similar spatial pattern of blood flow distribution: (1) radially, flow paths near PT or CV show greater flow velocity than mid-zonal and facial peripheral paths; (2) azimuthally, direct (axial) flow paths from PT to CV typically have greater flow velocity than indirect (facial) flow pathways (S1 Fig 10). In addition, the larger lobule displays similar distributions of flow velocities with a proportionally smaller number of flow paths with magnitude greater than 100 $\mu m/s$. In other words, the number of high flow segments is relatively insensitive to the lobule radius. In terms of descriptors of hydrodynamic features, mean blood flow velocity in the larger lobule is $\bar{v} = 36.7\ \mu m/s$, nearly 50% smaller than that in the default lobule. Smaller flow velocity in the larger lobule results from about 34.6% larger flow resistance ($\frac{\Delta P}{\dot{Q}}$). Apparently, the effective lobular flow resistance doesn't linearly scale with size (PT–to–CV distance) of the lobule, because a greater number of parallel flow pathways in the larger lobule tend to cause a drop in the flow resistance and therefore partially compensate the impact of the increase in size.

Spatial pattern of xenobiotic showed some difference as well in an example simulation with $D = 1 \times 10^{-6} cm^2/s, \alpha = 10, \beta = 10/s$ and $CL_{int} 0.1/s$ respectively. In the simulation with a 400 μm lobule, the xenobiotic was even more predominantly absorbed and metabolized in the periportal region, which led to a very small γ_{cp} and very large γ_{af}.

2.4 Flow Calculations and Microdosimetry Simulations with Varied PT-CV Pressure Drop

We also investigate the effects of larger a PT–to–CV pressure difference on hydrodynamic features of the virtual liver lobule as well as hepatic exposure to the xenobiotic (S1 Table 3). As discussed in the main text, we justified the selection of $\Delta P = 0.6\ mmHg$ as PT–to–CV pressure difference in the virtual mouse liver lobule by comparing calculated average flow velocity and total inflow/outflow volume rate with experimental values. However, it's interesting to examine if the virtual liver lobule with the same architecture (therefore

S1 Table 2. Quantitative Results with Different Lobule Sizes.

Quantity (unit)	200μm lobule	400μm lobule	Ratio
ΔP $(mmHg)$	0.6	0.84	1.40
$\bar{V}_{hep}$ (μm^3)	8017 ± 18	7295	0.91
η_{sin} (%)	12.77 ± 0.07	12.85	1.01
S_{int} $(\mu m^2/\mu m^3)$	0.1224 ± 0.0013	0.1199	0.98
$\bar{v}$ $(\mu m/s)$	65.8 ± 2.0	36.7	0.56
$\dot{Q}$ (pL/s)	94.3 ± 2.9	98.0	1.04
$\bar{c}_{lob}$ $(mmol/L)$	1.17 ± 0.13	0.31	0.26
γ_{cp}	0.1355 ± 0.0054	0.001	
γ_{af}	1.447 ± 0.045	4.886	

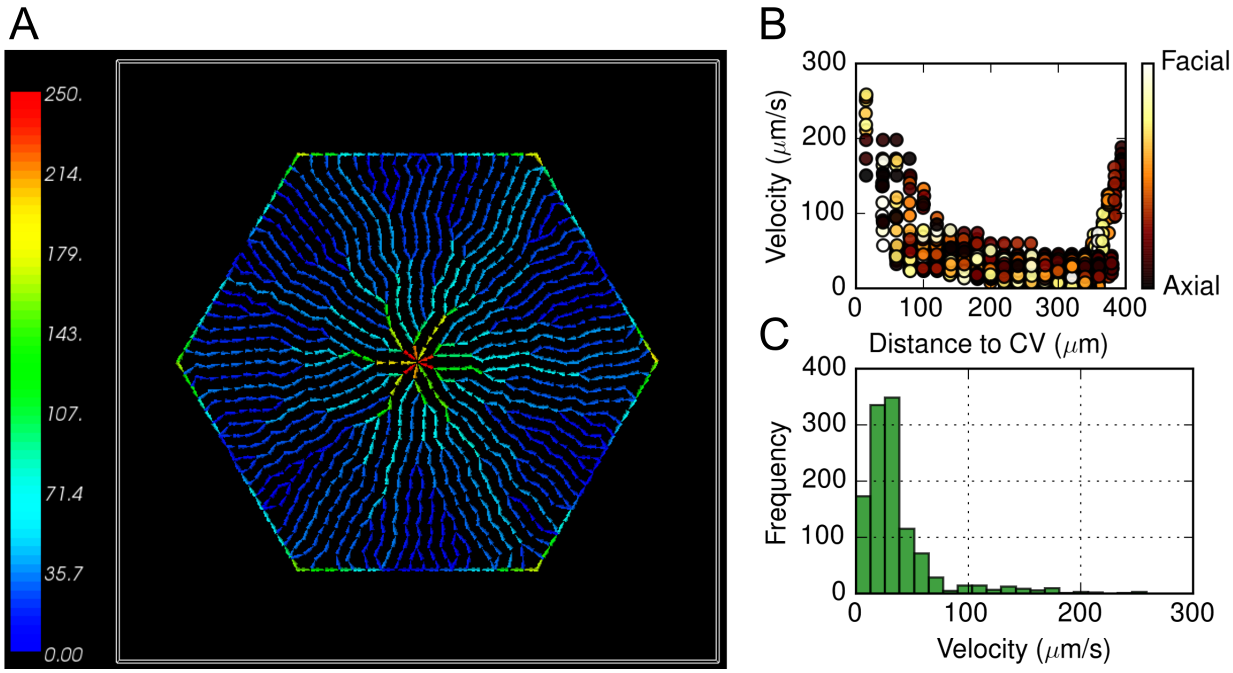

S1 Fig 10. Impact of lobule size on hemodynamics. (A) Calculated flow velocity map in the simulated lobule with a PT–to–CV distance of 400 μm. Warmer color represents greater flow velocity. Color bar has unit $\mu m/s$. (B) Calculated flow velocities in individual sinusoid segments with respect to their distances to central vein. Color codes the segment's angular position. Black indicates axial regions; white indicates facial regions. Note that for a 400 μm lobule the maximum distance from the lobule's center to the center of a face is about 350 μm. (C) Histogram of calculated flow velocities.

the same flow resistance) can represent a rat liver lobule by using a greater PT–to–CV pressure difference. Not surprisingly, both average flow velocity and volumetric flow rates increase proportionately to increase in ΔP (S1 Table 3). Interestingly, a PT-to-CV pressure difference of $\Delta P = 0.6$ $mmHg$ (reported value for rat liver lobule) results in mean flow velocity of $\bar{v} = 337.62$ $\mu m/s$, within the range of reported experimental

values [10–13]. In addition, flow distribution is essentially identical if we scale the velocities by pressure differences (images not shown), because the flow resistances at individual sinusoids segment are the same in these simulations. Therefore, both average flow velocity and volumetric flow rates increase proportionately to change in ΔP.

In terms of hepatic exposure to the xenobiotic, we ran an example simulation with $D = 1 \times 10^{-6} cm^2/s$, $\alpha = 10$, $\beta = 10/s$ and $CL_{int} = 0.1/s$. Simulations with greater ΔP show smaller lobular-wise average xenobiotic concentrations and more uniform lobular distribution of xenobiotic, as both radial and azimuthal discrepancies are closer to unity, indicating smaller hepatic extraction of the xenobiotic due to shorter blood transit time.

S1 Table 3. Quantitative Results with Varied PT–to–CV Pressure Drop ΔP

$\boldsymbol{\Delta P\ (mmHg)}$	**0.6**	**1.2**	**1.8**	**2.4**	**3.0**
$\bar{v}\ (\mu m/s)$	67.52	135.05	202.57	270.10	337.62
$\dot{Q}\ (pL/s)$	97.75	195.50	293.25	390.99	488.74
$\bar{c}_{lob}\ (mmol/L)$	1.426	1.353	1.260	1.118	1.013
γ_{cp}	0.141	0.259	0.391	0.476	0.614
γ_{af}	1.361	1.258	1.167	1.107	1.064

2.5 Descriptors of Hepatic Exposure within a *PIPE* Lobule in Active Transport Only PULSE Simulations

In the main text, we discussed the effect of varying active transport parameters α and β on radial and azimuthal distribution of the xenobiotic within a ***NET*** lobule in ***PULSE*** simulations (S1 Fig 17). In particular, we observed greater peri-central xenobiotic concentration when α and β were small. Here, we ask if this pattern found in ***NET*** lobule was also present ***PIPE*** lobule. In contrast to **NET** lobule, **PULSE** simulations within the ***PIPE*** lobule with small α and small β showed lobular-wise uniform distribution of xenobiotic (S1 Fig 11). This striking comparison emphasized the interpretation in the main text that heterogeneous pattern of blood flow and diverse blood flow transit times contributed to observed preferential peri-central distribution of the xenobiotic within the ***NET*** lobule in ***PULSE*** simulations.

2.6 Hepatic *NET* Exposure to PULSE Xenobiotic

To explore the simplest case of xenobiotic uptake across the simulated lobule we examined the effect of varying the passive transport rate constant, in the absence of active transport and metabolism, for a narrow pulse ($\Delta t = 0.1s$) of xenobiotic. In passive transport-only **PULSE** simulations, xenobiotic was absorbed into

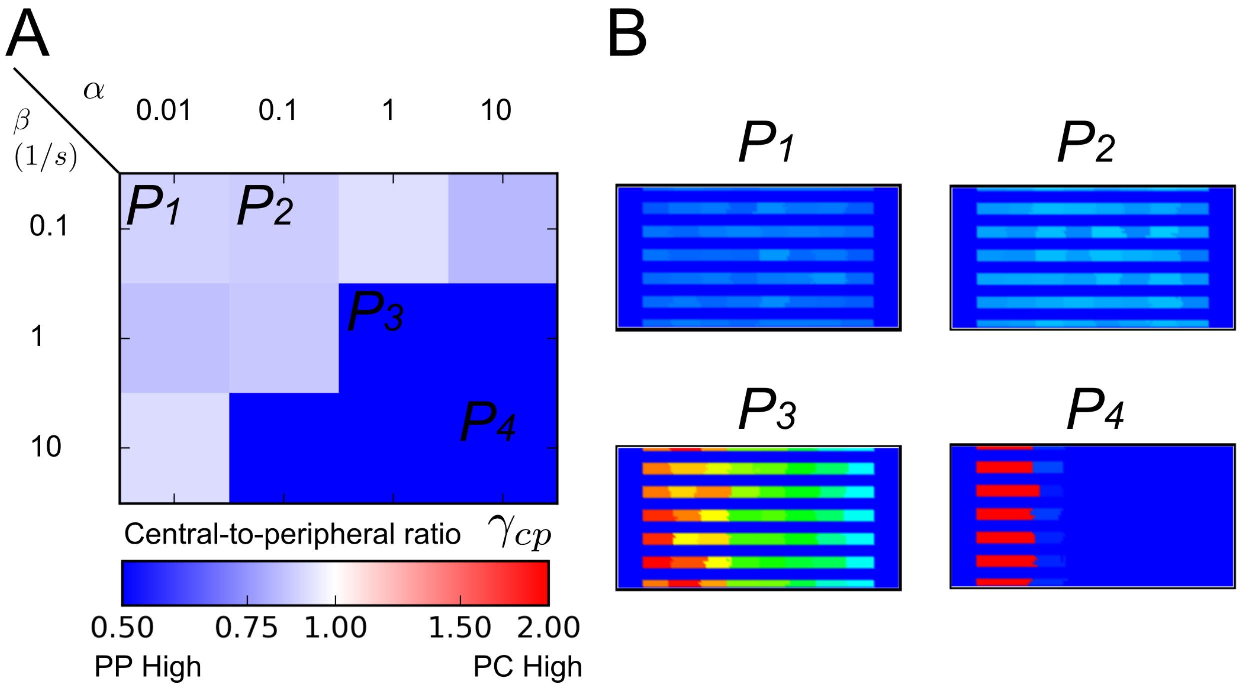

S1 Fig 11. Heat maps of central-to-peripheral ratios within the *PIPE* lobule at $t = 20\ s$ in active transport-only PULSE simulations. (A) Central-to-peripheral ratios γ_{cp} for simulations with 12 different (α, β) pairs. γ_{cp} defines the ratio of the average concentration within radially the most peri-central zone to the average concentration within the most peripheral zone. Warmer color reflects greater peri-central than peripheral concentration. "PP" and "PC" refer to peri-portal and peri-central, respectively. "P_1"–"P_4" correspond to "N_1"–"N_4" (see S1 Fig 17) in terms of (α, β) values. (B) Spatial snapshots of xenobiotic concentration within the ***PIPE*** lobule at 20 seconds in simulations corresponding to parameter domains marked "P_1"–"P_4".

the hepatocytes and eventually cleared from the cells back to the blood (S1 Fig 12). It should be noted that in a particular simulation the same diffusive trans-membrane rate constant was applied to both cell-blood and cell-cell transfers, but this did not guarantee the same diffusive flux, which depends also on variables such as interface area and blood flow velocity.

For a small diffusive trans-membrane rate constant $D = 10^{-7} cm^2/s$ (top row S1 Fig 12) extraction of xenobiotic from the blood is insignificant and most of the pulse exited via the central vein with little extraction into the parenchymal space. We observed penetrating "spike" patterns of high blood concentration of xenobiotic around the portal triads at early time points ($t = 1s$) due to the higher axial blood flow, particularly with small passive rate constants. Slow passive transport resulted in overall low levels of xenobiotic uptake but also reduced the rate that absorbed material was washed back out of the hepatocytes after the end of the input pulse (S1 Fig 12 upper right panel).

For intermediate diffusive trans-membrane rate constant, $D = 10^{-6} cm^2/s$ (middle row S1 Fig 12), uptake

of xenobiotic was notably enhanced compared to the slow passive transport situation. The majority of xenobiotic diffused into hepatocytes before eventually being cleared out and carried away by blood, evidenced by high peripheral concentration at $t = 0.5s$ and $1s$ and a well-maintained radial gradient at $t = 1s$. It is also clear that the diffusive flux between adjacent hepatocytes was insufficient to effectively reduce the gradient. The “spike” patterns were also observed early in the simulation but faded out as the concentration equilibrated between blood and hepatocytes, which ultimately contained xenobiotic of approximately equal concentration during the wash-out phase.

For large diffusive trans-membrane rate constant, $D = 10^{-5}cm^2/s$ (bottom row S1 Fig12), uptake of xenobiotic dominated the advection in blood, indicated by rapid “closure” of the zero-concentration “hole” in the central region via cell-to-cell diffusive transport. Remarkably, unlike simulations with smaller diffusive rate constants, this simulation showed earlier initiation of xenobiotic clearance, likely before $t = 1s$, which implies that the interval between first entrance and first exit could be shorter than the blood transit time at very high cell-to-cell diffusion rates. Furthermore, the “spike” patterns disappear quickly, suggesting that variability in transit time imposed by the network structure had little influence at high diffusive flux. It appears that blood flow was the limiting factor in this situation, since little change in xenobiotic distribution was observed between $t = 2s$ and $t = 5s$.

One characteristic that differentiates the ***PIPE*** model from the ***NET*** model is the possibility of non-uniform *azimuthal* distributions of xenobiotic in the latter case. Azimuthal distribution have an angular dependence, often corresponding to the 6-fold angular symmetry of an ideal liver lobule. As mentioned earlier, the ***NET*** layout has variable path lengths for PT (axial) to CV paths opposed to facial to CV paths. The differences in path lengths affect both the blood flow velocity and the number of hepatocytes that a particular flow path passes. To explore the radial versus azimuthal distributions we divided the ***NET*** lobule into 8 equally spaced radial zones and 12 azimuthal zones, each 30 degrees wide. Radial and azimuthal distributions of xenobiotic at $t = 0.5s$ and $t = 20s$ within the lobule are shown in S1 Fig 13 and S1 Fig 14, respectively. The first time point was selected to explore the uptake phase, while the latter to explore the washout phase. At $t = 0.5s$, a smaller diffusive trans-membrane rate constant resulted in a steeper radial gradient and more axially biased azimuthal distributions. In addition, mean xenobiotic concentrations within radially or azimuthally divided zones were lower in simulations with a smaller diffusive rate constant, indicating lower xenobiotic extraction (S1 Figs 13A, 13C). At $t = 20s$, simulations with an intermediate diffusive trans-membrane rate constant displayed the largest reversed radial gradient of xenobiotic (S1 Fig 14A), because at the peripheral region, hepatocytes and inflow xenobiotic-free blood had the greatest concentration difference and these peripheral cells washed out more quickly. As blood became increasingly

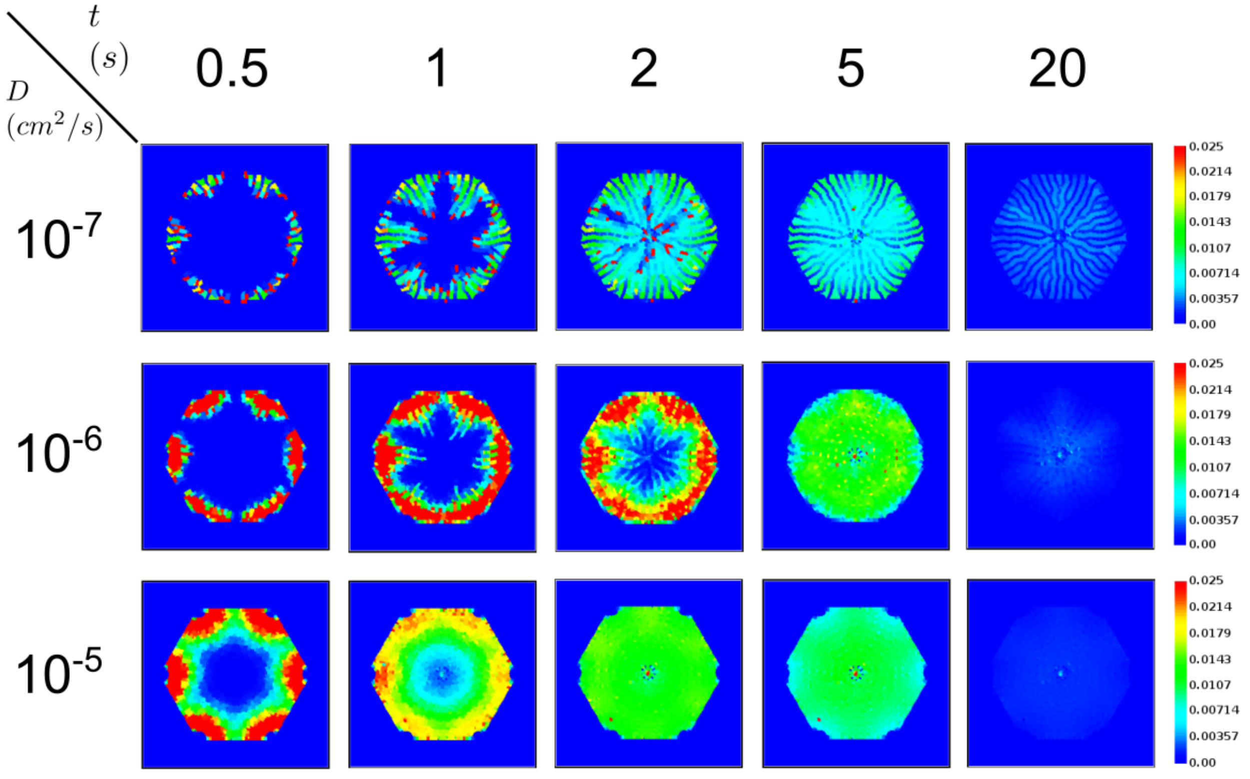

S1 Fig 12. Time progression of xenobiotic concentration in select passive transport-only PULSE simulations. Each row shows time progression of xenobiotic concentration from 0.5 to 20 seconds. Diffusive trans-membrane rate constants are, from top to bottom, 10^{-7}, 10^{-6}, and $10^{-5} cm^2/s$, respectively. Warmer colors reflect higher concentrations (units are $mmol/L$). Note that in some images the xenobiotic is primarily in the sinusoids (e.g., image at upper left), whereas in others it is primarily in the hepatocytes (upper right). Refer to *Supplement 3* for animations.

rich in xenobiotic during transit, clearance slowed for mid-zonal and peri-central cells. If the diffusive rate constant is large, cell-to-cell diffusive transport compensates for this effect. If the diffusive rate constant is small, peripheral cells do not absorb effectively. In terms of azimuthal distribution, simulations with intermediate rate constants also showed the greatest axial-facial discrepancy. As axial pathways corresponded to smaller transit time, clearance of xenobiotic was more efficient. For smaller or larger diffusive rate constants, the axial-facial discrepancy was less because of either slow lobule-wide extraction or enhanced cell-to-cell transfer, respectively.

We next studied the effect of active transport in **PULSE** simulations. In these simulations, cell-to-cell and blood-to-cell diffusive flux is turned off. Without an efflux pathway for the xenobiotic it remains within a given cell once it is actively imported. We are interested in determining if certain regions of the lobule accumulate more xenobiotic and if this accumulation is specific to the ***NET*** model. To explore this, we

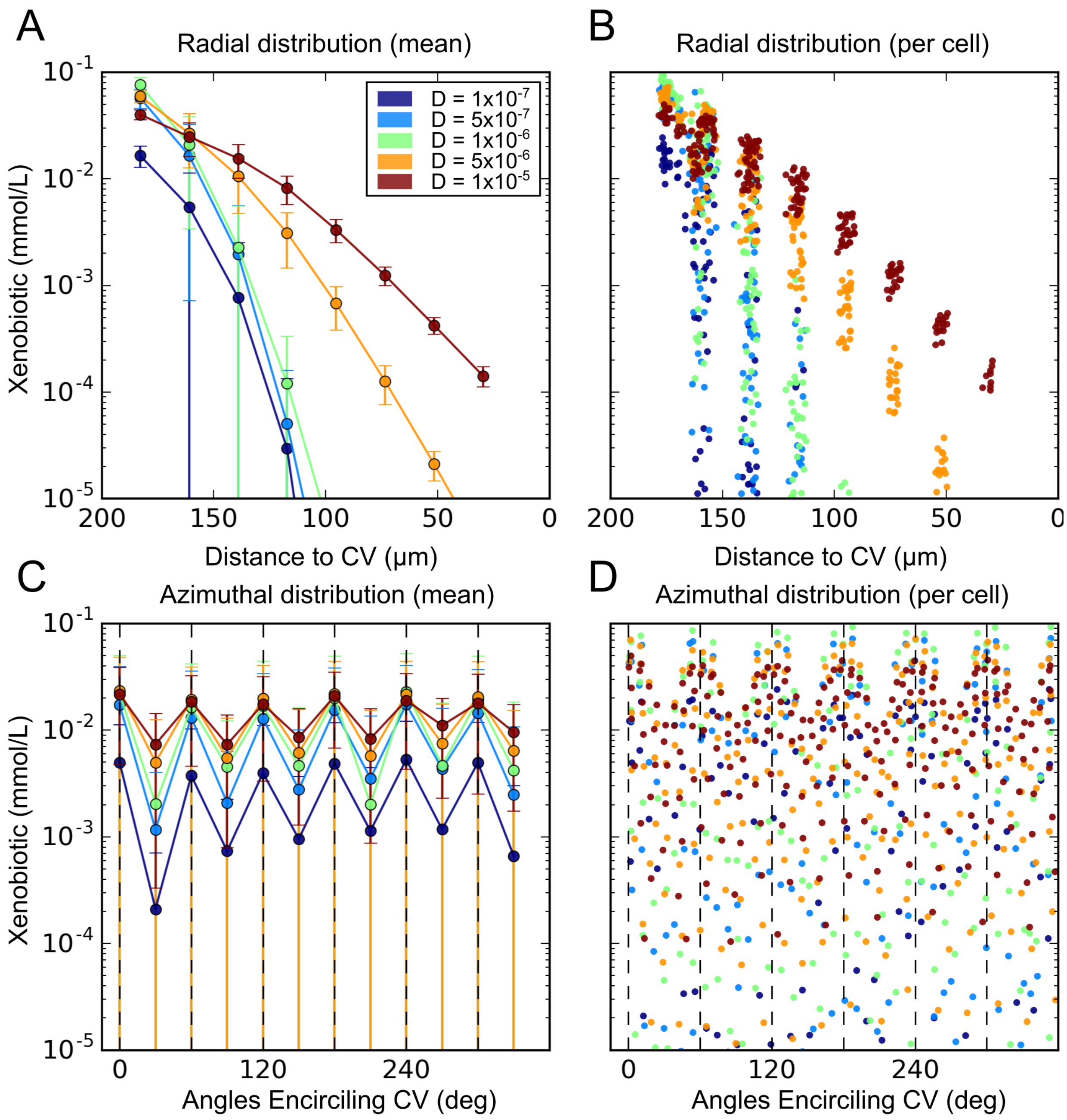

S1 Fig 13. Radial and azimuthal distribution of the xenobiotic the diffusive with respect to trans-membrane rate constant D at $t = 0.5s$ in passive transport-only PULSE simulations. (A) radial distribution of the average xenobiotic concentration in 8 radially-divided zones. (B) radial distribution of xenobiotic concentration within individual hepatocytes. (C) azimuthal distribution of the average xenobiotic concentration in 12 azimuthally-divided zones. (D) azimuthal distribution of xenobiotic concentration within individual hepatocytes. Dashed lines in (C) and (D) indicate angular orientations of six PT–CV axes. Different colors represent different diffusive trans-membrane rate constants. Error bars reflect standard deviations.

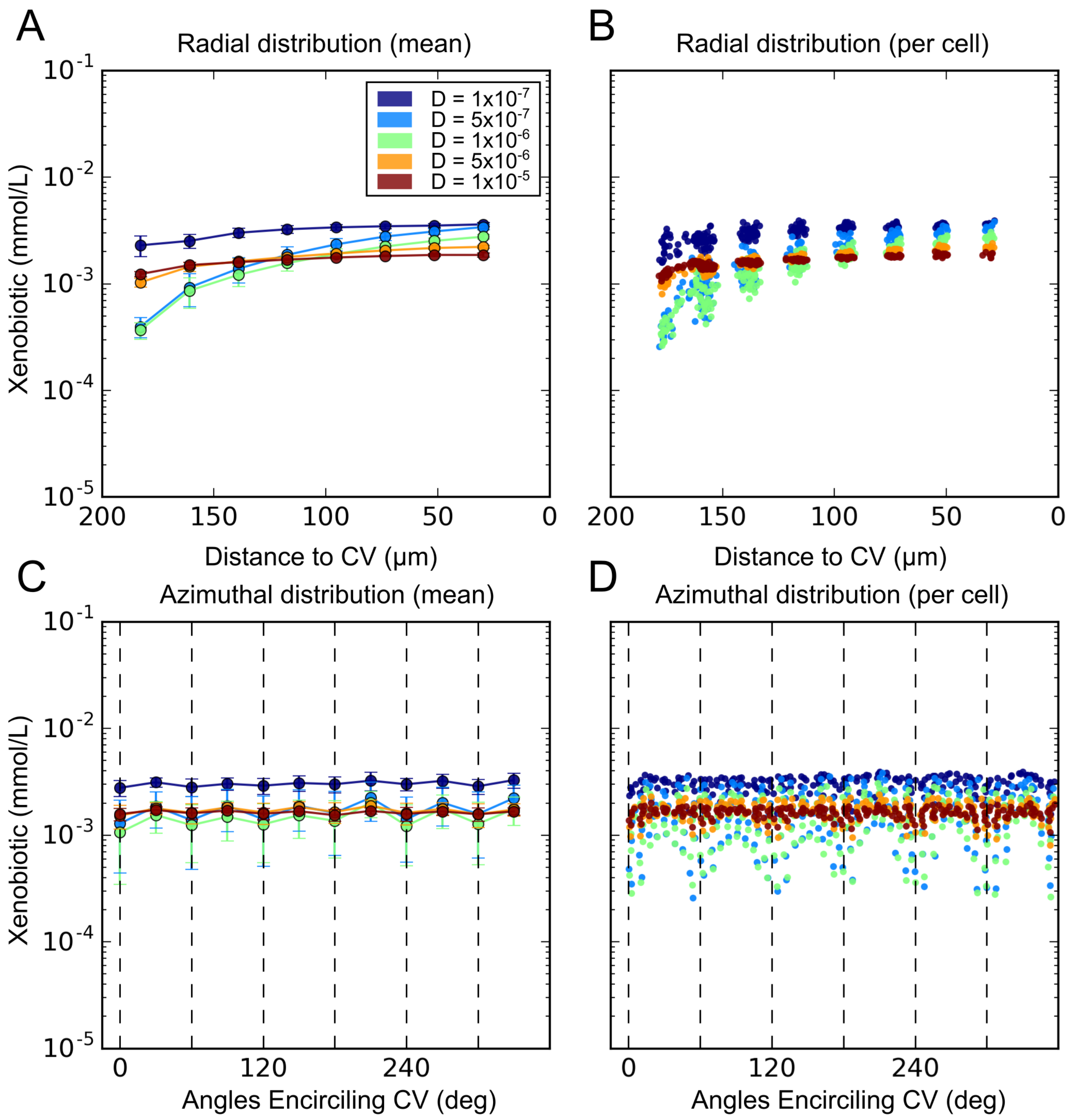

S1 Fig 14. Radial and azimuthal distribution of the xenobiotic the diffusive with respect to trans-membrane rate constant D at $t = 20s$ in passive transport-only PULSE simulations. (A) radial distribution of the average xenobiotic concentration in 8 radially-divided zones. (B) radial distribution of xenobiotic concentration within individual hepatocyte. (C) azimuthal distribution of the average xenobiotic concentration in 12 azimuthally-divided zones. (D) azimuthal distribution of xenobiotic concentration within individual hepatocyte. Dashed lines in (C) and (D) indicate angular orientations of six PT–CV axes. Different colors represent different diffusive trans-membrane rate constants. Error bars reflect standard deviations.

selected four parameter sets consisting of different combinations of α and β as shown in S1 Fig 15. Recall that α is the de-dimensionalized half-saturating concentration for uptake, and β is the ratio of maximum uptake rate to α, which gives a first order rate constant. If $\alpha \ll 1$, uptake of xenobiotic via active transport is almost always saturated and therefore obeys zeroth-order kinetics. Conversely, if $\alpha \gg 1$, uptake it is never saturated. For the intermediate domain $\alpha \sim 1$, some peri-portal cells do, but mid-zonal and peri-central cells don't, have saturated uptake and a near-maximum uptake rate. For simulations with $\beta = 0.1/s$, $\alpha = 0.01$ and $\alpha = 0.1$ showed very similar pattern of xenobiotic distribution. Extraction was small, as indicated by visually intact leading pulses in the sinusoids channels at $t = 0.5, 1, 2s$ (top two rows in S1 Fig 15). The "spike" patterns, introduced by distinct transit times across the lobule, were evident at $t = 1s$. Interestingly, although hepatocytes along the six porto-central axes seemed to experience pulses of xenobiotic sooner, they generally failed to absorb more, as indicated by lower concentrations (cooler color) along these axes at $t = 20s$. Also strikingly and somewhat counter-intuitively, peri-central hepatocytes, on average, accumulated more xenobiotic than peripheral hepatocytes. Both phenomena can be interpreted as a consequence of lobule-wide small uptake rate but different cumulative uptake durations. In **PULSE** simulations where the pulse size is as narrow as $\Delta T = 0.1s$, peri-central hepatocytes have the advantage of encountering a greater number of derived pulses split from the original incoming pulse due to variable transit times imposed by the network structure. In contrast, peri-portal cells experience only a single, short duration pulse. As a result, peri-central cells tended to accumulate more xenobiotic.

Axial and facial viability in xenobiotic absorption resulted from the distinct local flow velocities. Facial hepatocytes, despite fewer encounters with pulses compared to axial cells, had significant duration of xenobiotic in the local blood with some pulses persisting for at least 5 seconds due to the slower blood velocity ($t = 5s$ top two rows in S1 Fig 15). Apparently, increasing pulse width gradually diminishes the relative difference in uptake durations between peri-portal and peri-central hepatocytes, as will be discussed in more detail later. For $\alpha = 1$ and $\beta = 1/s$, the distribution was much different and the pattern was inverted. Peripheral hepatocytes absorbed more xenobiotic than peri-central cells. In the periphery, axial cells accumulated more xenobiotic than facial cells did. For $\alpha = 10$ and $\beta = 10/s$, the distribution was more extreme, and xenobiotic was preferentially absorbed into peripheral, especially peri-portal, hepatocytes.

In order to quantitatively depict the regional accumulation of the xenobiotic in active transport only **PULSE** simulations, we plotted radial and azimuthal distribution of xenobiotic at $t = 20s$ for select simulations with combinations of varying α and $\beta = 1/s$. The set of simulations with $\beta = 1/s$ showed two patterns of hepatic exposure to the xenobiotic: (1) preferentially peri-central and facial; (2) preferentially peri-portal and axial (i.e., peri-portal). In terms of the radial distribution, the xenobiotic accumulated

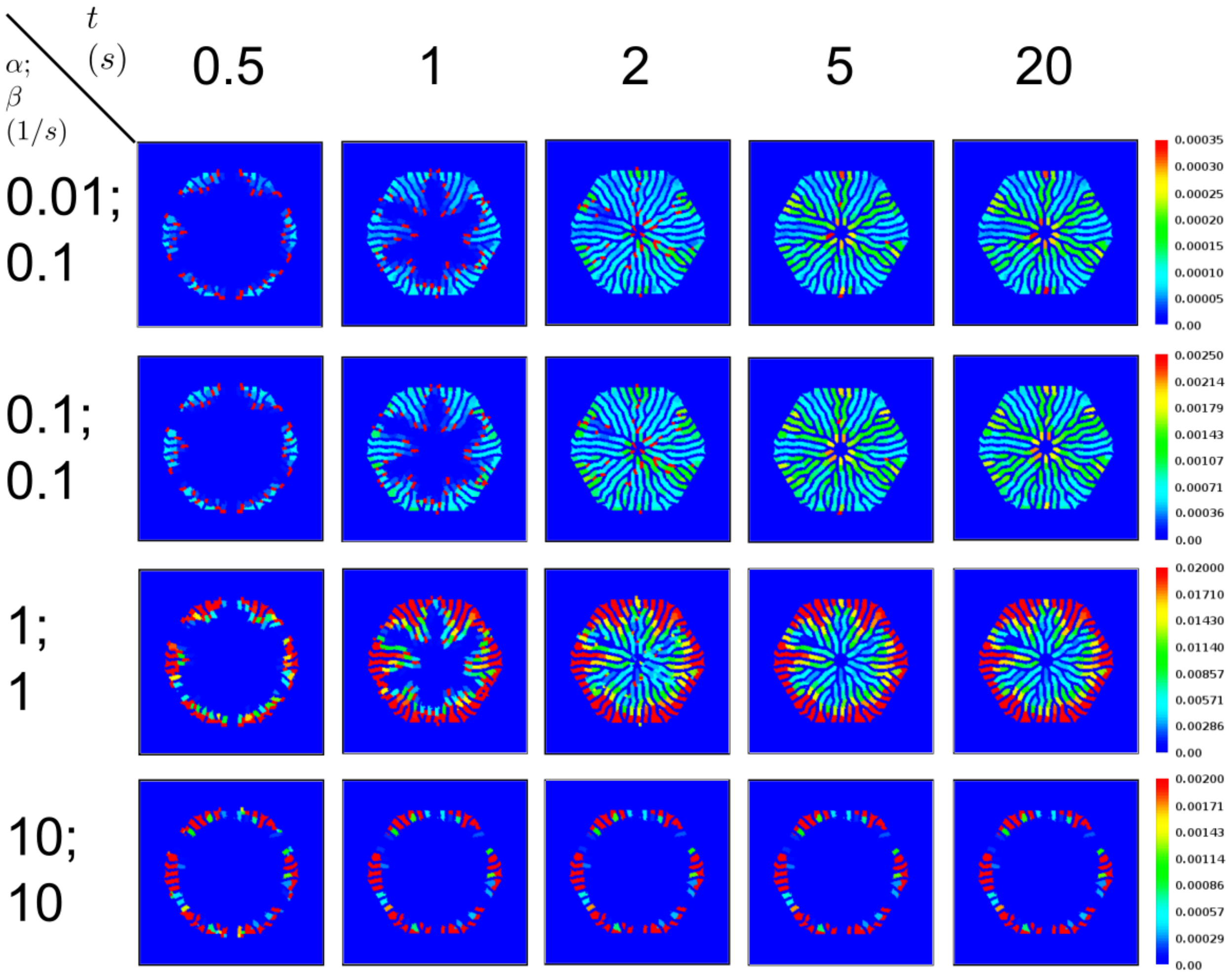

S1 Fig 15. Time progression of xenobiotic concentration in select active transport-only PULSE simulations. Each row shows time progression of xenobiotic concentration from 0.5 to 20 seconds. (α, β) pairs are, from top to bottom, $(0.01, 0.1/s)$, $(0.1, 0.1/s)$, $(1, 1/s)$, $(10, 10/s)$, respectively. Warmer colors indicate higher xenobiotic concentration (units are $mmol/L$). Note that concentrations are plotted at different orders of magnitude in these four simulations. Refer to *Supplement 3* for animations.

predominantly in peri-central hepatocytes for smaller αs (0.01 and 0.1) but in peripheral area for larger α (1 and 10) (S1 Fig 16A–B). In terms of the azimuthal distribution, the xenobiotic primarily accumulated in cells along facial paths for smaller α (0.01 and 0.1) but along porto-central pathways for larger α (1 and 10) (S1 Fig 16C–D). Therefore, in **PULSE** simulations with $\beta = 1/s$, a critical transition between two patterns of hepatic exposure to the xenobiotic occurred at some intermediate value of α between 0.1 and 1. Overall, strong active transport resulted in predominant hepatic exposure to the xenobiotic simultaneously at peripheral and axial regions, namely the peri-portal region, while weak active transport led to preferential hepatic exposure to the xenobiotic simultaneously at pericentral and facial regions.

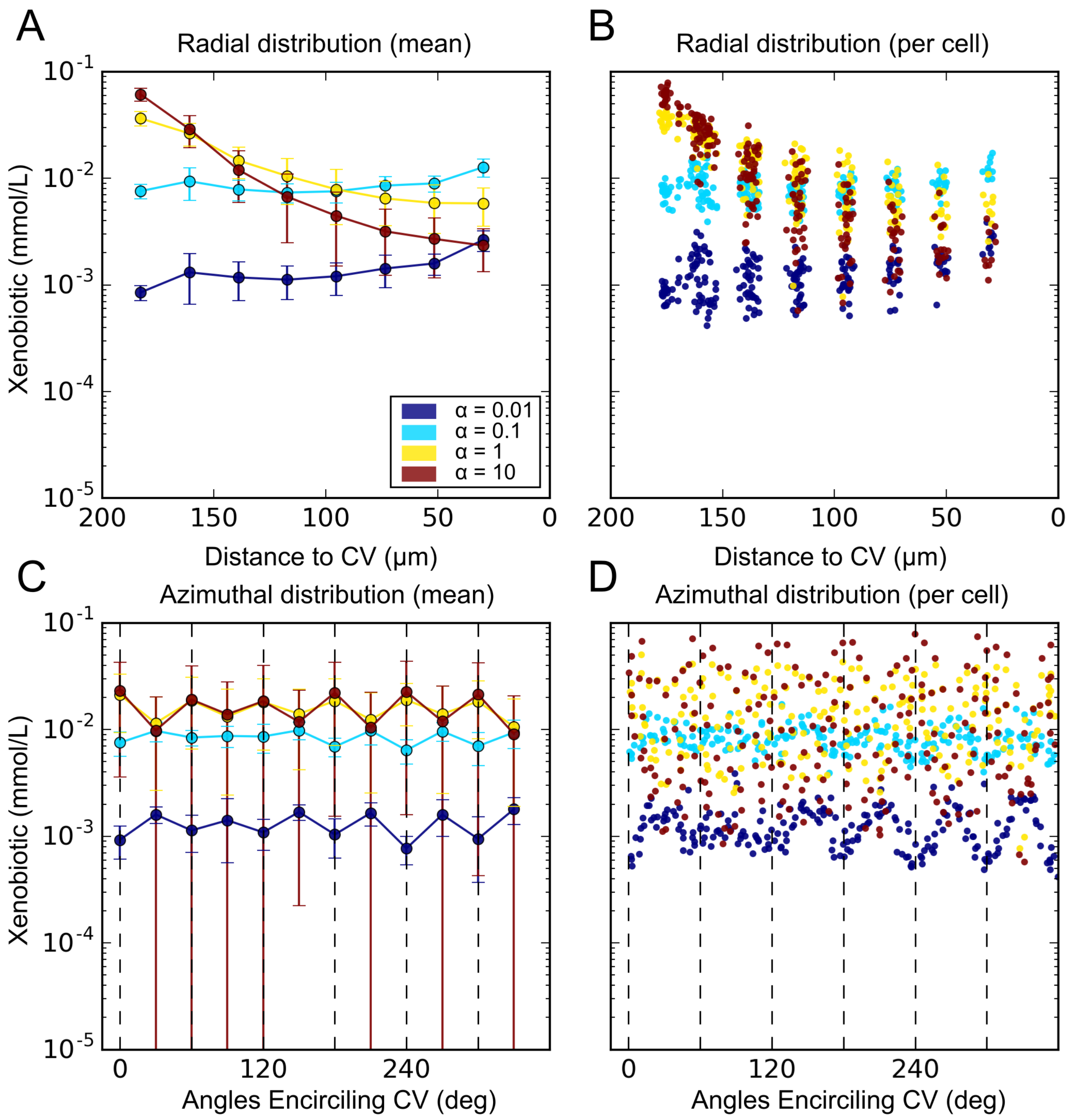

S1 Fig 16. Radial and azimuthal distribution of the xenobiotic with respect to α at $t = 20s$ in active transport-only PULSE simulations with $\beta = 1/s$. (A) radial distribution of the average xenobiotic concentration in 8 radially-divided zones. (B) radial distribution of xenobiotic concentration within individual hepatocyte. (C) azimuthal distribution of the average xenobiotic concentration in 12 azimuthally-divided zones. (D) azimuthal distribution of xenobiotic concentration within individual hepatocytes. Dashed lines in (C) and (D) indicate angular orientations of 6 PT-CV axes. Different colors represent different diffusive trans-membrane rate constants. Error bars reflect standard deviations.

We also employed two descriptors to characterize radial and azimuthal distributions of xenobiotic in the active transport-only simulations at $t = 20s$, as illustrated using heat maps in S1 Fig 17. The descriptor for radial distribution, termed the central-to-peripheral ratio γ_{cp}, defines the ratio of the average xenobiotic concentration at the peri-central-most radial zone to that at the peripheral-most radial zone (S1 Fig 17A). Scenarios with $\gamma_{cp} > 1.25$, $0.75 < \gamma_{cp} \leq 1.25$ and $\gamma_{cp} \leq 0.75$ reflect preferentially peri-central, radially uniform and preferentially peripheral exposure to the xenobiotic, respectively. The descriptor for azimuthal distribution, termed the axial-to-facial ratio γ_{af}, defines the ratio of the average xenobiotic concentration at the six axial azimuthal zones to that at the six facial azimuthal zones (S1 Fig 17B). Scenarios with $\gamma_{af} > 1.25$, $0.75 < \gamma_{af} \leq 1.25$ and $\gamma_{af} \leq 0.75$ reflect preferentially axial, azimuthally uniform and preferentially facial exposure to the xenobiotic, respectively.

For example, for the simulation with $\alpha = 1$ and $\beta = 1$, the rightmost data point of the yellow curve divided by the leftmost data point of the same curve in S1 Fig 16A gave the central-to-peripheral ratio γ_{cp} for this (α, β) pair. The mean value of the six data points of the yellow curve overlapping with dashed lines divided by the mean value of the other six data points of the same curve in S1 Fig 16C gave the axial-to-facial ratio γ_{af}. In S1 Fig 17A–B, the vertical axis from top to bottom reflects increasing maximum uptake rate and the horizontal axis from left to right reflects increasing half-saturating concentration of active transport. Both central-to-peripheral and axial-to-facial heat maps showed a "two-domain" pattern with the boundary between the two domains being the secondary diagonal of the (α, β) parameter matrices (S1 Fig 17A–B). This result echoes the xenobiotic distribution in select simulations as depicted in S1 Fig 16. Very small α alone, or combination of small α and small β, resulted in greater peri-central concentration radially and slightly greater facial concentration azimuthally. In contrast, simulations with large α and large β showed greater peripheral accumulation radially and greater axial accumulation azimuthally. An alternative description of the latter case is simply greater peri-portal accumulation. S1 Fig 17C shows corresponding spatial snapshots of xenobiotic distribution for four select domains "N_1"–"N_4".

In contrast to ***NET*** simulations, ***PIPE*** simulations didn't show higher peri-central xenobiotic concentrations in any combination of α and β (*Supplement 2.5*). In addition, As briefly mentioned earlier, we also examined whether (α, β)-domain specific pre-centrally preferential xenobiotic distribution was preserved when the pulse size was increased, as detailed in *Supplement 2.6.* We found that an increase in **PULSE** size alone was sufficient to alter the spatial pattern observed in active transport only **PULSE** simulations with weak active transport, from predominantly centri-lobular to lobular-wide uniform xenobiotic distribution. While this section focuses on describing spatial patterns of hepatic exposure to the xenobiotic, *Supplement 2.7* further discusses the overall hepatic exposure as reflected by lobular partition of the

xenobiotic in the hepatocytes versus in the blood within the virtual liver lobule.

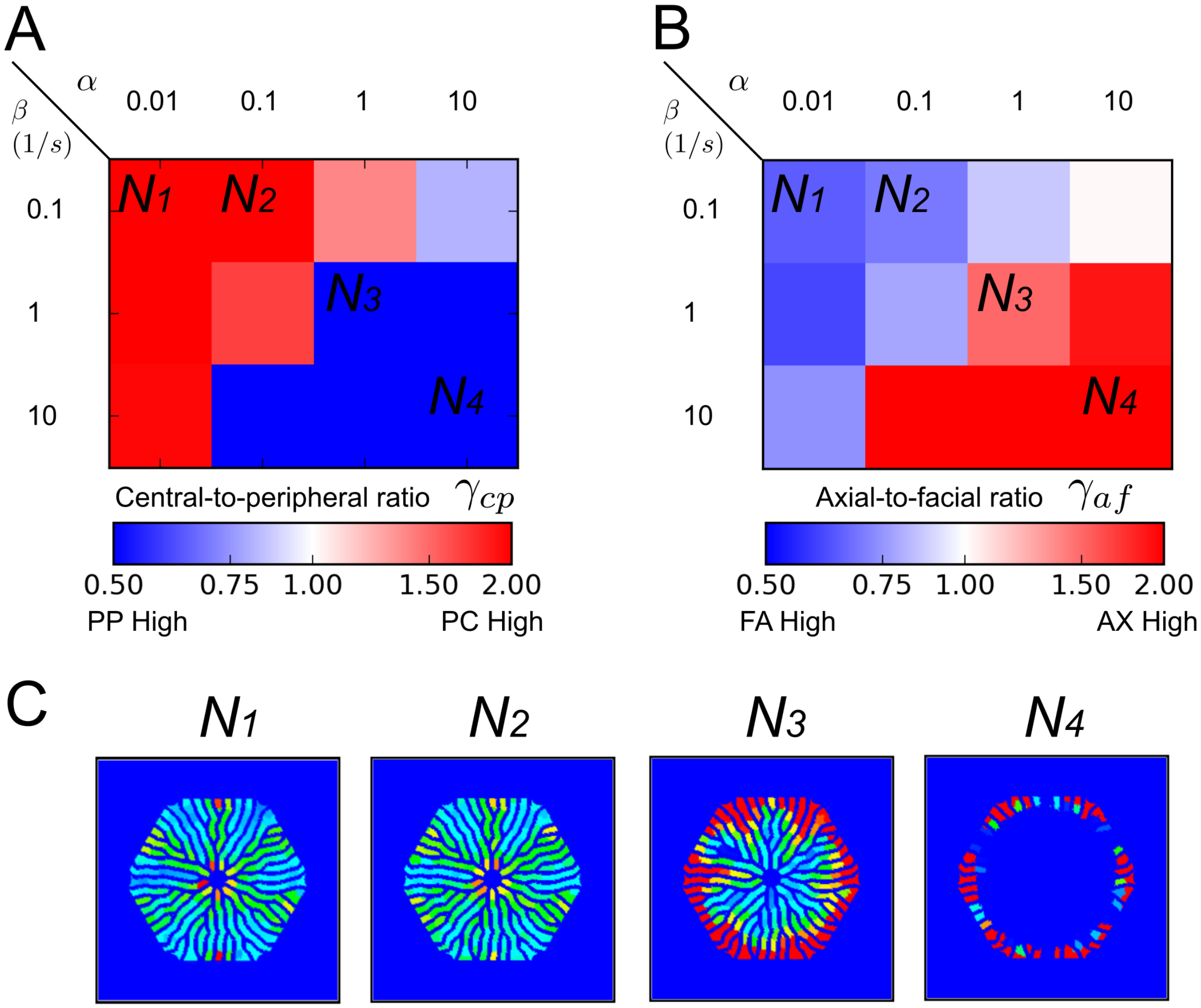

S1 Fig 17. Heat maps of central-to-peripheral and axial-to-facial ratios at t = 20 s in active transport-only PULSE simulations. (A) Central-to-peripheral ratios γ_{cp} for simulations with 12 different (α, β) pairs. γ_{cp} defines the ratio of the average concentration within radially the most peri-central zone to the average concentration within the most peripheral zone. Warmer color reflects greater peri-central than peripheral concentration. "PP" and "PC" refer to peri-portal and peri-central, respectively. (B) Axial-to-facial ratios γ_{af} for simulations with 12 different (α, β) pairs. γ_{af} defines the ratio of the average xenobiotic concentration within azimuthally the six axial zones by the average concentration within the six facial zones. Warmer color reflects greater axial than facial concentration. "FA" and "AX" refer to facial and axial, respectively. Blocks marked "N_1"–"N_4" in (A) and (B) correspond to (α, β) pairs used for simulations in S1 Fig 15. (C) Spatial snapshots of xenobiotic concentration at 20 seconds in simulations corresponding to parameter domains marked "N_1"–"N_4". Refer to S1 Fig 15 for color bars.

2.7 Descriptors of Hepatic Exposure within a *NET* lobule in Active Transport Only PULSE simulations with Varying Pulse Sizes

In the main text, we discussed spatial patterns of xenobiotic exposure in active transport only **PULSE** simulations with a **PULSE** size of $\Delta T = 0.1s$ (S1 Fig 17). A striking feature of the spatial pattern observed for weak active transport and short pulse duration was that xenobiotic preferentially accumulated in centri-lobular region. We interpreted this phenomenon as a consequence of variation in *cumulative uptake durations* of cells located in different lobular regions, and postulate that an increase of **PULSE** size would diminish the difference in cumulative uptake durations and thus alter radial variation in hepatic exposure to the xenobiotic.

We tested this prediction by gradually increasing the pulse size from $0.2s$ to $10s$ in **PULSE** simulations and created heat maps of the radial descriptor γ_{CP} (S1 Fig 18). As predicted, with pulse size increasing from $0.2s$ to $10s$, (α, β) domains of active transport that led to higher peri-central xenobiotic concentration gradually regressed to a smaller size at the top left corner of heat maps, and displayed smaller magnitudes of discrepancy. Interestingly, the dark red color in the heat maps, that represents a large discrepancy in hepatic exposure to the xenobiotic where peri-central xenobiotic accumulation is at least twice as large as peripheral accumulation, disappeared as the pulse size increased from $1s$ to $2s$, which coincided with typical blood transit time of $1s \sim 2s$ (for example, compare $t = 1s$ and $t = 2s$ snapshots at top row in S1 Fig 15). These results implied that in active transport-only **PULSE** simulations with lobular-wise weak active transport, a peri-central hepatocyte likely accumulated at least twice as much xenobiotic as a peripheral cell did on average in the virtual liver lobule, if the pulse size of the inflow xenobiotic was narrower than the typical transit time of blood flow in the simulated sinusoid network.

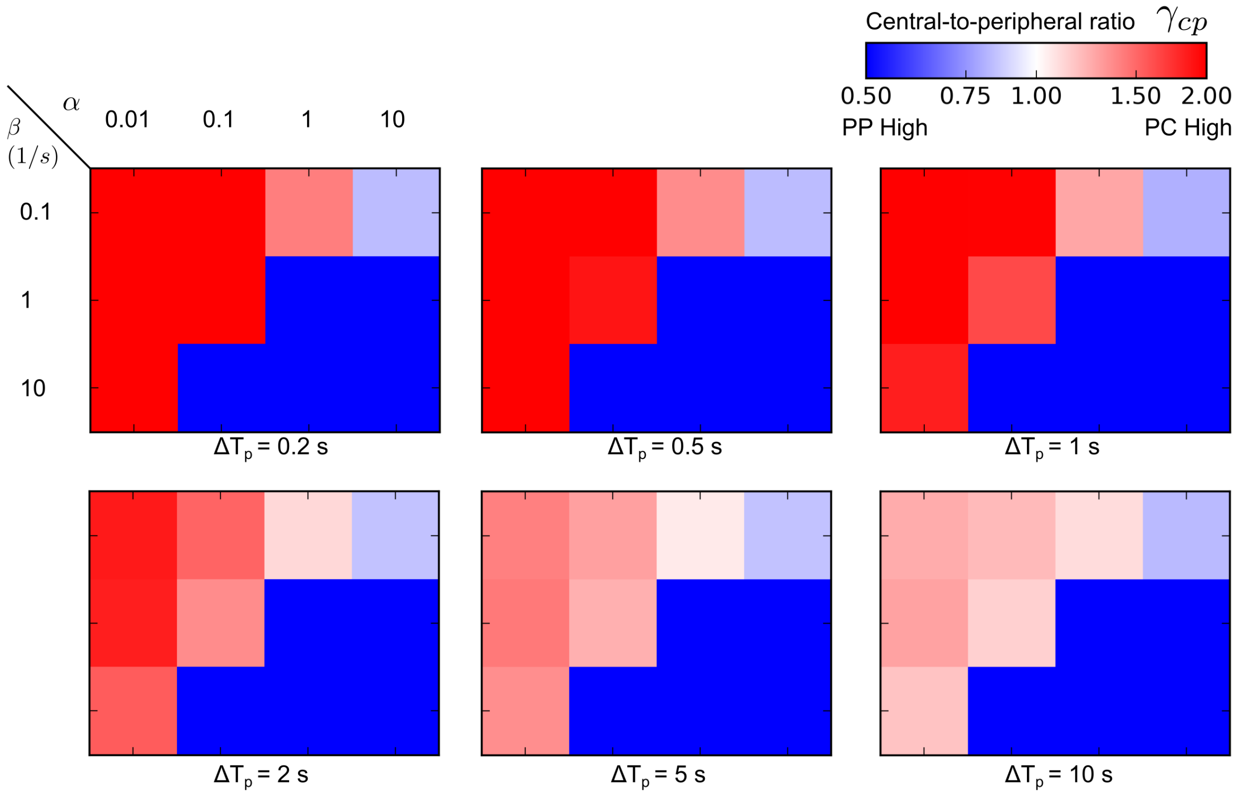

S1 Fig 18. Heat maps of central-to-peripheral ratios within a *NET* lobule at $t = 20$ *s* in active transport-only PULSE simulations with different pulse sizes. Sizes of the pulse, ΔT_p, indicated below each heat map, range from 0.2 to 10 seconds. Refer to S1 Fig 17 for details on the range of (α, β) pairs. "PP" and "PC" refer to peri-portal and peri-central, respectively.

2.8 Lobular Partition of the Xenobiotic in PULSE Simulations

The main text discusses the spatial patterns of hepatic exposure to the xenobiotic via simulations of microdosimetry of xenobiotics with diverse strengths of transport and metabolism. These simulations also produced relative quantities of the xenobiotic in the hepatocytes versus in the blood within the virtual liver lobule, which was an indicator of overall lobular exposure to the xenobiotic.

For passive transport only **PULSE** simulations, xenobiotic quantity partitioned in the parenchyma had a two-phase temporal pattern: uptake phase and clearance phase (S1 Fig 19A). Simulation with larger diffusive trans-membrane rate constant D displayed faster uptake and typically slower clearance. An obvious exception was the simulation with smallest diffusion coefficient, which showed slowest uptake and slowest clearance. Another feature of these curves was peak magnitudes observed for different diffusion coefficients. The largest three diffusion coefficients seemed to have the same peak magnitudes despite different peak times, suggesting identical equilibrium states of blood-parenchymal partition of xenobiotic. For the two smallest diffusion coefficients, peak magnitude was lower due to weaker diffusive transport.

For active transport only **PULSE** simulations, only inward uptake was permissive and all simulations arrived at and maintained at a plateau of xenobiotic concentration within hepatocytes (S1 Fig 19B-D). For a particular β, larger α caused greater extraction of the xenobiotic (S1 Fig 19B). For a particular α, larger β resulted in greater extraction and earlier equilibration of xenobiotic (S1 Fig 19C-D). For extremely large α and β, all incoming xenobiotic ended up within the hepatocytes (S1 Fig 19D).

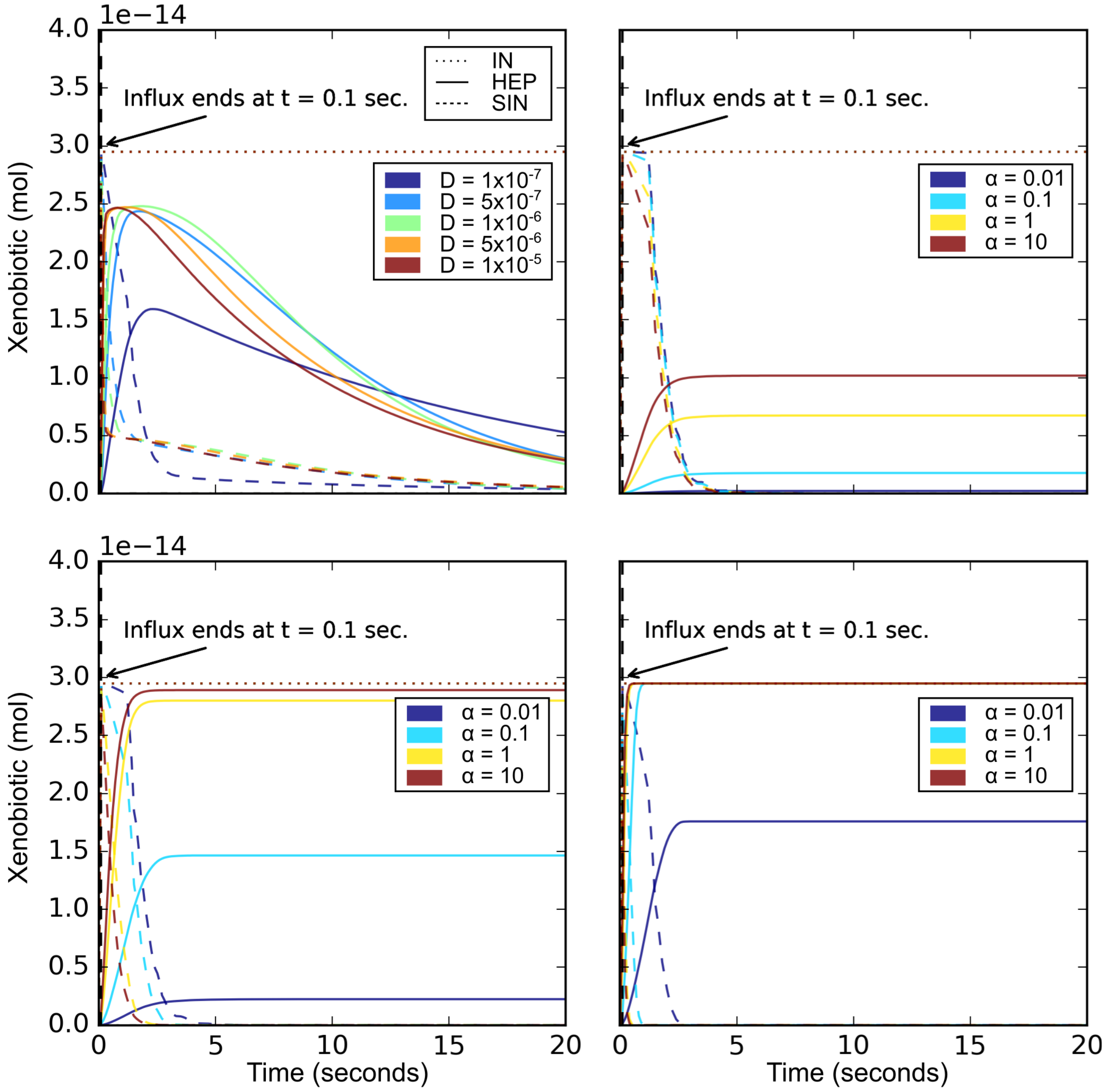

S1 Fig 19. Lobular partition of the xenobiotic over time in passive transport-only and active transport-only PULSE simulations. (A) Lobular partition of xenobiotic in passive transport-only simulations. Different colors represent different diffusive trans-membrane rate constants. (B)-(D) Lobular partition of xenobiotic in active transport-only simulations with $\beta = 0.1/s$, $\beta = 1/s$ and $\beta = 10/s$ respectively. Different colors represent different α. Line styles in (A)-(D) represent partitions. Dotted lines refer to the total incoming mass of xenobiotic; dashed lines refer to mass in the blood; and solid lines refer to mass in all hepatocytes.

2.9 Descriptors of Hepatic Exposure within a *NET* lobule in Active Transport Only PERSISTENT simulations

Heat maps of radial and azimuthal descriptors of hepatic exposure to the xenobiotic in active transport only **PERSISTENT** simulations showed strong resemblance to active transport only **PULSE** simulations except that weak active transport didn't cause predominant distribution of xenobiotic in the former case (S1 Fig 20). These **PERSISTENT** simulations were effectively the extreme cases of simulations with varying pulse sizes, as discussed above (S1 Fig 18). To echo the conclusions above, these simulations suggested that *preferential centrilobular accumulation of xenobiotic occurred only if pulse size was so narrow that centrilobular cells would acquire cumulatively longer duration of exposure to xenobiotic due to spatial arrangement of hepatocytes and vessels within the* ***NET*** *lobule.* Increasing pulse size in **PULSE** simulation or introducing xenobiotic continuously in **PERSISTENT** simulation could partially or entirely eliminate this radial discrepancy.

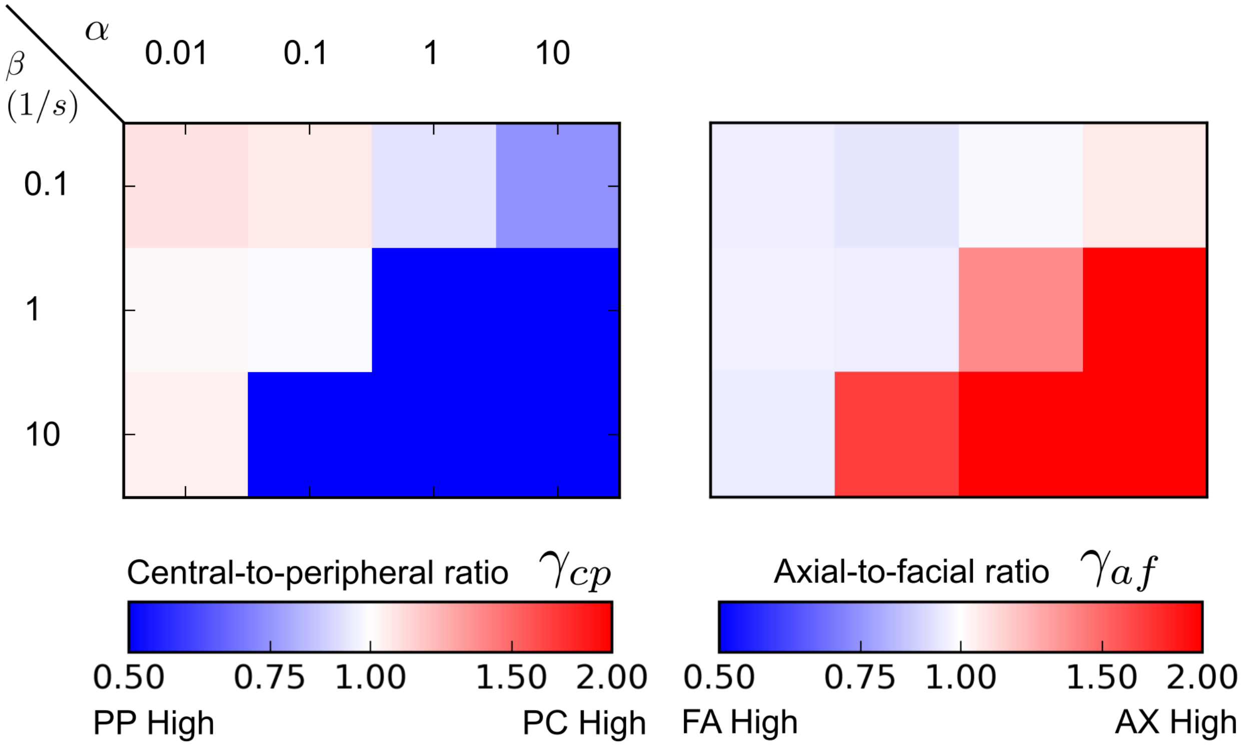

S1 Fig 20. Heat maps of central-to-peripheral and axial-to-facial ratios within a *NET* lobule at $t = 20$ s in active transport only PERSISTENT simulations. (A) central-to-peripheral ratio. (B) axial-to-facial ratio. Refer to S1 Fig 17 for details on the range of (α, β) pairs.

2.10 Variability in Calculated Hepatic Exposure from Replicate Simulations

Simulation of xenobiotic transport and metabolism is stochastic in the model because in each time step the processing order of the cells is random. To test the model's sensitivity to this stochastic component, we ran 50 replicates using the same lobule configuration and examined the variability in lobular-wise mean concentration of the xenobiotic ($\bar{c}_{lob}$) and central-to-peripheral ratio (γ_{cp}) at 20 seconds using select parameter sets from **PERSISTENT** simulations. These three parameter sets were; "simA" which had only active transport with $\alpha = 1$ and $\beta = 1/s$; "simAP" which had active/passive transport with $\alpha = 1$, $\beta = 1/s$ and $D = 10^{-6}\ cm^2/s$; "simAPM" which had active/passive transport and metabolism with $\alpha = 1$, $\beta = 1/s$, $D = 10^{-6}\ cm^2/s$ and $CL_{int} = 1/s$.

Average values and standard deviations from the 50 replicate simulations for the three parameter sets are listed in S1 Table 4. In all cases the results were very consistent, with standard deviations in the two outputs usually smaller than 1%. This indicates that the stochastic component of the model has only minimal affect on the model's outputs and that multiple runs for a given lobule geometry and parameter set are not needed.

S1 Table 4. Variability in Replicate Simulations: Average and Standard Deviations for 50 Replicate Simulations

	Lobular Mean Concentration ($\bar{c}_{lob}$) ($mmol/L$)	Central-to-Peripheral Ratio (γ_{cp})
simA	2.7354 ± 0.0090 (0.33%)	0.2080 ± 0.0078 (3.8%)
simAP	1.7311 ± 0.0022 (0.13%)	0.8594 ± 0.0045 (0.52%)
simAPM	0.1572 ± 0.0004 (0.25%)	0.0164 ± 0.0003 (1.8%)

2.11 Lobular Partitioning of the Xenobiotic in PERSISTENT Simulations

In the main text, we discussed how the interplay between active/passive transport and metabolism affected spatial patterns of hepatic exposure to the xenobiotic. We continue to discuss here their effects on lobular partition of the xenobiotic. One marked difference in temporal pattern of xenobiotic partition in these **PERSISTENT** simulations compared to the **PULSE** simulations is the linearly incremental dotted lines that represent continuous inflow of xenobiotic with constant concentration.

In simulations without metabolism (S1 Fig 21), for particular β and D, larger α resulted in greater xenobiotic partition in the parenchyma at a given time point. For particular α and D, larger β resulted in greater xenobiotic partition in the parenchyma at a given time point. For particular α and β, larger D resulted in smaller xenobiotic partition in the parenchyma at a given time point.

S1 Fig 22 and S1 Fig 23 depict lobular partitions of the xenobiotic in **PERSISTENT** simulations with

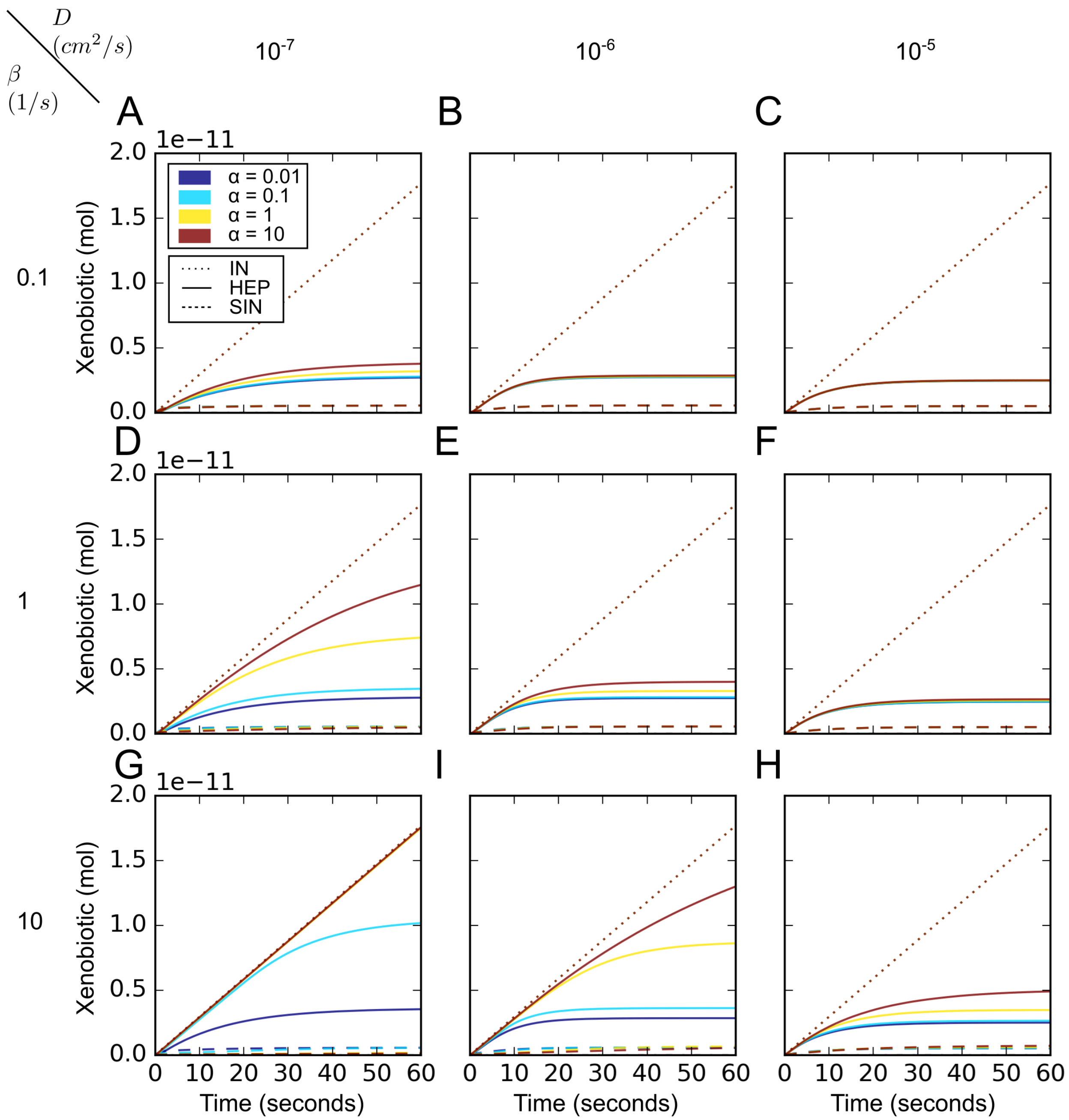

S1 Fig 21. Lobular partition of the xenobiotic over time in PERSISTENT simulations without metabolism. βs are, from top to bottom, $0.1/s$, $1/s$, and $10/s$, respectively. Ds are, from left to right, $1 \times 10^{-7}\ cm^2/s$, $1 \times 10^{-6}\ cm^2/s$, and $1 \times 10^{-5}\ cm^2/s$, respectively. Different colors represent different αs. Line styles represent partitions. Dotted lines refer to the cumulative incoming quantities of the xenobiotic; dashed lines refer to quantities in the blood; and solid lines refer to quantities in all hepatocytes. Only he first 60 seconds of simulation were shown.

varying strengths of active/passive transport and metabolism with $CL_{int} = 0.01/s$ and $CL_{int} = 0.1/s$, respectively. For a particular CL_{int}, variation in α, β and D had similar effects on lobular partition of the

xenobiotic to those observed in simulations without metabolism. For particular α, β and D, larger CL_{int} resulted in smaller partition of the xenobiotic in the parenchyma at a given time point.

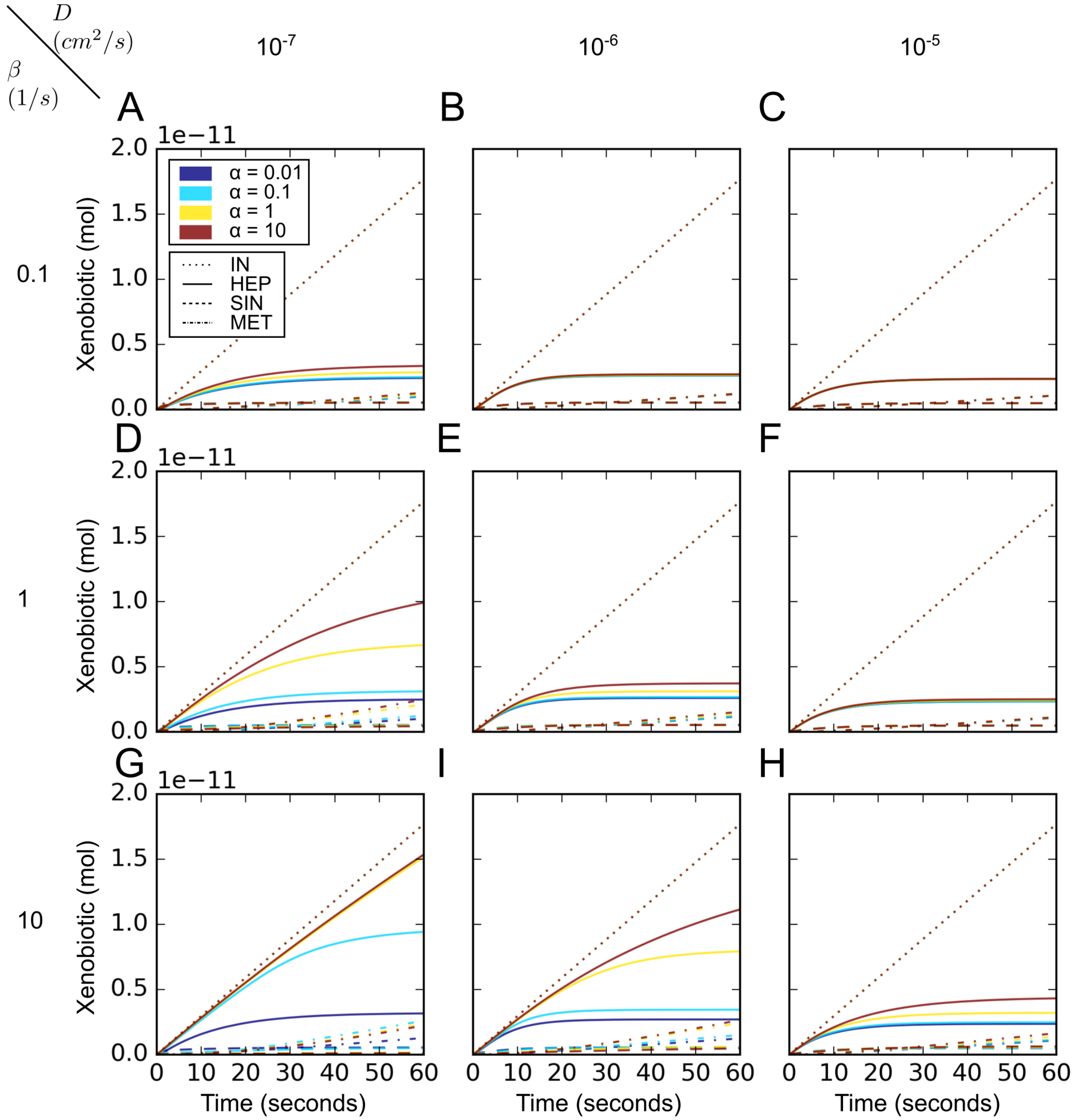

S1 Fig 22. Lobular partition of the xenobiotic over time in PERSISTENT simulations with both active/passive transports and metabolism ($CL_{int} = 0.01/s$). βs are, from top to bottom, $0.1/s$, $1/s$, and $10/s$, respectively. Ds are, from left to right, $1 \times 10^{-7}\ cm^2/s$, $1 \times 10^{-6}\ cm^2/s$, and $1 \times 10^{-5}\ cm^2/s$, respectively. Different colors represent different αs. Line styles represent partitions. Dotted lines refer to the cumulative incoming quantities of the xenobiotic; dashed lines refer to quantities in the blood; and solid lines refer to quantities in all hepatocytes. Only he first 60 seconds of simulation were shown.

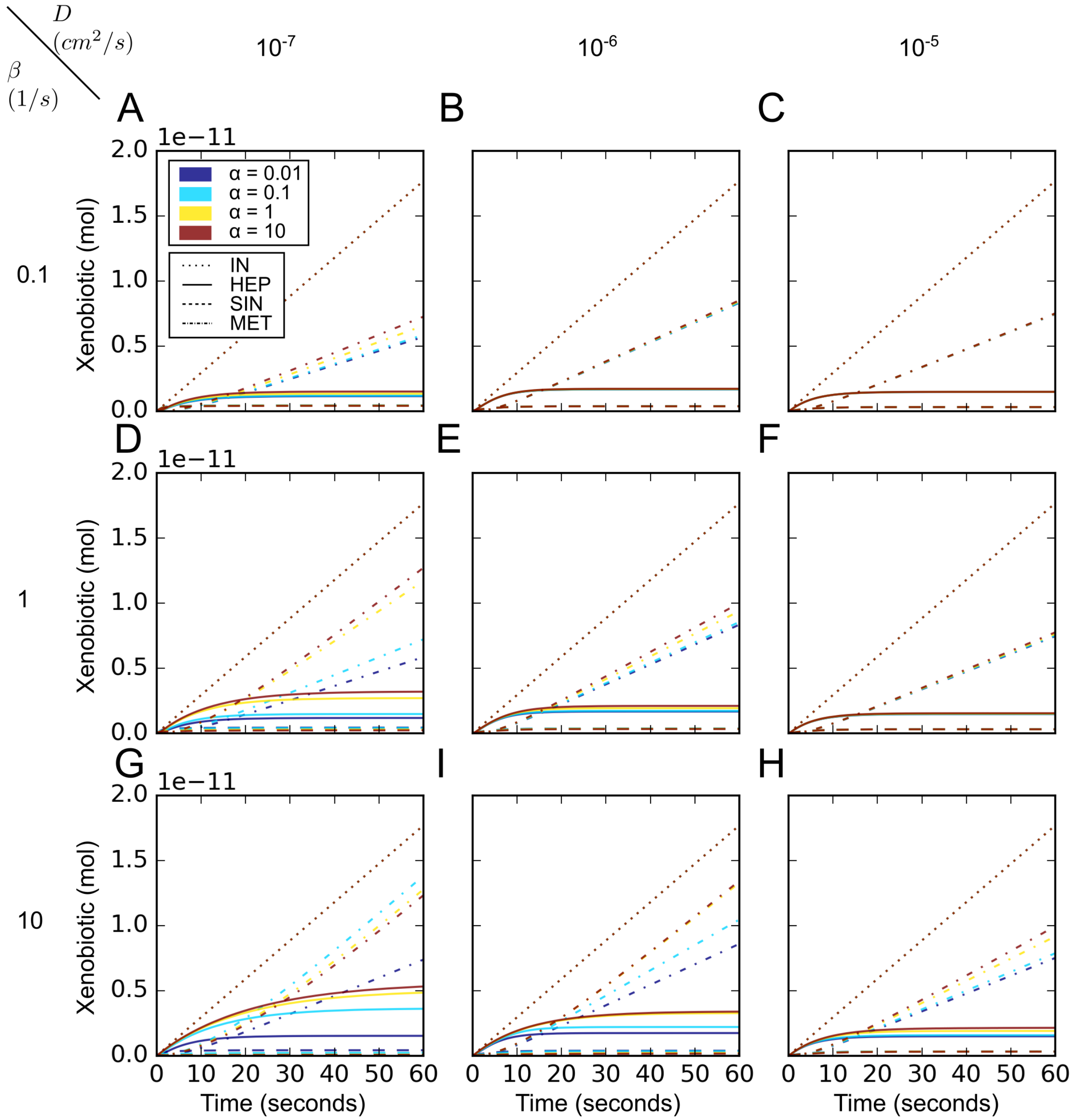

S1 Fig 23. Lobular partition of the xenobiotic over time in PERSISTENT simulations with both active/passive transports and metabolism ($CL_{int} = 0.1/s$). βs are, from top to bottom, $0.1/s$, $1/s$, and $10/s$, respectively. Ds are, from left to right, 1×10^{-7} cm^2/s, 1×10^{-6} cm^2/s, and 1×10^{-5} cm^2/s, respectively. Different colors represent different αs. Line styles represent partitions. Dotted lines refer to the cumulative incoming quantities of the xenobiotic; dashed lines refer to quantities in the blood; and solid lines refer to quantities in all hepatocytes. Only he first 60 seconds of simulation were shown.

3 Animations

We describe below parameter sets used for animations (S1 Animations) listed in S1 Table 5. We named the animation files by concatenating simulation type, structure type and process type(s). For instance, **PULSE_PIPE_AT.avi** refers to **PULSE** simulation with only active transport (_AT) within a ***PIPE*** lobule, while **PERSISTENT_NET_APM_CL1.avi** refers to **PERSISTENT** simulation with active/passive transport and metabolism within a ***NET*** lobule.

In animation files **PULSE_PIPE_PT.avi** and **PULSE_NET_PT.avi**, we included animations of five simulations with different diffusive trans-membrane rate constants ($D = 1 \times 10^{-7}\ cm^2/s$, $D = 5 \times 10^{-7}\ cm^2/s$, $D = 1 \times 10^{-6}\ cm^2/s$, $D = 5 \times 10^{-6}\ cm^2/s$, $D = 1 \times 10^{-5}\ cm^2/s$). Some snapshots collected from three of the five simulations in **PULSE_NET_PT.avi** were shown in S1 Fig 12.

In animation files **PULSE_PIPE_AT.avi** and **PULSE_NET_AT.avi**, we included animations of four simulations with (α, β) pairs; $(0.01, 0.1/s)$, $(0.1, 0.1/s)$, $(1, 1/s)$, $(10, 10/s)$. Snapshots collected from ***NET*** simulations were shown in Fig 5 of the main text. Snapshots collected from ***PIPE*** simulations were shown in S1 Fig 11 of supplementary text.

In animation file **PERSISTENT_NET_AP.avi**, we included animations of six simulations with selected (α, β, D) combinations; $(0.01, 0.1/s, 1 \times 10^{-7}\ cm^2/s)$, $(0.01, 0.1/s, 1 \times 10^{-6}\ cm^2/s)$, $(0.01, 0.1/s, 1 \times 10^{-5}\ cm^2/s)$, $(10, 10/s, 1 \times 10^{-7}\ cm^2/s)$, $(10, 10/s, 1 \times 10^{-6}\ cm^2/s)$, $(10, 10/s, 1 \times 10^{-5}\ cm^2/s)$. Snapshots at $t = 20\ s$ collected from these six simulations were shown as first and fourth row in Fig 5 of the main text.

In animation file **PERSISTENT_NET_APM_CL0.1.avi**, we included animations of six simulations with $CL_{int} = 0.1/s$ and selected (α, β, D) combinations; $(0.01, 0.1/s, 1 \times 10^{-7}\ cm^2/s)$, $(0.01, 0.1/s, 1 \times 10^{-6}\ cm^2/s)$, $(0.01, 0.1/s, 1 \times 10^{-5}\ cm^2/s)$, $(10, 10/s, 1 \times 10^{-7}\ cm^2/s)$, $(10, 10/s, 1 \times 10^{-6}\ cm^2/s)$, $(10, 10/s, 1 \times 10^{-5}\ cm^2/s)$. Snapshots at $t = 20\ s$ collected from these six simulations were shown as second and fifth row in Fig 5 of the main text.

In animation file **PERSISTENT_NET_APM_CL1.avi**, we included animations of six simulations with $CL_{int} = 1/s$ and selected (α, β, D) combinations; $(0.01, 0.1/s, 1 \times 10^{-7}\ cm^2/s)$, $(0.01, 0.1/s, 1 \times 10^{-6}\ cm^2/s)$, $(0.01, 0.1/s, 1 \times 10^{-5}\ cm^2/s)$, $(10, 10/s, 1 \times 10^{-7}\ cm^2/s)$, $(10, 10/s, 1 \times 10^{-6}\ cm^2/s)$, $(10, 10/s, 1 \times 10^{-5}\ cm^2/s)$. Snapshots at $t = 20\ s$ collected from these six simulations were shown as third and sixth row in Fig 5 of the main text.

S1 Table 5. Animations for selected **PULSE** and **PERSISTENT** simulations.

File Name	Type	Structure	Active Transport	Passive Transport	Metabolism
PULSE_PIPE_AT.avi	PULSE	PIPE	Y	N	N
PULSE_PIPE_PT.avi	PULSE	PIPE	N	Y	N
PULSE_NET_AT.avi	PULSE	NET	Y	N	N
PULSE_NET_PT.avi	PULSE	NET	N	Y	N
PERSISTENT_NET_AP.avi	PERSISTENT	NET	Y	Y	N
PERSISTENT_NET_APM_CL0.1.avi	PERSISTENT	NET	Y	Y	Y
PERSISTENT_NET_APM_CL1.avi	PERSISTENT	NET	Y	Y	Y

4 Source Files

The model is implemented in CompuCell3D package (http://compucell3d.org/). Tutorials for using CompuCell3D can be downloaded from the website. We include in *Supplementary Information* the source files for a ***NET*** simulation with parameter values $\alpha = 1,\ \beta = 1/s,\ D = 10^{-6}\ cm^2/s$ and $CL_{int} = 1/s$ as a compressed file named S1 Code (see the subfolder "[CODE]_alpha1_beta1_D1e-6_CLint1.zip"). A list of files in this compressed file are briefly described in S1 Table 6. All simulations described in the *Main Text* can be derived by adjusting parameter values in "pDictLiverXeno.csv" as described in S1 Table 7. Source files for other simulations appearing only in *Supplementary Information* are available upon request.

S1 Table 6. Files included in the zip file S1 Code.

File Name	Description
LIVER_LOBULE_APAP_SBML_PBPK.py	Main python script including configurations for simulation and definitions for basic cell properties; **Essential for model setup in CompuCell3D**
LIVER_LOBULE_APAP_SBML_PBPKSteppables.py	Steppable python script including user defined functions for additional cell properties and behaviors; **Essential for model setup in CompuCell3D**
APAP_GSH_Liver_Metab_Sul_Glu.xml	SBML file to describe xenobiotic metabolism with only one reaction turned on for simulations in this manuscript; Called from LIVER_LOBULE_APAP_SBML_PBPK-Steppables.py
pDictLiverXeno.csv	CSV file of model parameters including those adjustable to derive ***NET*** simulations (see S1 Table 7); Called from LIVER_LOBULE_APAP_SBML_PBPK-Steppables.py
csv_2015-10-12/adj-lobule-rings-seed-12.csv	CSV file of connection information of sinusoid network; Called from LIVER_LOBULE_APAP_SBML_PBPK-Steppables.py
csv_2015-10-12/loc-lobule-rings-seed-12.csv	CSV file of node locations of sinusoid network; Called from LIVER_LOBULE_APAP_SBML_PBPK-Steppables.py
csv_2015-10-12/cell.csv	CSV file of cell center locations; Called from LIVER_LOBULE_APAP_SBML_PBPK-Steppables.py

S1 Table 7. Parameters in "pDictLiverXeno.csv" for generation of ***NET*** simulations.

Parameter Name	Range of Values (units)	Description
DplAPAP & DtAPAP	1x10^{-7}, 5x10^{-7}, 1x10^{-6}, 5x10^{-6}, 1x10^{-5} (cm^2/s)	Diffusive (passive) trans-membrane rate constant for xenobiotic transfer (see D in Table 2 of main paper); Equivalent to D in the main paper
KmAtAPAP	0.01, 0.1, 1, 10 ($mmol/L$)	Half saturating concentration of active uptake (see α in Table 2 of main paper); Equivalent to K_m^{AT} in the main paper
VmaxAtAPAP	0.1, 1, 10 ($1/s$) x KmAtAPAP	Maximum active uptake rate (see β in Table 2 of main paper); Equivalent to V_{max}^{AT} in the main paper
Km_PhaseIIEnzGlu_APAP	0.1, 1, 10, 100 ($1/s$)	Maximum metabolic rate (see CL_{int} in Table 2 of main paper); Equivalent to V_{max}^{M} in the main paper
pulseDura	0.1, 1000 (s)	Duration of incoming xenobiotic pulse; 0.1 used for all **PULSE** simulations, 1000 used for all **PERSISTENT** simulations

5 References

1. National Research Council. Guide for the Care and Use of Laboratory Animals. Washington, DC: The National Academies Press; 2011. Available from: https://www.nap.edu/catalog/12910/guide-for-the-care-and-use-of-laboratory-animals-eighth.

2. Clendenon SG, Young PA, Ferkowicz M, Phillips C, Dunn KW. Deep Tissue Fluorescent Imaging in Scattering Specimens Using Confocal Microscopy. Microscopy and Microanalysis. 2011;17(4):614–617.

3. Schindelin J, Arganda-Carreras I, Frise E, Kaynig V, Longair M, Pietzsch T, et al. Fiji: an open-source platform for biological-image analysis. Nat Meth. 2012;9(7):676–682. doi:10.1038/nmeth.2019.

4. Cardona A, Saalfeld S, Schindelin J, Arganda-Carreras I, Preibisch S, Longair M, et al. TrakEM2 Software for Neural Circuit Reconstruction. PLOS ONE. 2012;7(6):1–8. doi:10.1371/journal.pone.0038011.

5. Hammad S, Hoehme S, Friebel A, von Recklinghausen I, Othman A, Begher-Tibbe B, et al. Protocols for staining of bile canalicular and sinusoidal networks of human, mouse and pig livers, three-dimensional reconstruction and quantification of tissue microarchitecture by image processing and analysis. Archives of toxicology. 2014;88(5):1161–83. doi:10.1007/s00204-014-1243-5.

6. Blouin A, Bolender RP, Weibel ER. Distribution Of Organelles And Membranes Between Hepatocytes And Nonhepatocytes In The Rat Liver Parenchyma. The Journal of Cell biology. 1977;72:441–455.

7. Swat MH, Thomas GL, Belmonte JM, Shirinifard A, Hmeljak D, Glazer JA. Multi-scale modeling of tissues using CompuCell3D. Methods in cell biology. 2012;110:325–366. doi:citeulike-article-id:11149386 doi: 10.1016/b978-0-12-388403-9.00013-8.

8. Pries AR, Secomb TW, Gessner T, Sperandio MB, Gross JF, Gaehtgens P. Resistance to blood flow in microvessels in vivo. Circulation Research. 1994;75(5):904–915. doi:10.1161/01.RES.75.5.904.

9. Pries AR, Secomb TW. Microvascular blood viscosity in vivo and the endothelial surface layer. American Journal of Physiology - Heart and Circulatory Physiology. 2005;289(6):H2657–H2664. doi:10.1152/ajpheart.00297.2005.

10. Koo A, Liang IYS, Cheng KK. The Terminal Hepatic Microcirculation In The Rat. Quarterly Journal of Experimental Physiology and Cognate Medical Sciences. 1975;60(4):261–266. doi:10.1113/expphysiol.1975.sp002320.

11. Koo A, Liang IY. Microvascular filling pattern in rat liver sinusoids during vagal stimulation. J Physiol. 1979;295:191–199.

12. Shibayama Y, Nakata K. Localization of increased hepatic vascular resistance in liver cirrhosis. Hepatology. 1985;5(4):643–648. doi:10.1002/hep.1840050421.

13. Komatsu H, Koo A, Guth PH. Leukocyte flow dynamics in the rat liver microcirculation. Microvascular Research. 1990;40(1):1 – 13. doi:http://dx.doi.org/10.1016/0026-2862(90)90002-9.
